# Trends in Persuasive Technologies for Physical Activity and Sedentary Behavior: A Systematic Review

**DOI:** 10.3389/frai.2020.00007

**Published:** 2020-04-28

**Authors:** Noora Aldenaini, Felwah Alqahtani, Rita Orji, Srinivas Sampalli

**Affiliations:** ^1^Faculty of Computer Science, Dalhousie University, Halifax, NS, Canada; ^2^Department of Computer Science, Imam Abdulrahman Bin Faisal University, Dammam, Saudi Arabia; ^3^Department of Computer Science, King Khalid University, Abha, Saudi Arabia

**Keywords:** persuasive technology, persuasive strategies, behavior theory, targeted audience, targeted outcomes, physical activity, sedentary behavior, health

## Abstract

Persuasive technology (PT) is increasingly being used in the health and wellness domain to motivate and assist users with different lifestyles and behavioral health issues to change their attitudes and/or behaviors. There is growing evidence that PT can be effective at promoting behaviors in many health and wellness domains, including promoting physical activity (PA), healthy eating, and reducing sedentary behavior (SB). SB has been shown to pose a risk to overall health. Thus, reducing SB and increasing PA have been the focus of much PT work. This paper aims to provide a systematic review of PTs for promoting PA and reducing SB. Specifically, we answer some fundamental questions regarding its design and effectiveness based on an empirical review of the literature on PTs for promoting PA and discouraging SB, from 2003 to 2019 (170 papers). There are three main objectives: (1) to evaluate the effectiveness of PT in promoting PA and reducing SB; (2) to summarize and highlight trends in the outcomes such as system design, research methods, persuasive strategies employed and their implementaions, behavioral theories, and employed technological platforms; (3) to reveal the pitfalls and gaps in the present literature that can be leveraged and used to inform future research on designing PT for PA and SB.

## 1. Introduction

In recent years, our way of life has become increasingly sedentary, which is a significant public health issue. Sedentary behavior (SB) is defined as any awake behavior that has an energy expenditure ≤1.5 metabolic equivalent (METs). This may include a sitting, lying, or reclining posture such as watching television and working at a desk [1]. When we compare our life to previous generations, it is clear that our life has become more sedentary. For example, some individuals are spending more time in environments that limit physical activity (PA) and require prolonged sitting. A sedentary lifestyle is associated with health complications such as obesity, diabetes, cancer, and cardiovascular diseases, among other conditions [2]. Thus, reducing SB and increasing PA has been the focus of much PT. There is a need to understand how persuasive technology (PT) has been used to promote health and prevent disease by targeting certain behaviors in the individual that promote their PA and reduce SB.

Over the years, considerable research has designed and used PT to promote PA and discourage SB. Thus, it is important to understand and evaluate the effectiveness of these PT at achieving their intended outcome of reducing the health risks associated with a sedentary lifestyle by promoting PA.

Therefore, in this paper we aim to achieve three main objectives: (1) to evaluate the effectiveness of PTs used to promote PA and reduce SB; (2) to summarize and highlight trends in the outcomes such as system design, research methods, persuasive strategies employed and their implementations, behavioral theories, and employed technological platforms; and (3) to reveal pitfalls and gaps in the present literature that could be leveraged and used to inform the design of PTs targeting physical activity. To achieve this, we conducted a systematic review of 170 research papers to identify and evaluate the effectiveness of PT for promoting PA and discouraging SB using the Persuasive System Design (PSD) model [3] as shown in Table 1.

**Table 1:** Principles “strategies” of Persuasive System Design (PSD) Model [3].

**Descriptions of PSD model strategies “Principles”**	
**Primary task support**	
Reduction	The system has to decrease effort and strain that users consume when doing their target behavior. The reduction principle can be achieved by reducing a complex behavior into simple and easy tasks for users.
Tunneling	The system has to guide users in the attitude change process or experience by providing opportunities for action performance that makes user nearer to the target behavior.
Tailoring	The system has to offer tailored information for its user group according to their interests, needs, personality, or other factors related to the user group.
Personalization	The system has to provide personalized content and customized services for users.
Self-monitoring	The system has to give means for users to track and monitor their performance, progress, or status in accomplishing their goals.
Simulation	The system needs to give means for observing and noticing the connection between the cause and effect of users' behavior.
Rehearsal	The system must deliver means for rehearsing a target behavior.
**Dialogue support**	
Praise	The system has to deliver praise through images, symbols, words, videos, or sounds as an approach to give user feedback information regarding his/her behavior.
Rewards	The system should offer virtual rewards for users to provide credit for doing the target behavior. The virtual rewards come in different forms such as collecting points or trophies, and changing media elements (e.g., background, sounds, or avatar), etc.
Reminders	The system has to remind users to perform their target behavior while using the system.
Suggestion	The system has to suggest ways that users can achieve the target behavior and maintain performing behavior during the use of the system.
Similarity	The system must imitate its users in some particular manner, so the system should remind the users of themselves in a meaningful way.
Liking	The system should be visually attractive and contain a look and feel that meets its users' desires and appealing.
Social role	The system has to adopt a social role by supporting the communication between users and the system's specialists.
**System credibility support**	
Trustworthiness	The system has to give truthful, fair, reasonable, and unbiased information.
Expertise	The system has to offer information displaying experience, knowledge, and competence.
Surface credibility	The system must have a competent look and feel that portrays system credibility based on an initial assessment.
Real-world feel	The system must give information of the organization and/or the real individuals behind its content and services.
Authority	The system should refer to people in the role of authority.
Third-party endorsements	The system should deliver endorsements from well-known and respected sources.
Verifiability	The system has to give means to investigate the accuracy of the system content through external sources.
**Social support**	
Social learning	The system has to give a user the ability to observe other users and their performance outcomes while they are doing their target behavior.
Social comparison	The system should enable users to compare their performance with other users' performance.
Normative influence	The system has to have a feature for gathering together individuals that have identical objectives and let them feel norms.
Social facilitation	The system should enable a user to discern other users who are performing the target behavior along with him/her.
Cooperation	The system should offer the opportunity for a user to cooperate with other users to achieve the target behavior goal.
Competition	The system should allow a user to compete with other users. In the competition principle, there is a chance for winning or losing a race.
Recognition	The system has to offer public recognition (e.g., ranking) for users who do their target behavior.

## 2. Literature Review

PT is a computer system that is designed to be interactive in a way that it can influence the attitude, beliefs, and behavior of the user to achieve a certain objective [4]. Fogg [5] further defined persuasive technology as “*the computing systems, devices, or applications intentionally designed to change a person's attitudes or behavior in a predetermined way*.” The use of the term “persuasion” implies that the attitude and behavior of the user can be changed in a predetermined way in accordance with the plans and design intents of the persuasive technology's designer. Within the health domain, PTs can be used to either promote health and prevent disease, or to manage diseases and health conditions [6]. Many researchers have designed PT to help people to change their lifestyle and become more active. We present an overview of the literature review of PT interventions targeting both the SB and PA health domain.

### 2.1. Sedentary Behavior

There are many studies that have examined and evaluated the effectiveness of digital interventions in the health domain that aim to reduce SB for individuals.

The majority of the PT studies in the SB domain have targeted office workers and workplace interventions. For example, Wang et al. [7] conducted a systematic review to evaluate the use and effectiveness of PTs targeting SB in the work environment using the PSD model. They found that reminders were the most employed strategy to reduce SB. However, reminders alone have no substantial impact on SB reduction.

Similarly, Gardner et al. [8] reviewed 26 studies and identified the behavior change strategies employed in the SB interventions using behavior change techniques (BCTs). They examined the effectiveness of the identified strategies. Their findings revealed that problem-solving, self-monitoring, and reorganization of the social or physical environment were effective strategies in decreasing SB among adults.

There are other workplace interventions that are aimed at reducing SB. For example, Healy et al. [9] presented a review of 11 studies that aimed to reduce SB and offer a healthy work environment. They reinforced the implementation of motivational strategies (e.g., the use of a combination of several strategies, the increase in the number of breaks taken from sitting time, the focus on comfortable changes to people's workplace, the change to a healthy posture periodically, etc.) to decrease prolonged workplace sitting and mitigate the risks of such unhealthy behaviors. These strategies played an essential role in improving the individual health status in the workplace environment, increasing productivity, and decreasing absenteeism and injury costs.

Similarly, Shrestha et al. [10] reviewed a total of eight studies that aimed to reduce SB in the workplace. A total of 1,125 users who participated in the study were divided into intervention groups: policy changes, physical workplace changes, and information and counseling. The findings indicated that sit-stand desks were able to decrease sitting time at work, while the consequences of the information and counseling as well as policy changes were unpredictable. All eight selected review studies provided low-quality evidence due to the high risk of bias, poor research design, and small sample sizes.

Moreover, Chu et al. [11] showed evidence in their review paper for intervention effectiveness in decreasing SB in the workplace environment, especially for multi-component interventions (e.g., the installation of sit-stand workstations with the use of wearable activity trackers in combination with behavioral change strategies), and environmental strategies (e.g., the use of sit-stand workstations, treadmill desks, stationary cycle ergometers, and portable elliptical/pedal machines). They showed that the use of multi-component interventions was more promising than implementing educational/behavioral strategies alone. However, they did not compare the effectiveness of different behavior change techniques “strategies,” as it is crucial to provide instructions and recommendations for PT design.

Addtionally, there are some studies that have evaluated the effectiveness of mobile applications in mitigating SB. For example, Dunn et al. [12] conducted a systematic review of persuasive strategies in 50 mobile applications (36 free apps, 14 paid apps) for reducing SB (e.g., sitting, laying on a bed, etc.) to identify the persuasive strategies employed in them using a taxonomy of 93 BCTs. The results showed that SB apps employed fewer persuasive strategies compared to PA mobile apps and other technology interventions in the health domains.

### 2.2. Physical Activity

Considerable studies have been focused in the area of analyzing the efficacy of PTs for promoting PA. Most of the PA interventions were mainly focused on using mobile applications and wearable devices technologies. McCallum et al. [13] examined 111 studies to evaluate PA promoting smartphone apps and wearable devices from different aspects: effectiveness, acceptability, engagement, and the implementation of rapid research designs. The results suggest the need to provide guidance to health and human-computer interaction (HCI) researchers in using more in-device sensors, user-logs, and rapid research designs.

Rao [14] provided a review paper on the usage of wearable activity monitoring devices for tracking and measuring PA in older people. Rao suggested that wearable sensors are perfect for measuring PA intensity, step counts, and energy expenditure, however; there is a need to enhance the accuracy of measurement in this type of PA, non-ambulatory PA, and the spatial extent of PA.

There were other mobile applications and wearable tracker device-based interventions that targeted increasing PA. Stephens and Allen [15], in their systematic review, examined user satisfaction and the usefulness of smartphone applications and text messaging technology to support PA and weight loss. Seven articles published between 2005 and 2010 were included in their review paper. Their results indicated that all the technology interventions that included educational support or had more interventions showed greater effectiveness for smartphone and text messaging for weight loss and the increase of PA.

Similarly, a review of Lau et al. [16] assessed the success and quality of methods used in the information and communication technologies (ICTs)-based PA domain (e.g., Internet and mobile phones), specifically for children and adolescent populations. Nine studies (published between 2001 and 2009) were included and analyzed in their review article. These studies provided PA related to behavioral, psychosocial, and cognitive outcomes. Their findings showed the positive effects of ICTs in the PA domain for children and adolescents, especially when implemented with additional delivery methods (e.g., the face-to-face approach).

Tong and Laranjo [17] also wrote a review paper that characterized and assessed the effects of social features integration in mobile health (mHealth) interventions in promoting PA. They included 19 studies in their research, and their findings showed that social aspects were mostly employed to offer social support or comparison. Furthermore, some individuals were more motivated by social support and social competition, while others had concerns about social comparison. They found that social features may increase user engagement and increase users' PA levels; however, they also found it too difficult to determine the most effective features for increasing PA in mobile health technology due to the multi-component interventions of most of the studies they reviewed.

Hardeman et al. [18] conducted a systematic review of just-in-time adaptive interventions (JITAIs) in mobile health (mHealth) technology for PA to determine these interventions' effectiveness, feasibility, features, and acceptability. There were 19 papers included in the review, and 14 unique JITAIs were identified. Hardeman and colleagues emphasized that research into JITAIs' effectiveness in decreasing SB and increasing PA in its early stages, and there is a need for more evidence by endorsing the robust assessment of theory and evidence-based JITAIs.

Ehn et al. [19] provided a qualitative study of “elderly” users' experiences of using activity monitors to track and measure their performance for supporting PA in daily life. There were eight users involved in the qualitative study, and they perceived the wearable devices as easy to handle. Ehn and partners suggested that activity monitors can be used for motivating elderly people to adopt a good level of PA and to promote a healthy lifestyle. However, Ehn et al. identified areas that need development and enhancement such as usability, reliability, and content supporting successful BCTs to increase older people's engagements in PA.

Hamasaki [20] summarized studies (published between 2015 and 2018) to investigate the efficacy of using wearable devices, particularly mobile applications, to manage diabetes for diabetic patients. A total of four studies were included in the review paper. Hamasaki's review results showed that the use of accelerometers or pedometers increased PA by about 1 h weekly, while diabetes and obesity rates were not changed. He also found that smartphone applications are beneficial for encouraging PA and treating diabetes. Consequently, the use of wearable devices and smartphone apps by diabetic patients increases their interactions due to the self-monitoring, education, and coaching features implemented in these technologies. However, the author mentioned that there is still a need to investigate the most useful wearable devices that can be used by diabetics patients to track their PA level, heart rate, blood glucose level, blood pressure, and energy balance accurately and comfortably.

Bort-Roig et al. [21] introduced a systematic review paper of smartphones app for PA with a total of 26 articles published between 2007 and 2013. They showed proof on smartphones and their ability to measure and influencing PA. Moreover, they recommended working on identifying and having well-designed studies to help in evaluating the accuracy of PA measurements along with employing long-term assessments.

Matthews et al. [22] provided a systematic review of 20 articles for health behavioral-change of mobile apps, especially those apps aimed at promoting PA. The authors employed the PSD model for evaluating the inbuilt persuasive strategies of mobile apps in their reviewed articles. Their findings showed that the most commonly employed persuasive strategies were primary task support, social support, and dialogue support, while the least frequently employed was credibility support.

Ghanvatkar et al. [23] offered a scoping review of 48 studies to address the use of a personalization strategy for PA interventions, to recognize the different types of personalization, and to identify the user models employed for delivering personalization. Their review covered only the studies that implemented a personalization strategy in the design of the PT for PA regardless of the use of other persuasive strategies. The authors provided some recommendations and feedback for the researchers and developers of PTs (e.g., fitness devices, mobile apps) in the use of personalization strategies to increase PA.

Other studies have evaluated different PT interventions in encouraging PA. For instance, Almutari and Orji [24] presented an empirical review of 19 years (54 studies) of literature on PT for influencing PA. The authors included 54 papers (published from 2000 to 2019) in their report to assess the effectiveness of implementing social support strategies in PT for PA. They only included papers that focused mainly on employing the most frequently used social support strategies as social cooperation, social comparison, and social competition. Their findings suggest that PTs implementing socially-oriented strategies in the design of PT are considered successful tools to encourage and increase users' PA levels. The review papers conducted by Win et al. [25, 26] are other examples of a PT intervention in PA.

### 2.3. Studies Examining Both Physical Activity and Sedentary Behavior

This section includes the review papers that have focused on PT interventions in the area of both increasing PA and reducing SB.

A number of studies combined both PA and SB. For example, Prince et al. [27] provided a qualitative analysis of systematic review papers, including six studies in the PA and/or SB health domain. The authors aimed to provide a comparison of the efficacy of the interventions used on PA and/or SB to decrease the time spent sedentary in the adult population. Their findings indicate that a huge and clinically significant decrease in sedentary time can be achieved using interventions concentrating on reducing SB.

Schembre et al. [28] in their systematic review, evaluated data on the content features of feedback messaging employed in diet, PA, and SB interventions. The authors also created a practical framework to help developers to design just-in-time feedback for health behavior change in individuals. Approximately 31 studies were included in their review, in which 30 used personalized feedback, 24 employed goal-oriented feedback, and just 5 implemented actionable feedback. Furthermore, their results show that the feedback was often available, personalized, and actionable feedback with substantial behavior change outcomes.

Schoeppe et al. [29] investigated the effectiveness of health interventions that employ smartphone apps to enhance PA, SB, and diet in children and adult populations. Their systematic review examined twenty-seven studies published between 2006 and 2016. The results suggested that app-based interventions can be very useful in improving diet, PA, and SB. Furthermore, multi-component interventions seemed to be more promising and effective than stand-alone smartphone app interventions.

Yim and Graham [30] reviewed the literature on PA motivation and SB reduction by investigating the properties of digital exercise games. The authors introduced an exercise game called “Life is a Village” to demonstrate the exercise motivation needs and requirements for computer-aided exercise games.

The objective of the review paper by Lister et al. [31] was to identify and analyze the use of gamification in health and PA “fitness” apps in motivating users to adopt desirable and healthy behavior. Lister and partners examined health apps from the Apple App Store that were associated with diet and PA domains. The authors reviewed 132 apps and determined the top ten successful game elements, the top six essential health gamification elements, and the 13 most fundamental health behavior concepts. Their results indicated that the use of gamification in fitness and health apps was prevalent, and there was a lack of implementing behavior theory elements in the app industry.

It is obvious from the above literature review of the related work that some systematic studies have focused only on one specific domain, either PA or SB. Others have considered both fields of reducing SB and increasing PA while focusing on targeting a particular technology, population, or strategy. However, none of these studies have provided a comprehensive overview of the development and trends of PTs in PA and/or SB domains. For example, some reviews concentrated on a particular PT such as the use of smartphone apps, wearable devices, or games in promoting physical activity. Other papers focused only on reviewing studies that used one or a particular set of motivational strategies such as personalization or social support features, whereas another collection of papers focused on a specific target audience, such as children, elderly, or adults. Therefore, there is a need to provide a systematic review paper that offers a comprehensive overview of PTs in both PA and SB domain to bridge existing gaps from the review papers.

## 3. Materials and Methods

The aim of this study is to evaluate the effectiveness of PT in reducing sedentary lifestyles and increasing the level of PA. The research questions of our systematic review paper are:

- To what extent are PTs effective in promoting PA and reducing SB?
- What are the outcomes' trends of employing PTs in promoting PA and reducing SB?
- What persuasive strategies were employed in designing PTs for PA and SB and how were they implemented?
- What are the pitfalls and gaps in the present literature on PT for PA and SB?
- What are the opportunities and recommendations for future PTs design?

We conducted a systematic review of 170 published papers in the PA and SB domains between 2003 and 2019. To achieve this, we used quantitative content analysis, a technique that enables the comparison, contrast, and categorization of data according to different themes and concepts, as adapted from Orji and Moffatt [4]. This entails collecting data in a rigorous way, paying special attention to the objectivity of the results. To retrieve articles for this review, we searched various databases including Springer, PubMed, ACM Digital Library, EBSCOHost, ProQuest, Google Scholar, Elsevier Scopus, and IEEE Xplore. The databases were selected to ensure that articles across various fields would be accessed for the study.

As shown in Table 2A, various keywords were used in the search process such as “Physical Activity,” “Physical Activity Applications or Apps,” “Sedentary Behavior or Behaviour,” “Sedentary Behavior or Behaviour Applications or Apps,” “Sedentary Lifestyle,” “Prolonged Sedentary,” “Prolonged Sedentary Behavior,” “Prolonged Sedentary Sitting,” “Prolonged Sitting,” “Physical Activity and Sedentary Behavior,” “Persuasive Technology and Physical Activity,” “Persuasive Technology and Sedentary Behavior,” “Persuasive Technology and Physical Activity and Sedentary Behavior,” “Persuasive Technology Exercise,” “Persuasive Technology Fitness,” “Physical Activity and Gamification,” “Physical Activity and Exergames,” “Exercise Applications Or Apps,” “Fitness Applications or Apps,” “Exergames or Mobile Exergames.” The search was refined through the use of Boolean terms such as “Persuasive Technology AND Physical Activity AND Sedentary Behavior.” We adapted Table 2A from the previous work done by Wang et al. [7] and refined using more keywords identified from the literature, in the refine process.

**Table 2:** **(A)** Search terms and combination methodology for articles selections; **(B)** Persuasive technology classifications and coding scheme analysis—Adapted from Orji and Moffatt [4].

**(A)**				
**Search terms method**				
**Numbers**	**Terms**	**Combinations**		**Search terms**
1	Physical	1 and 2	1 and 2 and 7	° Physical Activity ° Physical Activity applications or apps ° Sedentary Behavior or Behavior ° Sedentary Behavior or Behavior applications or apps ° Sedentary Lifestyle ° Prolonged sedentary ° Prolonged sedentary behavior ° Prolonged sedentary sitting ° Prolonged sitting ° Physical activity and sedentary behavior ° Persuasive Technology and Physical activity ° Persuasive Technology and sedentary behavior ° Persuasive Technology and Physical activity and sedentary behavior ° Persuasive Technology Exercise ° Persuasive Technology Fitness ° Physical activity and Gamification ° Physical activity and Exergames ° Exercise applications or apps ° Fitness applications or apps ° Exergames or Mobile exergames ° Fitness Technology ° Exergame Technology ° Fitness ° Exergames
2	Activity	1 and 2 and 3 and 6	1 and 2 and 3 and 6 and 4 and 5	
3	Sedentary	1 and 2 and 11	1 and 2 and 12	
4	Persuasive	1 and 2 and 13	1 and 2 and 14	
5	Technology	1 and 14	1 and 15	
6	Behavior or behavior	1 and 2 and 15	3 and 6	
7	Applications or apps	3 and 6 and 7	9 and 3	
8	Lifestyle	9 and 3 and 6	9 and 3 and 7	
9	Prolonged	9 and 3 and 10	9 and 10	
10	Sitting	4 and 5 and 1 and 2	4 and 5 and 3 and 6	
11	Exercise	4 and 5 and 1 and 2 and 3 and 6	4 and 5 and 11	
12	Fitness	4 and 5 and 12	15	
13	Gamification	16 and 15	11 and 7	
14	Games	16 and 1 and 2 and 14	12 and 7	
15	Exergames	3 and 8	12 and 5	
16	Mobile	12	15 and 5	

**Table:** 

**(B)**		
**PT classifications and coding scheme**		
**S/N**	**Identification**	**Examples/meaning**
1	Papers	Name of the research papers and articles.
2	Author(s)	Name of the author(s) who wrote a research paper and conducted a study.
3	Year	The year of when the study was conducted.
4	Domain Focus	PA, SB, Eating, Smoking, Stress, Obesity, Sitting Postures, Mental Health, etc.
5	Technology	Mobile, Web, Games, Computer applications, Ambient displays, etc.
6	Evaluation Methods	Quantitative, Qualitative, and Mixed.
7	Persuasive Strategies	Motivational affordance strategies used in a PT system design.
8	Duration of Evaluation	Minutes, Hours, Days, Weeks, Months, and Years.
9	Behavior Theories	Theories used in a PT system design or assessment.
10	Targeted Outcomes	Behavior, Attitudes, Awareness, Adherence, Motivation, Feasibility, Cognitive, etc.
11	Targeted Audience based on their Age Group	Children, Teenagers, Young Adults, Adults, Elderly, etc.
12	Targeted Audience based on their Status/Occupations/Health Conditions	School students, University students, Office workers, Overweight and Obese, Nurses, Patients with type 2 diabetes, etc.
13	Number of Participants	Number of participants who participate in the assessment of a study.
14	Venue	CHI, UbiComp, Persuasive, MobileHCI, Pervasive Health, Health Informatics, JMIR, etc.
15	Effectiveness/Evaluation Outcomes	Identifying whether the study was successful or not successful.
16	Country/Region of a Study	Country or region where the study was conducted.

The search in the databases was also refined using an inclusion and exclusion criteria. The first criteria was to include recent articles, so those articles published earlier than the year 2003 were excluded from the search because the first paper in the field of persuasive technology was introduced by Fogg [32] as a seminar paper in the year of 2002. Accordingly, most papers in the area of PT where published from the year of 2003. This was also to ensure that the findings reported in the studies were current and not outdated. The second criteria was that only articles that were in English were selected for the study. The search was run through the databases to locate relevant articles. The reference lists of these articles were also reviewed to further identify other potentially relevant articles.

### 3.1. Analysis and Coding Scheme

**Figure 1:**
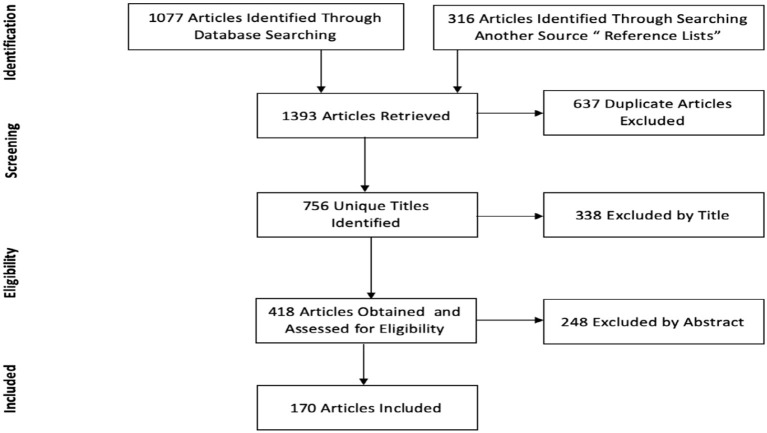
PRISMA flow diagram for the study selection workflow.

We retrieved 1,393 articles, of which 1,077 articles were identified through database searching, and 316 articles were identified through reviewing the reference lists of the obtained articles. There were 637 duplicate articles excluded from the total of 1,393 articles. The titles of these articles were examined, and those found not to be suitable were excluded, such as those that targeted health domains other than PA/SB. Overall, we identified 756 unique titles, of which 338 articles were excluded by titles, and after evaluating the abstracts of the remaining 418 articles, 170 articles were selected for final analysis. The study identification process is summarized in Figure 1 [a PRISMA flow diagram [33]].

In the second step of this review, we coded the articles by creating an excel coding sheet for the PT analysis. As a starting point, we adopted a coding sheet that was developed and validated by Orji and Moffatt [4] and refined it by adding new coding categories that emerged as we iteratively analyzed our data. Table 2B shows how we classified and coded the articles. Once the articles were identified, they were coded and classified, as shown in Appendix 1.

## 4. Results

The analysis of PTs for physical activity and SB reveal some interesting findings, as shown below. The findings are presented under various categories such as the year and country in which the technology was developed, the platforms, behavioral and psychological outcomes targeted, and the evaluation results of the PTs. The summaries of all the reviewed papers are as shown in Appendix 1. For the papers that have more than one study, we combined the findings for all the studies in the paper. For example, we reported the total number of participants, all the persuasive strategies used, and the total duration of all the studies in each paper.

### 4.1. Persuasive Technology for Physical Activity and Sedentary Behavior by Year and Country

**Figure 2:**
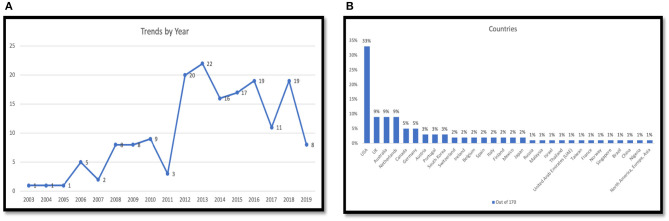
**(A)** Persuasive technology for physical activity and sedentary behavior trend by year; **(B)** Persuasive technology for physical activity and sedentary behavior by study country/region.

As shown in Table 3A and Figure 2A, a large number of articles and studies were published after compared to before 2011. In recent years, there has been a sharp increase in the number of articles published since 2012, although the number fluctuated year to year from 2012 to 2019. It is important to mention that while 2019 appears to have the lowest number of studies since 2012, this is probably because most papers for 2019 are yet to be published at the time of this study, third quarter of 2019.

**Table 3:** **(A)** Persuasive technology for physical activity and sedentary behavior trends by year; **(B)** Persuasive technology for physical activity and sedentary behavior by study country/region.

**(A)**			
**Counrty**	**Study**	**Total**	**Overall of % 170**
2003	[34]	1	1%
2004	[35]	1	1%
2005	[36]	1	1%
2006	[37–41]	5	3%
2007	[42, 43]	2	1%
2008	[44–51]	8	5%
2009	[52–59]	8	5%
2010	[60–68]	9	5%
2011	[69–71]	3	2%
2012	[72–91]	20	12%
2013	[92–113]	22	13%
2014	[114–131]	16	9%
2015	[130–146]	17	10%
2016	[147–165]	19	11%
2017	[166–176]	11	6%
2018	([177–194]; [195])	19	11%
2019	[196–203]	8	5%
**(B)**			
**Counrty**	**Study**	**Total**	**Overall of % 170**
USA	[34, 35, 39, 41, 44, 46, 50, 51, 54, 59, 60, 64, 68, 69, 74, 80, 84, 85, 87, 89, 90, 93, 96, 99, 100, 102, 105, 106, 114, 117, 123, 125, 127, 128, 131, 134, 136, 140, 148, 150, 156, 158, 162, 164–167, 173, 184, 188–191, 199, 200, 203]	56	33%
Australia	[61, 73, 75, 92, 94, 96, 98, 147, 151, 152, 174, 201] [58, 109, 126]	15	9%
Austria	[49, 81, 88, 111, 141]	5	3%
Portugal	[120, 135, 139, 159, 186]	5	3%
Canada	[67, 72, 77, 83, 96, 122, 185, 187, 199]	9	5%
UK	[40, 42, 63, 78, 79, 82, 101, 104, 118, 124, 142, 154, 161, 168, 171, 196]	16	9%
Russia	[169]	1	1%
Malaysia	[157]	1	1%
Israel	[129]	1	1%
Thailand	[137]	1	1%
Switzerland	[38, 115, 130, 198]	4	2%
Germany	([62, 95, 170, 176–178, 192, 193]; [202])	9	5%
Netherlands	[37, 52, 53, 57, 66, 70, 71, 91, 110, 113, 145, 160, 175, 181, 195]	15	9%
United Arab Emirates (UAE)	[103]	1	1%
Taiwan	[116]	1	1%
Italy	[76, 119, 132]	3	2%
Finland	[55, 133, 153]	3	2%
Mexico	[48, 65, 112]	3	2%
South Korea	[43, 86, 108, 138, 155]	5	3%
Ireland	[34, 96, 97, 163]	4	2%
Belgium	[143, 144, 149, 197]	4	2%
France	[183]	1	1%
Norway	[182]	1	1%
Singapore	[56]	1	1%
Brazil	[45]	1	1%
China	[172]	1	1%
Japan	[36, 47, 107]	3	2%
Nigeria	[199]	1	1%
Spain	[146, 179, 180, 194]	4	2%
North America, Europe, Asia	[121]	1	1%

As it is evident from Table 3B and Figure 2B, the studies were conducted in 29 different countries, with most of the studies coming from the USA, 56 (33%). This is followed by the UK with a total of 16 articles. Australia and the Netherlands are in third place, with a total of 15 articles for each. Canada and Germany are in fourth with a total of 9 articles. Only one article did not specify the country where the study was conducted, only mentioning the continent such as North America, Europe, and Asia.

### 4.2. Effectiveness of Persuasive Technology for PA and SB

**Figure 3:**
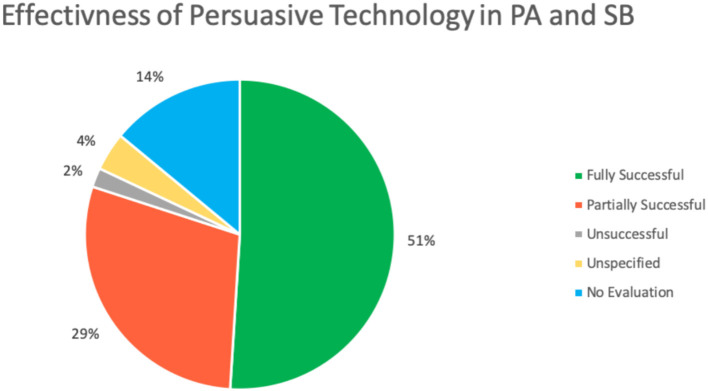
Effectiveness of persuasive technology in physical activity and sedentary behavior.

Table 4 and Figure 3 show a summary of the results of the effectiveness of PT for PA and SB reviewed in this paper. We found that 87 (51%) studies reported fully successful outcomes, and 50 (29%) studies reported partially successful outcomes from using the PT to achieve desired behaviors and attitudes related to PA and/or SB. Partially positive results are used to describe studies that reported a combination of positive with negative or no effect results [4]. However, only 4 (2%) of the studies reported completely unsuccessful results. In the studies reviewed, 6 (4%) did not specify the outcomes of the technology, and 23 (14%) of the articles did not evaluate their PT design. As a result, most of the reviewed studies (80%) reported successful outcomes, whether fully or partially, while only 4% of the studies were unsuccessful. This means that PTs are effective tools to persuade people in practicing more PA and reducing their SB.

**Table 4:** Summary results of Persuasive Technology (PT) Effectiveness in Physical Activity (PA) and Sedentary Behavior (SB).

**Results**	**Study**	**Total**	**Overall of % 170**
Successful	[34, 35, 37–39, 41, 44–50, 54, 56–58, 60–64, 66–70, 72, 73, 75, 76, 79, 80, 83, 85, 86, 89, 92–95, 100, 101, 103, 109, 110, 114–118, 121, 122, 124, 126, 128, 130, 131, 136, 137, 139, 141, 144, 146–151, 153–156, 158, 163, 166, 169, 173–176, 184, 188, 191, 196, 197, 203]	87	51%
Partially successful	([36, 42, 43, 51–53, 59, 71, 77, 81, 84, 96, 99, 104, 105, 112, 113, 120, 123, 125, 129, 132–135, 140, 142, 143, 160, 164, 167, 170, 172, 177–183, 185–187, 190, 192, 193]; [195, 199, 201, 202])	50	29%
Unsuccessful	[91, 107, 108, 165]	4	2%
Unspecified	[40, 55, 97, 98, 138, 152]	6	4%
No evaluation	[65, 74, 78, 82, 87, 88, 90, 102, 106, 111, 119, 127, 145, 150, 159, 161, 162, 168, 171, 189, 194, 198, 200]	23	14%

### 4.3. Major Technology Platforms Employed in PTs for Physical Activity and Sedentary Behavior and the Effectiveness of PTs

**Figure 4:**
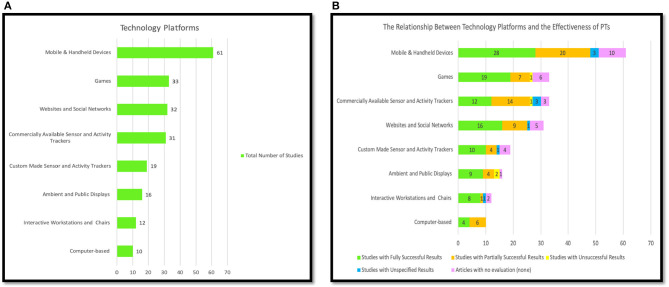
**(A)** Persuasive technology platforms. **(B)** Technology platforms and the effectiveness of PTs.

Figure 4A provides a summary of the major technology platforms employed to design the PTs for PA and SB. Mobile and handheld devices were the most used platform with a total of 61 studies (36%), followed by platforms that employed games and gamifications with precisely 33 (19%) studies, as well as web and social networks that placed second with 32 studies (19%). The games category includes all the interventions that were delivered in the form of games, irrespective of whether the game is web-based, a mobile, or a desktop device. We found that 31 (18%) studies used commercially available sensors and other activity trackers (e.g., Fitbit, Pebble smartwatch, ActivPAL, and ActiGraph), whereas 19 (11%) used custom-designed sensors and activity trackers that have been designed by the researchers in their studies. Ambient and public displays came in fifth place with 16 (9%) studies using this platform, this was followed by the interactive workstations and chairs with just 12 (7%) studies. Computer-based platforms such as desktop and laptop were the least frequently employed platform for delivering PTs for physical activity and sedentary behavior, with only 10 (6%) studies.

It is important to mention that most of the reviewed studies employed more than one technology platform in their PT design. Generally, the second most employed technology platforms after the mobile and handheld devices are activity trackers and sensors (whether commercial or custom-designed) with a total of 42 studies (29%). Consequently, by considering the use of embedded sensors in mobile devices, we can notice that the dominant technology platforms employed in the PTs for PA and SB were activity trackers and sensors and most PT employing them were successful. Thus, it is essential to employ activity trackers and sensors in the PT design to track users' performance and to provide them with accurate feedback about their activity progress to motivate them to change their unhealthy habits such as SB.

Figure 4B demonstrates the effectiveness of employing PT with regards to the technology platforms. For the mobile and handheld devices, we found that 48 (79%) of the studies reported successful results; that is, studies with partially successful and those with fully successful results. Precisely, 28 (58%) studies were fully successful, and 20 (42%) studies were partially successful. For the games, out of 33 studies employing them, 19 (58%) showed fully successful outcomes, 7 (21%) displayed partially successful outcomes, just 1 (3%) reported unsuccessful outcomes, and 6 (18%) did not provide evaluations. For the commercially available sensors and activity trackers, out of 33 studies using them, 12 (36%) reported fully successful results, 14 (43%) showed partially successful results, only 1 (3%) reported unsuccessful outcomes, 3 (9%) reported unspecified results, and 3 (9%) did not evaluate their studies. For the websites and social networks, out of 31 studies implementing them, 16 (52%) reported fully successful results, 9 (29%) showed partially successful results, only 1 (3%) did not specify the results, and 5 (16%) did not evaluate their PTs. For the custom made sensors and activity trackers, out of 19 studies designed them, 10 (53%) reported fully successful results, 4 (21%) provided partially successful results, 4 (21%) did not show evaluations, and only 1 (5%) reported unspecified results. For the ambient and public displays, out of 16 studies employing them, 9 (56%) reported fully successful results, 4 (25%) showed partially successful results, 2 (13%) reported unsuccessful outcomes, and 1 (6%) did not evaluate their studies. For the interactive workstations and chairs, out of 12 studies implementing them, 8 (67%) reported fully successful results, 1 (8%) showed partially successful results, only 1 (8%) did not specify the results, and 2 (17%) did not evaluate their PTs. For the computer-based technology such as a desktop, we found that 10 of the studies reported successful results; that were 6 (60%) studies with partially successful, and 4 (40%) studies with fully successful results. Overall, the findings show that the most effective technology platforms are mobile and handheld devices with 48 successful studies (whether fully or partially successful), followed by activity trackers and sensors (whether commercial or custom-designed) with 40 successful studies, and then games with 26 successful studies, followed by websites and SNSs with 25 successful studies.

### 4.4. Persuasive Strategies and Motivational Affordances

**Figure 5:**
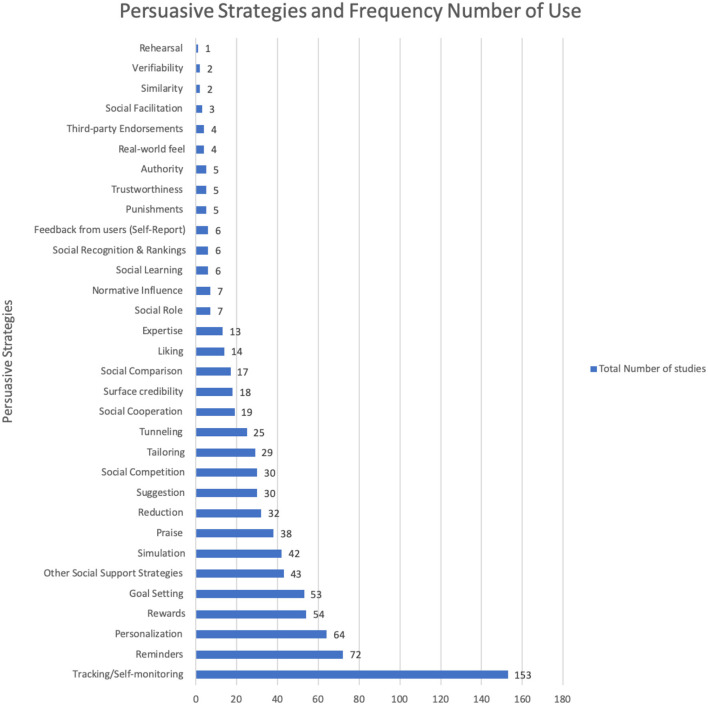
Persuasive strategies and frequency of use.

Table 5 and Figure 5 show the strategies most commonly employed to bring about the intended behavioral outcomes in the PA and/or SB domains. Tracking and self-monitoring were the most frequently employed strategies with a total of 153 (90%) studies. Reminder ranked as the second most employed strategy with 72 (42%) studies, and personalization is the third most employed strategy with a total of 64 (38%) studies. Rewards and goal-setting ranked as the fourth and fifth frequently employed strategies with 54 (32%) studies and 53 (31%) studies respectively using the strategy. Other social support strategies (which refer to those strategies that did not belong precisely to the PSD model or those that were not specified such as social comments, tags, likes, chatting, and sending invitations, etc.) came sixth, with a total of 43 (25%) studies implementing these strategies. Simulation came in seventh place with a total of 42 (25%) studies, and praise came eight, with a total of 38 (22%) studies. Thirty-two (19%) studies employed the reduction strategy, which was the ninth most frequently used strategy. Suggestion and social competition strategies emerged as the tenth and eleventh most frequently used strategies with 30 (18%) studies employing each of them. Finally, tailoring, tunneling, social cooperation, surface credibility, social comparison, liking, and expertise credibility emerged as the 12, 13, 14, 15, 16, 17, and 18th most frequently used strategies, respectively, with a total of 29 (17%), 25 (15%), 19 (11%), 18 (11%), 17 (10%), 14 (8%), and 13 (8%) studies, see Figure 5.

**Table 5:** Persuasive strategies for PT of physical activity and sedentary behavior.

**#**	**Motivational strategies/ Affordances**	**Studies with fully successful results**	**Studies with partially successful results**	**Studies with unsuccessful results**	**Studies with unspecified results**	**Articles with no evaluation (none)**	**Total number of studies**	**Average out of % 170 for each**
1	Reduction	[41, 45, 60, 61, 64, 67, 70, 76, 80, 86, 100, 116, 117, 139, 157, 166, 175, 191, 196]	[104, 123, 135, 140, 181, 182, 185]		[55]	[74, 106, 150, 159, 198]	32	19%
2	Tunneling	[37, 45, 64, 66, 86, 94, 100, 117, 122, 137, 139, 157, 173, 191, 197]	[52, 104, 123, 182, 192]			[102, 111, 119, 127, 189]	25	15%
3	Tailoring	[37, 50, 54, 73, 79, 85, 89, 98, 100, 126, 130, 146, 149, 157, 174, 175, 197]	[36, 81, 112, 123, 183, 185, 199]		[55, 98]	[88, 119, 168, 198]	29	17%
4	Personalization	[41, 60, 63, 67–70, 72, 73, 76, 83, 85, 86, 89, 98, 114, 124, 126, 130, 137, 141, 149, 153, 157, 158, 169, 174, 175, 191, 196, 197, 203]	[42, 43, 51, 71, 123, 167, 170, 178, 182, 183, 185–187, 190, 193, 195, 199, 202]	[91, 107]	[55, 97, 98]	[88, 90, 106, 127, 150, 159, 162, 168, 189, 200]	64	38%
5	Tracking/Self-monitoring	[34, 35, 37–39, 41, 44–48, 50, 57, 60–64, 67–70, 73, 75, 76, 79, 80, 85, 86, 89, 92–95, 100, 101, 103, 109, 114–116, 118, 121, 124, 126, 128, 130, 131, 136, 137, 139, 141, 144, 146, 148, 149, 151, 153–158, 163, 166, 169, 173, 175, 176, 184, 188, 191, 196, 197, 203]	([36, 42, 43, 51–53, 59, 77, 81, 84, 96, 99, 104, 105, 112, 113, 120, 123, 125, 129, 132–135, 140, 142, 143, 160, 167, 170, 172, 177–180, 182, 183, 185–187, 192, 193]; [195, 199, 201, 202])	[91, 107, 108, 165]	[40, 55, 97, 98, 138, 152],	[74, 78, 119, 150, 168, 198] [65, 82, 87, 90, 102, 106, 111, 127, 145, 159, 161, 162, 171, 189, 194, 200]	153	90%
6	Simulation	[34, 35, 39, 46, 56, 58, 64, 66, 67, 72, 73, 76, 83, 89, 98, 122, 124, 126, 130, 141, 148, 157, 174–176]	[36, 104, 120, 134, 160, 180, 181]	[91, 107]	[98, 138]	[82, 88, 106, 111, 119, 194, 198]	42	25%
7	Rehearsal	[157]					1	1%
8	Praise	[37, 41, 44, 46, 48, 50, 64, 68, 85, 92, 100, 114, 146, 157, 191, 196, 197]	[51, 81, 112, 113, 129, 134, 135, 177, 179, 183, 192, 193, 195]	[91]	[55]	[74, 87, 90, 106, 119, 189]	38	22%
9	Rewards	[39, 44, 49, 60, 61, 64, 66, 68, 69, 75, 76, 80, 85, 110, 114–116, 121, 122, 124, 137, 141, 153, 191, 196]	([43, 52, 81, 84, 104, 112, 129, 135, 167, 177, 178, 181, 182, 185, 192, 193]; [199])	[91, 107]	[55]	[65, 74, 82, 102, 106, 145, 161, 171, 189]	54	32%
10	Punishments	[39, 49]	[43]	[107]		[119]	5	3%
11	Reminders	[35, 38, 44, 50, 54, 66, 69, 70, 79, 85, 89, 92, 93, 95, 114, 126, 130, 136, 141, 144, 151, 153–155, 157, 158, 169, 175, 176, 191, 196, 203]	[43, 51, 53, 71, 81, 84, 96, 99, 112, 113, 120, 123, 133, 140, 160, 164, 167, 170, 178, 179, 183, 185–187, 190, 195, 202]	[107, 165]	[55, 138]	[74, 87, 102, 111, 145, 150, 171, 189, 194]	72	42%
12	Suggestion	[45, 47, 54, 66, 70, 85, 100, 117, 122, 126, 131, 136, 139, 146, 149, 157, 173, 175]	[81, 84, 96, 112, 120, 123, 135, 195]		[138]	[87, 150, 168]	30	18%
13	Similarity	[157]	[178]				2	1%
14	Liking	[72, 75, 124, 141, 175]	[36, 123, 181, 185, 193]	[91, 108]		[189, 198]	14	8%
15	Social role	[175, 191]	[187]		[55]	[106, 119, 159]	7	4%
16	Trustworthiness	[100, 191]	[81]		[55]	[168]	5	3 %
17	Expertise	[100, 141, 144, 149, 157, 191, 196, 197]	[53, 81, 187]		[55]	[168]	13	8%
18	Surface credibility	[100, 131, 144, 158, 175]	[84, 105, 132, 135, 202]	[91]	[55]	[74, 90, 102, 119, 168, 171]	18	11%
19	Real-world feel	[64, 100, 157, 196]					4	2%
20	Authority	[100, 191]	[71]			[150, 168]	5	3%
21	Third-party endorsements	[100, 157]				[168, 171]	4	2%
22	Verifiability	[100, 157]					2	1%
23	Social learning	[38, 69, 157]	[172, 199]			[168, 200],	7	4%
24	Social comparison	[157, 175, 203]	([42, 51, 113, 129, 143, 177–179]; [199])	[91]		[74, 82, 119, 200]	17	10%
25	Normative influence	[100, 157, 191]	([113, 177, 178])			[168]	7	4%
26	Social facilitation	[38, 157]	[81]				3	2%
27	Social cooperation	[39, 41, 47, 48, 68, 86, 115, 184, 191]	[43, 81, 172, 177, 180, 192, 199]			[65, 74, 119, 145, 162, 198]	19	11%
28	Social competition	[34, 39, 41, 45–47, 56, 58, 60, 66, 68, 86, 94, 103, 115, 116, 153, 157, 184]	[42, 43, 51, 134, 177, 181, 182]	[91]		[74, 145, 162]	30	18%
29	Social recognition & rankings	[153, 157]	[172]	[91]		[145, 168]	6	4%
30	Other social support strategies	[38, 39, 41, 47, 48, 58, 64, 66, 68, 69, 85, 86, 100, 103, 115, 121, 124, 137, 146, 188, 191, 197, 203]	[42, 51, 53, 84, 112, 113, 143, 160, 172, 179]	[91, 108]	[97]	[65, 74, 90, 145, 150, 168, 171]	43	25%
31	Goal setting	[38, 39, 44, 47, 48, 63, 64, 68, 85, 92, 100, 114, 121, 126, 139, 146, 175, 184, 188, 191, 196, 197]	[43, 51–53, 81, 84, 99, 104, 112, 113, 123, 129, 135, 143, 179, 181–183, 185–187, 199]		[55, 97]	[74, 82, 106, 168, 171, 189, 200]	53	31%
32	Feedback from users (Self-Report)	[50, 68, 70, 158]	[190]			[90]	6	4%

### 4.5. Examples of Persuasive Strategies Employed in the Reviewed Studies

It is important to note that most of the studies employed more than one strategy at a time, and each strategy may have been implemented differently from one study to another. For example, the main strategy used by a study could be self-monitoring, but the app may also provide feedback that may appear in different formats such as audio, visual or textual feedback. It is essential to mention that we relied mainly on the PSD model [3] in sorting and organizing the persuasive strategies we obtained from the reviewed articles. Table 1 summarizes the PSD model principles' “strategies.” However, we identified some strategies that were not capured in the PSD model such as goal setting, punishments, self-report, and other social support strategies. For instance, goal setting is not part of the strategies highlighted in the PSD model; however, it is clearly an example of the persuasive strategies that have been employed in many PA and SB applications.

Other social support strategies (which refer to strategies that did not belong precisely to the PSD model or those that were not specified) such as (social sharing, social set/accept challenges, social posting feeds, social sending likes, social follow, social messages exchange (e.g., sending encouraging feedback, invitation, chatting), social interaction (e.g., communicating via video-conferencing “video streams, microphone”), social giving comments, and tagging).

#### 4.5.1. Punishment Strategy

The punishment strategy also known as “negative reinforcement” does not belong to any strategy in the PSD model. An example of a punishment strategy was a sad or an angry emotional facial expression of fish in a social computer game called “Fish ‘n’ Steps” [39], and a negative expression, such as in the “Persuasive Art” ambient mirror system [107]. It's also exemplified in apps where users lose some points for not meeting their goals.

#### 4.5.2. Tracking/Self-Monitoring Strategy

**Figure 6:**
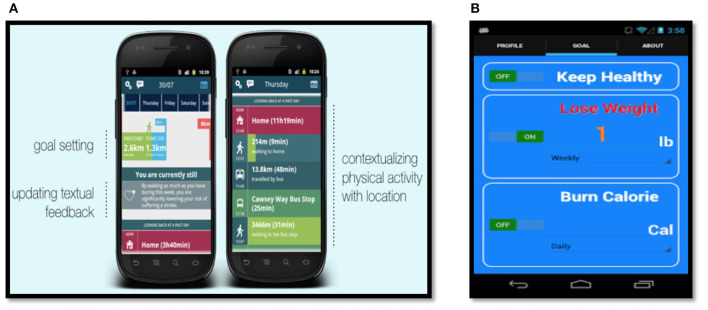
**(A)** Visual and textual feedback in habito mobile activity tracker system [135]; **(B)** Visual and textual feedback in on11 mobile system [123].

The examples of tracking/self-monitoring strategy were diverse in the reviewed papers. For example, tracking/self-monitoring could be in the form of textual and visual feedback of a user's progress, step counts, approximate burnt calories, and goal completion as can be seen from Figure 6 of the mobile activity tracker system called “Habito” [135] and the “On11” mobile system [123]. Real-time and vibration feedback were used as tracking/self-monitoring in the mobile game called “LocoSnake” because the phone vibrates when the snake's head goes near a piece of fruit [76]. Tracking/self-monitoring also was represented as a graphical and informative art visualization such as in the “Spark” web application [80]. Another tracking/self-monitoring strategy was the “Pediluma” show activity tracker device that monitors the wearer's PA by providing various light intensity levels regarding the user's status based on whether he/she was engaged in PA (e.g., walking) or sedentary [69]. The sculpture in the “Breakaway” ambient display system was used as a tracking/self-monitoring strategy and a reminder strategy since there was a connection between the user's movements (whether sedentary or physically active) and the sculpture placed on the office workers' desks [35].

#### 4.5.3. Authority Strategy

An authority strategy was implemented as an example, as was presented in the “PRO-fit” system by using the OAuth 2.0 protocol [150]. The Calendar Integration Manager (CIM) module allows the “PRO-fit” system to integrate many calendar services providers such as Yahoo, Hotmail, Google, etc. [150].

#### 4.5.4. Third-Party Strategy

A third-party endorsement strategy was used in the “WragaFit” application, as the PA goals were set by the Ministry of Health [157]. Another example was represented in the “WeightBit” application, which used the Apple Technology Company's Health Kit [171].

#### 4.5.5. Simulation Strategy

The simulation strategy was found in PTs such as the mobile game called “LocoSnake,” in which the user represents a virtual snake in the game. When the user walks and moves in the real world, this controls the movement of the snake with the help of GPS and visualized satellite map technologies [76]. Another example was the interactive “GrabApple” game, which requires a player to make movements in the physical world such as raising their hands and jumping to pick up virtual falling green and red apples in the game on the screen [122]. Another instance was the “Energy Browser” system which allows users to wear activity sensor devices, and to observe the effects of their healthy physical movements while walking or running on treadmills [36]. Another example of a simulation strategy was in a web and smartphone game called “Phone Row” in which the users control the movements of a virtual boat through a virtual route on an outer screen [91]. The previously mentioned examples of a simulation strategy gave the user the ability to observe the connection between the cause and effect regarding his/her behavior, which reflects the definition of the simulation strategy in the PSD model [3].

#### 4.5.6. Suggestion Strategy

The suggestion strategy or what is known as persuasive messages or recommendations were shown in the following example (e.g., “Try walking when talking on the phone. During your call with Bob, you were sedentary,” “Last week, you reached your daily walking goal two times, try updating it to 8 km”) [135].

#### 4.5.7. Goal Setting Strategy

**Figure 7:**
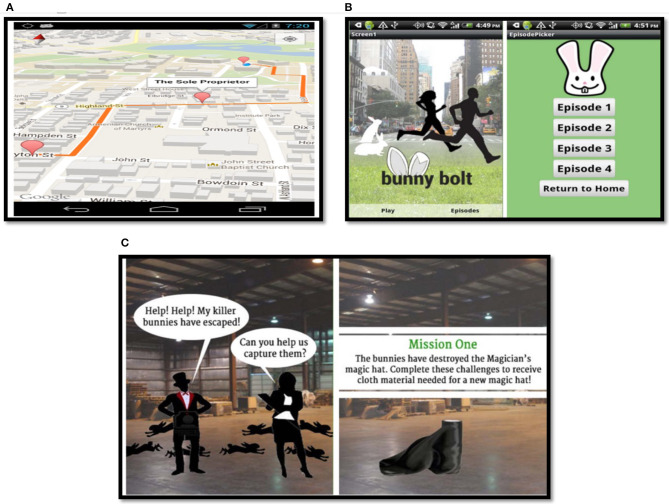
**(A)** On11 detour map [123]; **(B)** BunnyBolt game [102]; **(C)** BunnyBolt game scenario [102].

A goal setting strategy was used in the smartphone application called “On11,” which allows users to set their performance goal to enable the system to recommend the users suitable activities based on their conditions (time, weather, location) to assist them to meet their goals [123], as shown in Figure 7A.

#### 4.5.8. Tunneling Strategy

An example of a tunneling strategy was found in the “On11” system by generating walking routes to guide users through the use of Google Directions API [123]. The smartphone-based exergames called “Go Run Go” [137], and “BunnyBolt” [102] as shown in Figures 7B,C, represented a tunneling strategy that uses storyline scenarios to guide a player throughout the games.

#### 4.5.9. Reward Strategy

The reward strategy was exemplified as badges in the “BunnyBolt” game [102]. A virtual trophy or stars were used in the “Polar FT60” system as rewards [55]. Intelligent musical stairs known as “Social Stairs” were implemented as a reward strategy by triggering music corresponding to the user's steps on stairs [110]. As shown in Figure 7A, there were seven visual growth levels for the virtual fish in the “Fish in Steps” desktop game and a happy facial expression of virtual fish was used as reward and tracking/self-monitoring strategies for users [39].

#### 4.5.10. Priase Strategy

The praise strategy was used as an encouraging text message (“Keep walking! You can do it!”) [85]. Another example of a praise strategy to motivate users to do more PA was shown in the heart rate monitor system called “Polar FT60” by delivering encouraging verbal feedback such as “Maximal performance improving,” “Well done!,” or “Excellent!” [55].

#### 4.5.11. Tailoring Strategy

The tailoring strategy was employed in the mobile phone text messaging system [54] by providing information and tips on the PA and healthy eating domains tailored to African-American women who participated in a weight management program. Similarly, a tablet-based application called “Agile Life” was designed to be tailored to the elderly by giving them PA information chunks [81]. Another example of a tailoring strategy was used by micro-blogging sites like “Twitter,” which was tailored to encourage teenage girls to exercise through the use of social media supports [68].

#### 4.5.12. Reduction Strategy

The reduction strategy was used in different ways as represented in the reviewed articles (e.g., targeting simple behavior such as stretching and walking) as shown in the “WragaFit” application [157]. It was also seen in the “LocoSnake” game [76], as users could select the level of the game from three difficulty levels (easy, medium, and hard). In addition, a reduction strategy was represented, for example, in the “CrowdWalk” mobile application [139], since the application provides a list of a location-based “walking challenges” through the use of a map visualization to give the user an easy way to engage in nearby activities and challenges.

#### 4.5.13. Social Comparison and Social Learning Strategies

**Figure 8:**
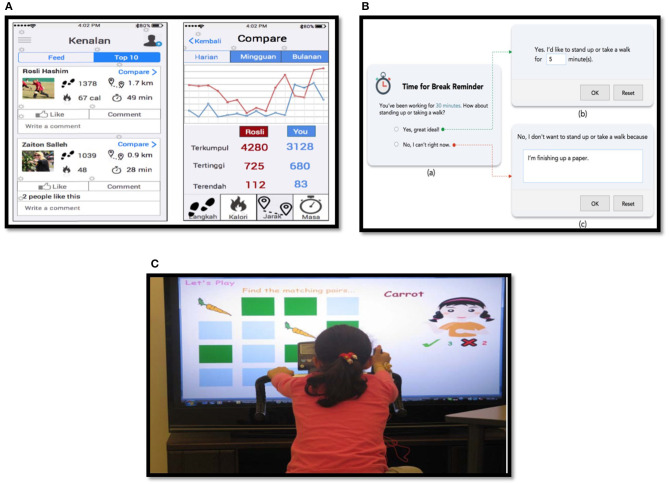
**(A)** Social comparison and social learning in wragaFit application [157]; **(B)** Time for break system [190]; **(C)** Exerlean bike system [72].

The social comparison and social learning were used in the “WragaFit” smartphone application [157] as highlighted in Figure 8A. Another example of a social learning strategy was the “Pediluma” shoe activity tracker device that monitored the wearer's movements by providing varying intensities of a lighted cage [69].

#### 4.5.14. Social Cooperation Strategy

The social cooperation was used in the tablet application “Agile Life” [81] to enable elderly users to engage with friends in PAs. Simulation and social comparison strategies were used as a mechanism on a group and individual level with the assistance of Facebook as in the “Active2Gether” system, so a user was able to compare his/her performance with others and notice the link between a cause and effect [175].

#### 4.5.15. Social Competition and Social Recognition Strategies

Social competition and social recognition “ranking” strategies were used, such as in a Facebook application called “StepMarton” [63] that displays the entire number of steps for each user and his/her name in an order from the user with the highest number of steps on the top to the user with the smallest number of steps on the bottom.

#### 4.5.16. A Real-World Feel Strategy

The example of a real-world feel strategy was shown in the “WragarFit” system by enabling users to accomplish each other's tasks on a “news feed” [157].

#### 4.5.17. Social Facilitation Strategy

The social facilitation strategy was implemented in a mobile lifestyle coaching application [38] by allowing the achievements of an individual team member to be visible to the rest of the team and the achievement of the entire team to be visible to all members of a team and other teams.

#### 4.5.18. Normative Influence Strategy

The normative influence strategy was employed in the “SitCoach” application since it stores the number of active minutes daily for each user and gives a notification for all users to observe the progress of each other [113].

#### 4.5.19. Personalization Strategy

The personalization strategy was employed in the “StepMarton” application [63] by providing personalized Facebook notifications and in the “Alert Me” mobile application by delivering timely personalized messages to users and by allowing users to create personal profiles [169].

#### 4.5.20. Self-Report Strategy

The self-report strategy was represented as feedback from a user to the system, such as in the “Time for Break” system [190], as a user provides feedback when responding to the reminders. Users in such a situation respond to the reminder question with either “Yes” or “No,” and when choosing “Yes,” the users enter a desirable duration for a break, and when they choose “No,” the users type the reasons for not taking a break as shown in Figure 8B. The smart-watch application in the “ROAMM” monitoring system was used to collect self-reported data from users [158].

#### 4.5.21. A Similarity Strategy

A similarity strategy was employed in the “WragaFit” system by providing images to older workers to make them feel familiar [157].

#### 4.5.22. A Reminder Strategy

An example of a reminder strategy was mentioned in the “Time for Break” system [190] by issuing a textual notification as a question to a user (e.g., “You have been working for 30 min. How about taking a walk or standing up?”) as shown in Figure 8B. Another instance of a reminder was represented in the “SitCoach” mobile application as an acoustic (buzzing) alert, a textual message, and a tactile reminder (vibration) [113]. A musical reminder in the “FLOW Pillow” system for the elderly was another way of implementing a reminder strategy [160].

#### 4.5.23. Surface Credibility Strategy

The surface credibility strategy was employed in the “PersonA” system (a persuasive social network for PA) by providing security, confidentiality, and privacy features in the system [74]. Furthermore, a smartphone and web game known as “Phone Row” implemented a surface credibility strategy by offering a security mechanism through generating a new identifier for a present computer screen every time a user visits the webpage. In addition, a user was also required to scan a QR-code on the website [91].

#### 4.5.24. Rehearsal Strategy

The rehearsal strategy was used by providing a video tutorial to educate users on appropriate techniques for doing stretching at the workplace [157].

#### 4.5.25. Expertise Strategy

The expertise strategy was used by delivering healthy tips and information to older workers from an official medical source or fitness experts [157].

#### 4.5.26. Verifiability Strategy

The verifiability strategy was implemented in the “WragaFit” application, as users were able to verify the source of the provided health tips and information through an external link [157].

#### 4.5.27. Trustworthiness Strategy

The trustworthiness strategy was implemented in the “Polar FT60” heart rate monitor because Polar is a trustworthy source of information [55].

#### 4.5.28. Liking Strategy

The liking strategy was clearly shown in the Exerlean Bike System [72], as it provides children with attractive audial, textual, and visual representations for both Memory and ExerMath games and through the use of sensors and a stationary bike to easily enable the children to do PAs while responding to the games' assignments, as shown in Figure 8C. Other examples of liking and expertise strategies are found in the “Active2Gether” system when an expert designer was hired to design and provide recommendations on diverse aspects of the user interface to give the system an appropriate look and feel for the users [175].

#### 4.5.29. Social Role Strategy

The reasoning engine feature in the “Active2Gether” system implemented a social role strategy by providing communication dialogue between the users and the system, which contains messages or questions for the users [175]. Moreover, a social role strategy was implemented as a “personal trainer” to guide users' movements by giving verbal and personalized feedback as mentioned in the “Polar FT60” system [55].

### 4.6. Comparative Effectiveness by the Persuasive Strategies

**Figure 9:**
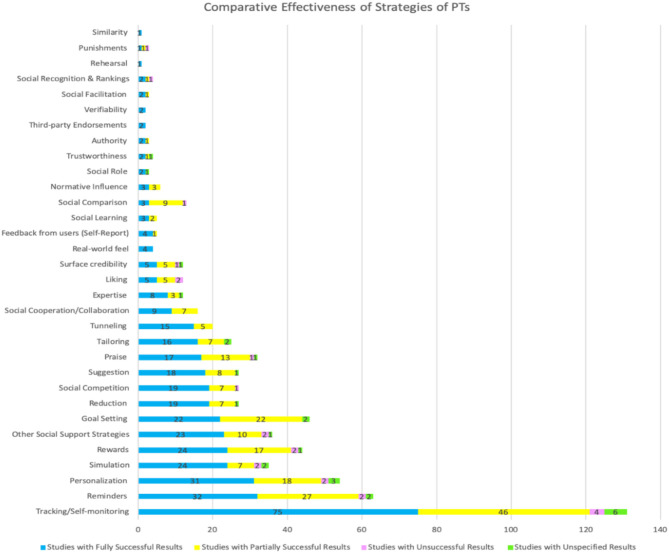
Comparative effectiveness of persuasive strategies.

Table 6 and Figure 9 show the comparative effectiveness of PTs using persuasive strategies in the domain of PA and SB. The table and figure indicate that some strategies were applied more frequently and some tend to be more effective than others. For example, the tracking and self-monitoring strategy was employed in 153 (19%) studies, with a total of 75 (49%) studies reporting fully successful outcomes, 46 (30%) studies reporting partially successful outcomes, four (2%) studies reporting unsuccessful outcomes and six (4%) studies not specifying their outcomes, while 23 (15%) studies did not evaluate their strategies.

**Table 6:** Comparative effectiveness of persuasive strategies of persuasive technology.

**#**	**Motivational strategies/Affordances**	**Total number of studies with fully successful results**	**Total number of studies with partially successful results**	**Total number of studies with unsuccessful results**	**Total number of studies with unspecified results**	**Total number of articles with no evaluation (none)**
1	Tracking/Self-monitoring	75	46	4	6	22
2	Reminders	32	27	2	2	9
3	Personalization	31	18	2	3	10
4	Simulation	24	7	2	2	7
5	Rewards	24	17	2	1	9
6	Other social support strategies	23	10	2	1	7
7	Goal setting	22	22	0	2	7
8	Reduction	19	7	0	1	5
9	Social competition	19	7	1	0	3
10	Suggestion	18	8	0	1	3
11	Praise	17	13	1	1	6
12	Tailoring	16	7	0	2	4
13	Tunneling	15	5	0	0	5
14	Social cooperation/Collaboration	9	7	0	0	6
15	Expertise	8	3	0	1	1
16	Liking	5	5	2	0	2
17	Surface credibility	5	5	1	1	6
18	Real-world feel	4	0	0	0	0
19	Feedback from users (Self-Report)	4	1	0	0	1
20	Social learning	3	2	0	0	2
21	Social comparison	3	9	1	0	4
22	Normative influence	3	3	0	0	1
23	Social role	2	0	0	1	3
24	Trustworthiness	2	1	0	1	1
25	Authority	2	1	0	0	2
26	Third-party endorsements	2	0	0	0	2
27	Verifiability	2	0	0	0	0
28	Social facilitation	2	1	0	0	0
29	Social recognition & Rankings	2	1	1	0	2
30	Rehearsal	1	0	0	0	0
31	Punishments	1	1	1	0	1
32	Similarity	1	0	0	0	0

In summary, we reported the top twelve persuasive strategies most frequently used in the domain of PA and SB with respect to their effectiveness. As represented in Table 6 and Figure 9, out of the total studies that implemented each persuasive strategy (see Section 4.4), tracking and self-monitoring ranked first with a total of 121 (79%) successful outcomes, followed by reminder and personalization, which ranked second and third with 59 (82%), and 58 (91%) successful results, respectively. Goal-setting came at fourth with 44 (83%) successful outcomes. Rewards ranked at fifth with 41 (76%) successful results. Other social support strategies ranked 6th with total numbers of 33 (77%) successful studies. Simulation and praise were at 7th and 8th with 31 (84%) an 30 (79%) successful studies, respectively. Reduction, social competition, and suggestion came in the 9th place with total numbers of 26 (81%) successful studies for each. Tailoring, tunneling, and expertise ranked 10, 11, 12th with 23 (79%), 20 (80%), and 11(85%) successful studies, respectively.

Generally, we noticed that the five most effective persuasive strategies employed were tracking/self-monitoring, reminders, personalization, goal-setting, rewards, and other social support strategies. Furthermore, if we consider the employment of all social support strategies as overall (e.g., social learning, social cooperation, social comparison, social competition, normative influence, social facilitation, social recognition, and other social support strategies), we can notice that the second most effective and commonly employed set of strategies were social support strategies which were mainly used as external motivations to persuade users to engage more in increasing their PA levels and reducing SB.

### 4.7. Behavior Theories Employed and the Effectiveness of PTs

**Figure 10:**
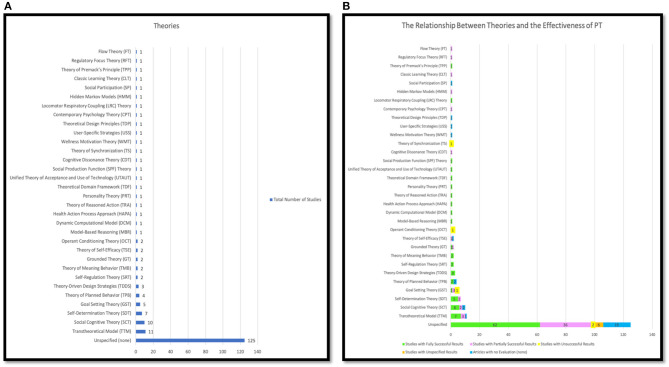
**(A)** Behavior theories used in persuasive technology design; **(B)** the relationship between behavior theory and the effectiveness of PT.

Evaluating the studies based on the behavior theories they employed shows that 125 studies, approximately three quarters (74%) did not have any theory informing their design of the PTs, as shown in Table 7 and Figure 10A. However, among the studies employing theories, many of the studies also only mentioned the theories without providing details of how they informed the study and design of the PT. Table 7 and Figure 10A show that the Transtheoretical model of change (TTM) was the most frequently employed in the studies that were analyzed, with a total of 11 (6%) of studies. Social cognition theory and self-determination theory were second and third with a total of 10 (6%) and 7 (4%) studies respectively. Furthermore, many of the studies used more than one theory, or adapted more than one theory to guide the PT design.

**Table 7:** Behavior theories used in persuasive technology design.

**Theories**	**Study**	**Total number of studies**	**Average out of % 170 for each**
Transtheoretical model (TTM)	[39, 42, 44, 69, 82, 84, 85, 94, 135, 175, 191]	11	6%
Goal setting theory (GST)	[84, 107, 135, 186, 197]	5	3%
Theory of planned behavior (TPB)	[60, 74, 149, 161]	4	2%
Social cognitive theory (SCT)	[42, 74, 82, 94, 116, 126, 175, 191, 199, 203]	10	6%
Theory-driven design strategies (TDDS)	[80, 95, 149]	3	2%
Model-based reasoning (MBR)	[175]	1	1%
Dynamic computational model (DCM)	[175]	1	1%
Self-regulation theory (SRT)	[149, 175]	2	1%
Health action process approach (HAPA)	[175]	1	1%
Theory of reasoned action (TRA)	[103]	1	1%
Theory of meaning behavior (TMB)	[50, 60]	2	1%
Personality theory (PT)	[60]	1	1%
Theoretical domain framework (TDF)	[154]	1	1%
Self-determination theory (SDT)	[50, 57, 94, 143, 149, 153, 178]	7	4%
Unified theory of acceptance and use of technology (UTAUT)	[144]	1	1%
Grounded theory (GT)	[58, 172]	2	1%
Social production function (SPF) theory	[56]	1	1%
Cognitive dissonance theory (CDT)	[112]	1	1%
Theory of synchronization (TS)	[108]	1	1%
Wellness motivation theory (WMT)	[106]	1	1%
User-specific strategies (USS)	[106]	1	1%
Theoretical design principles (TDP)	[106]	1	1%
Contemporary psychology theory (CPT)	[132]	1	1%
Locomotor respiratory coupling (LRC) theory	[131]	1	1%
Hidden markov models (HMM)	[42]	1	1%
Theory of self-efficacy (TSE)	[82, 104]	2	1%
Social participation (SP)	[82]	1	1%
Classic learning theory (CLT)	[52]	1	1%
Operant conditioning theory (OCT)	[75, 107]	2	1%
Theory of Premack's principle (TPP)	[75]	1	1%
Regulatory focus theory (RFT)	[183]	1	1%
Flow theory (FT)	[160]	1	1%
Unspecified (none)	[34–38, 40, 41, 43, 45–49, 51]	125	7%
	[53–55, 59, 61–68, 70–73, 76–78]		
	[79, 81, 83, 86–93, 96–99, 111, 118, 151]		
	[100–102, 105, 109, 110, 113–115, 117, 119–125, 127]		
	[128–130, 133, 134, 136–142, 145, 147, 148, 150, 152, 174]		
	[146, 155–159, 162–171, 173, 176, 179]		
	[177, 180–182, 184, 185, 187–190, 192–196, 198, 200–202]		

As shown in Figure 10B, based on our analysis, a total of 98 (78%) of all the studies employing no theory reported successful outcomes, whether fully or partially successful, while only 2 (2%) reported unsuccessful results. Nineteen of the studies that did not employ any theory conducted no evaluations. With respect to the studies employing theories (45 studies), 39 (86%) reported successful results, whether fully or partially successful, while 2(4%) reported unsuccessful results. Four of the studies that employed theories conducted no evaluations. We could not precisely compare the effectiveness of PTs employing behavior theories and those that did not because of the limited number of studies employing theory. However, we noticed that although limited, the studies employing theory in their design seem to be more effective compared to those that are not based on any theory.

### 4.8. Targeted Health Behavior Domain

**Figure 11:**
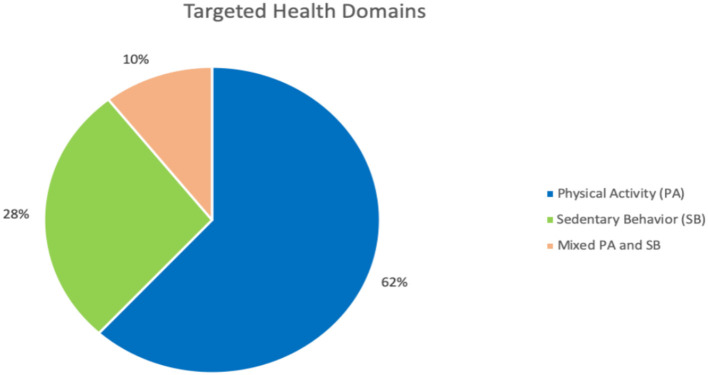
Targeted health domain.

In this study, all the articles selected for review were those that targeted PA and/or SB. Table 8 and Figure 11 illustrate how we categorized the health domains in this paper into three groups based on the main objective of each study. One hundred and five (62%) of the studies focused on increasing physical activity (PA) levels, and 47 (28%) studies focused on mitigating sedentary behavior. Eighteen (11%) studies aimed to both increase PA levels and reduce SB. In general, the domains covered in this paper are classified into two main categories, PA and/or SB.

**Table 8:** Targeted health domains.

**Domain**	**Total**	**Overall of 170**
Physical Activity (PA)	105	62%
Sedentary Behavior (SB)	47	28%
Mixed PA and SB	18	11%

### 4.9. Targeted Behavioral and/or Psychological Outcomes

**Figure 12:**
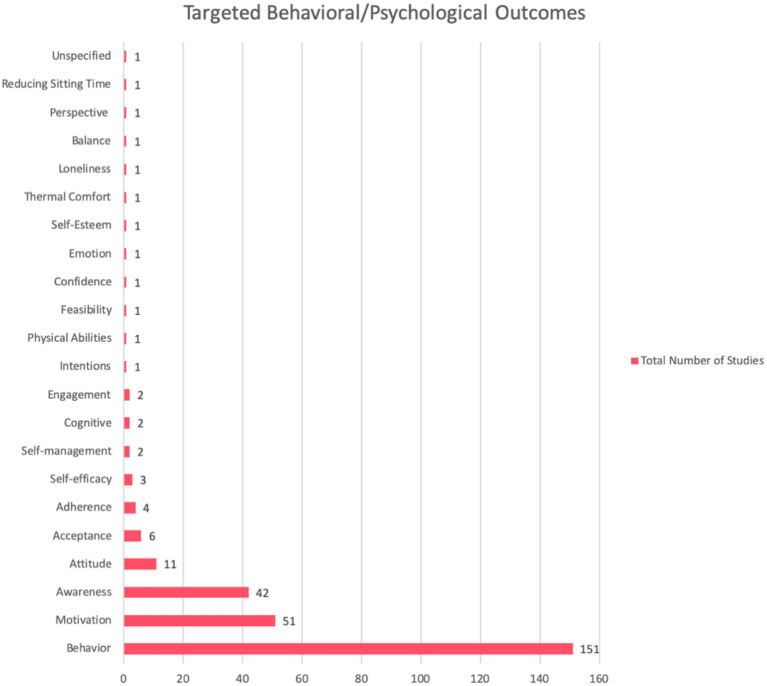
Targeted psychological and behavioral outcomes of persuasive technologies.

Table 9 and Figure 12 display the behavioral and psychological outcomes targeted by the reviewed articles.The articles targeted 21 diverse outcomes as most of the reviewed studies targeted more than one behavioral and/or psychological outcome. Almost three quarters of the studies 151 (89%) were targeted at actual behavior change, which consists of promoting/encouraging a shift from undesirable behavior and habit [4], promoting physical activity and discouraging SB. We found that 51 (30%) of the studies targeted a change in motivation, 42 (25%) increased the awareness for the users, and 11 (6%) focused on changing the attitude of the individuals. Several of the studies targeted the emotions, loneliness, adherence, intentions, and self-efficacy of the individual, as shown in Table 9 and Figure 12. The category “Unspecified” refers to those studies that did not specify the targeted behavioral and/or psychological outcomes. It is also important to note that most of the studies targeted more than one behavioral outcome, which means that many of the studies belonged to more than one category. For example, one study could be targeting the behavior and attitude change in the user.

**Table 9:** Targeted psychological and behavioral outcomes by persuasive technology.

**Targeted outcomes**	**Study**	**Total number of studies**	**Average out of % 170 for each**
Behavior	[42, 52, 60–62, 73–75, 92, 132, 144, 177–180]; [198] [34–36, 39, 41, 43–46, 48, 51, 53, 55–59, 63–71, 77–91, 93–115, 118, 120–131, 133–142, 145–158, 160–165, 167–176, 182–187, 189–197, 199, 202, 203]	151	89%
Awareness	[35, 39, 42, 43, 45, 47, 49, 69, 70, 80–82, 87, 94, 95, 99, 112, 113, 115, 123, 138–141, 144, 145, 158, 161, 169, 170, 172, 175, 184, 187, 191–193, 200] [41, 67, 202, 203]	42	25%
Motivation	[36, 37, 42, 44, 47, 50, 52, 53, 57, 60, 61, 65, 69, 75, 81, 95, 100, 103, 110–114, 116, 119, 129, 134, 136, 141, 143, 145, 162, 166, 171, 177–179, 181, 183, 185, 186, 189, 191–194]; [195, 197–200]	51	30%
Self-management	[159, 187]	2	1%
Attitude	[39, 54, 69, 76, 103, 117, 122, 132, 191–193]	11	6%
Adherence	[74, 182, 188, 196]	4	2%
Intentions	[190]	1	1%
Cognitive	[72, 83]	2	1%
Physical abilities	[72]	1	1%
Feasibility	[154]	1	1%
Acceptance	[38, 154, 182, 188, 192, 193]	6	4%
Confidence	[194]	1	1 %
Emotion	[56]	1	1 %
Self-Esteem	[56]	1	1 %
Thermal comfort	[201]	1	1 %
Loneliness	[56]	1	1 %
Balance	[56]	1	1 %
Engagement	[48]	1	1%
Perspective	[174]	1	1%
Reducing sitting time	[126]	1	1%
Self-efficacy	[36, 57, 194]	3	2%
Unspecified	[40]	1	1%

### 4.10. Study Methodology Used by Persuasive Technology

**Figure 13:**
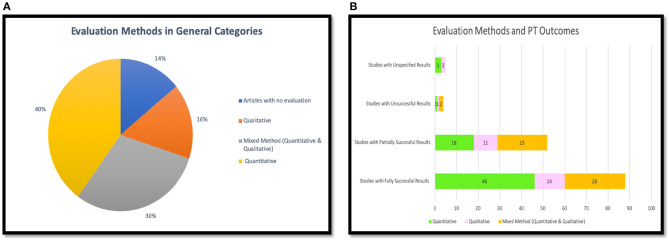
**(A)** Evaluation methdologies employed by persuasive technology; **(B)** Evaluation methods and persuasive technology effectiveness.

Table 10 and Figure 13A demonstrate the frequency of the study methodologies used by PTs in the reviewed studies. The quantitative method was the most common methodology employed in the reviewed studies, with a total of 68 (40%). The mixed method was the second most common method used, with a total of 51 (30%) studies. Of the reviewed studies, 28 (16%) studies used a fully qualitative method. The most common quantitative approaches used for data gathering were the use of activity trackers, monitors, and sensors devices, and the use of other systems capable of gathering quantitative data of users' behaviors such as step counters. Moreover, the questionnaire/survey was used as a quantitative method to collect numeric data. The qualitative methods used in the PA and/or SB studies include observations of users' performance, interviews, and focus groups. Although the mixed method (a combination of qualitative and quantitative methodologies) ranked as the second most commonly employed evaluation approach, it is considered as the most comprehensive approach to analyzing the PT design outcomes. Therefore, we recommend researchers to apply the mixed evaluation methodology over a qualitative methodology alone or a quantitative methodology alone.

**Table 10:** Evaluation methods and persuasive technology outcomes.

**Evaluation method**	**Number of studies with fully successful results**	**Number of studies with partially successful results**	**Number of studies with unsuccessful results**	**Number of studies with unspecified results**	**Total**	**Overall of % 170**
Quantitative	46 (68%)	18 (27%)	1 (1%)	3 (4%)	68	40%
Qualitative	14 (50%)	11 (39%)	1 (4%)	2 (7%)	28	16%
Mixed method (Quantitative & Qualitative)	28 (55%)	23 (45%)	2 (4%)	0	51	30%
Number of articles with no evaluation (none)	23				23	14%

### 4.11. Evaluation Methods and Persuasive Technology Effectiveness

As Table 11 and Figure 13B illustrate, out of the 68 studies that employed a quantitative evaluation, 46 (68%) reported fully successful outcome, 18 (27%) partially successful outcomes, 1 (1%) an unsuccessful study, and 3 (4%) were studies that did not specify their outcomes. However, of the studies that used the mix of quantitative and qualitative evaluation methodologies, a total of 28 (55%) were fully successful, 23 (45%) were partially successful, and 2 (4%) were unsuccessful. The studies which implemented just a qualitative methodology have the least effective outcomes, with a total of 14 (50%) completely successful studies, 11 (39%) partially successful, 1 (4%) unsuccessful, and 2 (7%) included studies with unspecified outcomes.

**Table 11:** Evaluation methodologies employed by persuasive technology and PT effectiveness.

**Evaluation method**	**Studies with fully successful results**	**Studies with partially successful results**	**Studies with unsuccessful results**	**Studies with unspecified results**	**Total**	**Overall of % 170**
Quantitative	[37, 38, 45, 47, 50, 56, 57, 61, 62, 68, 73, 75, 79, 89, 92–95, 109, 114, 117, 118, 124, 126, 128, 130, 131, 136, 141, 144, 146–149, 151, 155, 156, 158, 163, 166, 169, 173, 176, 196, 197, 203]	[53, 59, 71, 77, 96, 105, 113, 120, 125, 135, 140, 142, 143, 164, 170, 177, 181, 201]	[165]	[98, 138, 152]	68	40%
Qualitative	[49, 50, 58, 60, 67, 80, 110, 121, 153, 154, 174, 175, 188]	[36, 43, 81, 84, 99, 123, 132, 172, 183, 187, 191]	[91]	[55, 97]	28	16%
Mixed method (Quantitative & Qualitative)	[34, 35, 39–41, 44, 46, 48, 54, 63, 64, 66, 69, 70, 72, 76, 83, 85, 86, 100, 103, 115, 116, 122, 137, 139, 157, 184]	[42, 51, 52, 101, 104, 112, 129, 133, 134, 160, 167, 178–180, 182, 185, 186, 190, 192, 193, 195, 199, 202]	[107, 108]		51	30%
Articles with no evaluation	[65, 74, 78, 82, 87, 88, 90, 102, 106, 111, 119, 127, 145, 150, 159, 161, 162, 168, 171, 189, 194, 198, 200]				23	14%

*Conference Name:ACM Woodstock conference*.

### 4.12. Study Participants and Sample Size

**Figure 14:**
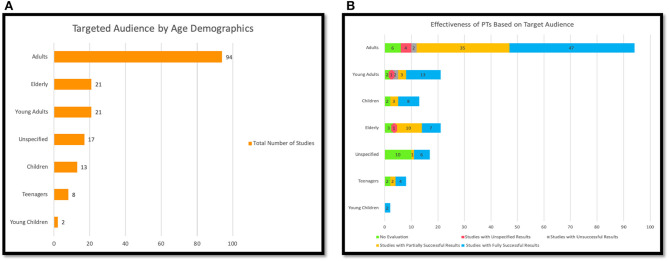
**(A)** Targeted audience by age demographics; **(B)** Effectiveness and evaluation outcomes of PTs based on target audience.

The sample size varies greatly among the studies reviewed, as the mean number of subjects was 798 with a minimum of one subject and a maximum of 129,010 participants. There are also some studies that did not report the total number of participants, whereas others also had varying sample sizes at different stages of the PT evaluation. Table 12 and Figure 14A show the targeted audience by age demographic, whereas Table 13A and Figure 14B present the effectiveness of the interventions depending on the targeted audience. We found that most of the studies (94, or 53%) targeted the adults with most of them reporting successful results. This was followed by 21 (12%) studies that targeted young adults and elderly people. However, only 13 studies (8%) targeted children, 8 studies (5%) targeted teenager, and 2 studies (1%) targeted young children. We also found 17 (10%) studies that did not specify their audience. The most targeted populations were adults and young adults, while the least were older people, children, teenagers, and young children.

**Table 12:** Targeted audience by age demographic.

**Audience category**	**Study**	**Total number of studies**	**Average out of % 170 for each**
Young children	[66, 83]	2	1%
Children	[36, 61, 64, 67, 72, 75, 101, 104, 122, 141, 193, 198, 200]	13	8%
Teenagers	[41, 50, 51, 60, 68, 82, 192, 198]	8	5%
Young adults	[37, 44–46, 55, 76, 82, 85, 86, 102, 107, 108, 134, 136, 148, 153, 155, 175, 181, 192, 203]	21	12%
Adults	[34–36, 38, 39, 42, 43, 47, 49, 53, 54, 57–59, 62, 63, 65, 69–71, 73, 77, 79, 80, 84, 87, 89, 91–99, 103, 105, 109, 111, 113–117, 120, 121, 123, 125, 126, 128–132, 135, 137–140, 142–144, 146, 147, 149, 151, 152, 154, 158, 161, 163–165, 167, 168, 170, 172–174, 176, 178, 179, 184–186, 189–192, 195, 199, 201, 202]	94	55%
Elderly	[52, 166, 177, 180] [40, 48, 56, 78, 81, 100, 106, 112, 119, 133, 157, 160, 182, 187, 188, 192, 197]	21	12%
Unspecified	[74, 88, 90, 110, 118, 124, 127, 145, 150, 156, 159, 162, 169, 171, 183, 194, 196]	17	10%

**Table 13:** **(A)** Targeted audience by age group and persuasive technologies effectiveness; **(B)** Effectiveness of persuasive technologies based on targeted audience.

**(A)**								
**Targeted audience by age group**	**Age group (years old)**	**Studies with fully successful results**	**Studies with partially successful results**	**Studies with unsuccessful results**	**Studies with unspecified results**	**Articles with no evaluation**	**Total number of studies**	**Average out of 170 for each**
Young children	4 to 7	[66, 83]					2	1%
Children	8 to 12	[61, 64, 72, 75, 101, 122, 141] [67]	[36, 104, 193]			[198, 200]	13	8%
Teenagers	13 to 17	[41, 50, 60, 68]	[51, 192]			[82, 198]	8	5%
Young adults	18 to 30	[37, 44–46, 76, 85, 86, 136, 148, 153, 155, 175, 203]	[134, 181, 192]	[107, 108]	[55]	[82, 102],	21	12%
Adults	31 to 49	[34, 35, 38, 39, 47, 49, 54, 57, 58, 62, 63, 69, 70, 73, 79, 80, 89, 92–95, 103, 109, 114–117, 121, 126, 128, 130, 131, 137, 139, 144, 146, 147, 149, 151, 154, 158, 163, 173, 174, 176, 184, 191]	[36, 42, 43, 53, 59, 71, 77, 84, 96, 99, 105, 113, 120, 123, 125, 129, 132, 135, 140, 142, 143, 164, 167, 170, 172, 178, 179, 185, 186, 190, 192, 195, 199, 201, 202]	[91, 165]	[97, 98, 138, 152]	[65, 87, 111, 161, 168, 189]	94	55%
Elderly	50 and above	[48, 56, 100, 157, 166, 188, 197]	[52, 81, 112, 133, 160, 177, 180, 182, 187, 192]		[40]	[78, 106, 119]	21	12%
Unspecified	Not specified	[110, 118, 124, 156, 169, 196]	[183]			[74, 88, 90, 127, 145, 150, 159, 162, 171, 194]	17	10%

**Table:** 

**(B)**					
**Targeted audience by age group**	**Number of studies with fully successful results**	**Number of studies with partially successful results**	**Number of studies with unsuccessful results**	**Number of studies with unspecified results**	**Number of articles with no study (none)**
Young children (4 to 7)	2	0	0	0	0
Children (8 to 12)	8	3	0	0	2
Teenagers (13 to 17)	4	1	0	0	2
Young adults (18 to 30)	13	3	2	1	2
Adults (31 to 49)	47	34	2	4	6
Elderly (50 and above)	7	6	0	1	3
Unspecified	6	0	0	0	10

Young children include kids in the age group 4 to 7, children in the age group 8 to 12, teenagers from 13 to 17 years old and young adults from 18 to around 30 years old. Adults have a wide age range and could start from 31 to 49 years old, whereas the elderly were 50 years old and above.

### 4.13. Effectiveness of PTs Based Targeted Audience's Age Group

Tables 13A,B and Figure 14B demonstrate the effectiveness of employing PT with regards to the targeted audience's age group. For adults, we found that 81(86%) of the studies reported successful results; that is, studies with partially successful and those with fully successful results. Specifically, 47 (58%) studies were fully successful, and 34 (42%) studies were partially successful. For young adults, out of 21 studies targeted at them, 13 (61%) showed fully successful outcomes, 3 (14%) displayed partially successful outcomes, just 2 (10%) reported unsuccessful outcomes, and only 1 (5%) represented unspecified outcomes, and 2 (10%) did not provide evaluations. For the elderly, out of 21 studies targeted at them, 7 (33%) reported fully successful results, 10 (48%) showed partially successful results, only 1 (5%) reported unspecified results, and 3 (14%) did not evaluate their studies. For children, out of 13 studies targeted at them, 8 (62%) reported fully successful results, 3 (23%) showed partially successful results, and just 2 (15%) did not evaluate their PTs. For teenagers, out of 8 studies targeted at them, 4 (50%) reported fully successful results, 2 (25%) provided partially successful results, and only 2 (25%) did not conduct any evaluations. Only two studies provided fully successful outcomes for young children. Therefore, the most successful outcomes for implementing the PTs were observed in the studies targeting adults and young adults.

### 4.14. Targeted Audience by Their Occupation/Status or Health Condition

**Figure 15:**
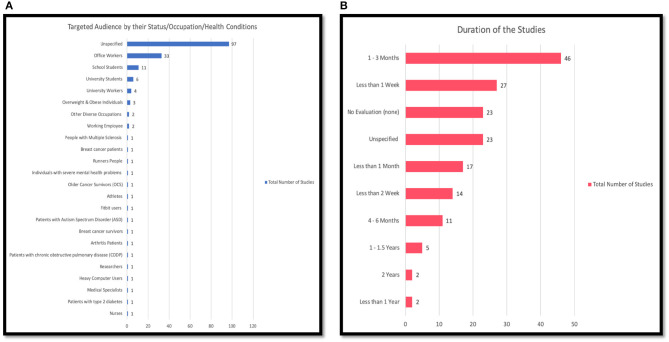
**(A)** Audience categorization based on their occupation/health conditions; **(B)** Duration of studies' evaluation.

Another classification of the targeted audience was based on the audience's situation, such as their occupation and health conditions, as we found from the reviewed studies. As Table 14 and Figure 15A show, 97 (57%) studies did not specify their sample population's status. Thirty-three (19%) studies are targeted at office workers, 11 (6%) at students (e.g., primary school students, and high school students), 6 (4%) at university students (e.g., undergraduate students, and graduate students), 4 (2%) at university workers (e.g., university staff and faculty members), and 3 (2%) studies were targeted at people with overweight and obesity conditions. Nurses, researchers, runners, employees, heavy computer users, medical specialists, patients with type 2 diabetes, patients with chronic obstructive pulmonary disease, breast cancer survivors, arthritis patients, patients with autism spectrum disorder, Fitbit users, older cancer survivors, individuals with severe mental health problems, breast cancer patients, and people with multiple sclerosis were the target of one (1%) of the study each. Consequently, approximately a quarter of all studies focused on office workers because these populations are more likely to remain sitting on their seats and working on their desks for long hours. In such a situation, it is possible for them to suffer some lifestyle-related health issues such as cardiovascular disease, cancer, obesity, and diabetes.

**Table 14:** Audience occupation /status/ health conditions.

**Audience occupation or health condition**	**Study**	**Total number of studies**	**Average out of % 170 for each**
School students	[50, 72, 75, 94, 101, 104, 122, 141, 181, 193, 200]	11	6%
University students (Undergraduate Students, Graduate students)	[86, 123, 134, 179, 190, 202]	6	4%
Office workers	[35, 43, 49, 71, 73, 79, 89, 93, 95, 98, 99, 109, 111, 113, 126, 130, 133, 142, 146, 147, 149, 151, 152, 154, 157, 161, 163–165, 167, 174, 195, 201]	33	19%
Nurses	[63]	1	1%
University workers (Information workers as University staff members, student council) and other workers	[96, 105, 178, 190]	4	2%
Patients with type 2 diabetes	[140]	1	1%
Medical specialists	[138]	1	1%
Heavy computer users	[116]	1	1%
Researchers	[94]	1	1%
Overweight & obese individuals	[59, 114, 166]	3	2%
Working employee	[77, 153]	2	1%
Patients with chronic obstructive pulmonary disease (CODP)	[182]	1	1%
Arthritis patients	[187]	1	1%
Breast cancer survivors	[191]	1	1%
Patients with autism spectrum disorder (ASD)	[189]	1	1%
Fitbit users	[186]	1	1%
Athletes	[143]	1	1%
Older cancer survivors (OCS)	[100]	1	1%
Individuals with severe mental health problems	[185]	1	1%
Runners people	[132]	1	1%
Breast cancer patients	[168]	1	1%
People with multiple sclerosis	[197]	1	1%
Diverse occupations (e.g., Administrator, Human resources specialist, Economist, Engineer, Educator, & real estate agent)	[129, 179]	2	1%
Unspecified	[34, 36–42, 44–48, 51–58, 60–62, 64–70, 74, 76, 78, 80–85, 87, 88, 90–92, 97, 102, 103, 106–108, 110, 112, 115, 117–121, 124, 125, 127, 128, 131, 135–137, 139, 144, 145, 148, 150, 155, 156, 158–160, 162, 169–173, 175–177, 180, 183, 184, 188, 192, 194, 196, 198, 199, 203]	97	57%

### 4.15. Duration of Evaluation

The duration of the studies varied from 1 day to ~2 years. In addition, 23 (14%) studies did not report how long they evaluated their persuasive technologies. The results indicate that 46 (27%) studies evaluated the PT from 1 to 3 months, 27 (16%) studies for <1 week, and 17 (10%) studies for <1 month, 14 (8%) studies for <2 weeks, and 11 (6%) studies for four to six months, and just 2 (1%) studies for <1 year. Only 5 (3%) studies conducted their long-term “longitudinal” evaluations of the effectiveness of PTs for one to a one and a half years, and 2 (1%) for 2 years. The results also reveal that 20 (11%) studies with a longitudinal evaluation have a duration from 4 months to 2 years, whereas 104 (61%) studies conducted their PTs over a duration from <1 week to 3 months. The variation in the duration of evaluating the PTs presents a challenge because it is difficult to establish the long-term effects of the PTs since many studies did not conduct an adequate evaluation and follow-up studies. Consequently, there is still a need to conduct more long-term evaluations of PTs design in the domain of PA and/or SB to examine users' adherence and commitment and establish PTs effectiveness over a long-term for sustained behavior change. Figure 15B presents the duration of the evaluation of the reviewed studies.

## 5. Discussion

The purpose of this study is (1) to evaluate the effectiveness of PT used to promote PA and reduce SB; (2) to summarize and highlight trends in the outcomes and employed technological platforms; and (3) to reveal pitfalls and gaps in the present literature that could be leveraged and used to inform the design of PT targeting physical activity and sedentary behavior.

### 5.1. Overall Effectiveness of PTs for Physical Activity and Sedentary Behavior

Overall, 137 (81%) of the articles that we reviewed in this study reported successful outcomes, whether fully or partially successful, which prove that PTs are effective tools to promote PA and decrease SB. Only 4 (2%) of the reviewed studies had unsuccessful outcomes. There were no common or specific reasons for the failure outcomes of these studies. Each study had a different situation and employed a different method, strategies, and technology that may contribute to unsuccessful outcomes. For example, one study failed in designing an appropriate smartphone virtual boat racing game to motivate people to engage more in Moderate-Intensity Physical Activity (MIPA). This is because the game was not implemented optimally, which caused users to suffer from some repetitive strain injuries and drove them to abandon the app [91]. Other studies implemented different technologies such as Persuasive Art reflection [107], and ExerSync by considering a rhythm of body movements [108]. Therefore, it is difficult to establish the actual reasons for the ineffectiveness of the PTs that reported unsuccessful results. Other reasons may be the target audience, their behavior change stage, and persuasive strategy mismatch, as highlighted [204].

### 5.2. The Relationship Between Technology Platforms and the Effectiveness of PTs

Mobile and handheld devices were the most dominant technology platforms used, with a total of 61 (36%) studies, followed by games, web and social networks, using of commercially available sensors and other activity trackers, custom-designed sensors and activity trackers, and ambient and public displays, which had a total of 33 (19 %), 32 (19%), 31 (18%), 19 (11%), and 16 (9%) studies respectively (see Figure 4A). Therefore, it is very clear that the second most dominant technologies employed in the reviewed studies were sensors and activity trackers and monitors devices, with a total of 50 (29%), either by using commercially available devices or designing new ones. In fact, if we consider the use of the embedded sensors in the smartphones and handheld devices such as GPS, GSM, gyroscope, accelerometer, pedometers, and cameras, we notice that the most important factor to motivate users in doing PA is to give accurate feedback and result of their activities tracked using sensors and activity trackers and monitors. This corroborates our findings, whereby the tracking and self-monitoring strategies ranked first with a total of 153 (90%) studies, of which 121 (79%) reported fully and/or partially successful outcomes, and the reminder strategy ranked second with a total of 72 (42%) studies, of which 32 (44%) had fully successful outcomes, and 27 (38%) had partially successful outcomes. These results suggest that a simple nudge such as a reminder to get some exercise (e.g., take some walk) or about how long they have been sitting down and the need to get up could motivate people to increase their physical activity. This is understandable, considering that in this modern time, people are always busy. So, even when they have the good intention to exercise and also know the consequences of living a sedentary lifestyle, they can easily forget. Therefore, a simple reminder could go a long way, motivating them to action.

As shown in Figure 4B, we found that the most successful outcomes for implementing the PTs were observed in the studies using the mobile and handheld devices, games, sensors and activity trackers in general, and websites and social networking sites (SNSs). It seems that these technologies are attractive and promising technologies for delivering interventions because of their ubiquitous nature.

### 5.3. The Relationship Between Behavior Theory and the Effectiveness of PT

As shown in Figure 10B, the findings reveal that almost three quarters 125 (74%) of all the reviewed articles did not use or did not state very clearly the behavior theory they used. Considering that most of the analyzed studies either did not specify the theories used to inform their design or did not use any theory, it is hard to draw conclusions on the relationship between employing behavior theory and the effectiveness of PTs. However, based on what we have, a total of 98 (78%) of all the studies employing no theory reported successful outcomes, whether fully or partially successful, while only 2 (2%) reported unsuccessful results. Nineteen of the studies that did not employ any theory conducted no evaluations. With respect to the studies employing theories (45 studies), 39(86%) reported successful results, whether fully or partially successful, while 2(4%) reported unsuccessful results. Four of the studies that employed theories conducted no evaluations. Based on this, it seems that the use of behavioral theories to inform PTs design increases the effectiveness of PTs with respect to achieving the intended objective of promoting PA or reducing SB.

### 5.4. Targeted Outcomes of Persuasive Technology

Most of the studies 151 (89%) targeted actual behavior change in the participants by increasing their level of physical activity, such as increasing step counts. User motivation was the second targeted outcome with a total of 51 (30%) studies, followed by articles that aimed at creating awareness and attitude change in users with totals of 42 (25%) and 11 (6%) studies, respectively. Nevertheless, there are some studies that targeted more than one behavioral or psychological outcomes.

### 5.5. The Relationship Between Persuasive Strategies and the Effectiveness of PTs

In the present review, various persuasive strategies were identified that were used to achieve positive behavior change. With respect to the studies employing persuasive strategies, and reported successful results, whether fully or partially, we found the most common strategies employed were tracking and self-monitoring with a total of 153 (90%) studies, of which 121 (79%) were successful studies. The implementation of such strategies was achieved by the use of diverse activity tracking and monitoring devices and sensors such as accelerometers, pedometers, heart rate monitoring devices and embedded sensors in smartphones, and by providing the user with his/her activity performance (e.g., step counts, heart rate, speed, summary progress) on the screen of the mobile phone devices using various display formats including visualization. PTs that used social support strategies (e.g., social comparison, social cooperation, social competition, normative influence, social facilitation, social learning, social recognition, and other social support strategies) were also effective in promoting physical activity with a total of 131 (77%) studies, of which 104 (79%) were studies of successful outcomes involving fully and partially. Overall, other strategies that were effective in addressing PA and SB includes: starting from the most effective to the least and out of the total studies that employed each persuasive strategy: reminders, personalization, goal setting, rewards, simulation, praise, reduction, suggestion, tailoring, tunneling, and expertise with a total of 59 (82%), 58 (90%), 44 (83%), 41 (76%), 31 (74%), 30 (79%), 26 (81%), 26 (87%), 26 (90%), 23 (92%), 20 (80%) and 11(85%) successful studies (whether fully or partially successful), respectively. These strategies were useful in encouraging users to make the appropriate changes in their behaviors and to be more aware and motivated.

It is also necessary to highlight the fact that most of the PT systems employed more than one strategy to achieve the targeted behavioral outcome. Also, the operationalization and implementation of these strategies varied from one application to another and may contribute to the effectiveness of the strategies. For example, some studies used a social support strategy as well as tracking, whereas others used the goal setting and reminder as different motivational strategies. In addition, the self-monitoring strategy came in various implementations, including graphical display, audio, textual, and visual feedback, ambient displays mirror, ambient sculpture display, and light displays.

Furthermore, the key implication from our findings is that there are considerable discrepancies in naming and implementing the persuasive strategies in the PT systems reviewed. Some PTs also implemented strategies that are not captured in the existing PSD framework. This makes it difficult to easily extract, identify, and name the strategies employed in PT. This makes the identification of such strategies to be based merely on the researchers' perspectives of PT. Although, there were diverse accomplishments in the research field in designing models that identify, classify, and name various persuasive strategies and their functionalities [3, 205]. Existing frameworks appear not to be comprehensive enough to capture all possible strategies in this considering the fast advancement of technology and opportunities that it creates to use various technology-enabled strategies that were probably not possible when existing models were developed. Therefore, we suggest that more work is needed in the area of developing a comprehensive PT design framework that captures all possible design strategies and various ways each can be operationalized in PT designs to achieve the desired behavioral outcome.

These findings agree with Orji and Moffatt [4]. As aforementioned, the persuasive system design (PSD) model seems not comprehensive enough to identify and classify all the strategies. As a result, we identify more strategies that were not included in the PSD.

### 5.6. The Relationship Between Targeted Audience and the Effectiveness of PT

Many PTs have been employed to persuade different age groups of users to change or adopt a desirable lifestyle with regard to PA. As displayed in Figure 14B, Tables 12, 13A, the reviewed studies showed that PT targeted at adults recorded the highest success rate, with 82 (87%) successful outcomes, of which 47 (58%) were fully successful outcomes and 34 (42%) were partially successful outcomes. Out of the total studies that targeted each age demographic, the second and third placed were elderly, and young adults, with a total of 17 (81%) and 16 (76%) of successful results studies, respectively. The fourth rank was children with 11 (85%) successful results. The studies that did not specify their target audience ranked 5th with 7 (41%) successful outcomes. The sixth and seventh placed were teenagers and young children with a total of 6 (75%) and 2 (100%) successful outcomes, respectively. As previously mentioned, the present study demonstrates that PT was most effective among adults when targeting PA and SB. However, it is important to note that the majority of the studies evaluated were targeted at the adult population; hence, comparing success rates across populations may not make much sense. A possible reason while most studies targeted adults reported successful results is that adults are in their active stage of life and at this stage, people tend to be more active naturally compare to the elderly group. Again, in comparison to children, adults tend to be more conscious about their life because they have the cognitive ability to understand the consequences of a sedentary lifestyle.

Furthermore, more than half of the reviewed articles did not specify their targeted audience occupation/status or health conditions, totaling 97 (57%) articles. However, 33 (19%) of the total articles were targeted at office workers. We believe that this is due to the nature of their jobs, which often lead to prolonged sitting (e.g., for hours) without taking frequent breaks to do some PA, such as stretching and walking. However, these articles did not specify their target audience's health situation beyond their occupation. One reason for this could be because conducting users' studies for the evaluation purposes of PT systems in adults and the general population without stating any conditions or restrictions is easier and more time-saving than conducting a study with a specific sample of users that have restricted criteria or specific health issues.

### 5.7. General Recommendations for Future Research

The review identified a number of limitations and gaps in the existing works in the area of PT for PA and SB. We offer suggestions for advancing research in this area:

1. **Standard Approach for Evaluating Persuasive Technology**: There is a need for a standard approach for evaluating the effectiveness of PTs, in order to provide standard and reliable data that can be used to inform future PT designs. Most of the studies reviewed presented subjective data with no standard approach by which to measure whether or not the technologies were effective, and to what extent they were effective.
2. **Using Behavior Theories to Inform Persuasive Technology Design**: Although our analysis could not successfully compare the effectiveness of PTs employing behavior theories and those that did not, due to the limited number of studies employing theory. Our result shows that although limited, PTs employing theory in their design tend to be more effective than those based not on any theory, although marginal. This supports previous research suggesting that PTs based on theory are more effective than those based on intuition [206–208]. A possible reason why most PT designers do not employ theories is probably because most designers lack the necessary background to appropriately interpret behavior theories and translate them into actionable and practical PT design components [208]. Hence, PT designers can collaborate with people that have an adequate background such as behavioral scientists to achieve this. Therefore, *we recommend that PT designer employ behavior change theories in their design and clearly state how the theoretical components were translated into the design components in the PTs*.
3. **Effectiveness of Persuasive Technologies Employing Multiple Strategies vs. Those Based on a Single Strategy**: There is also a need to establish the effectiveness of PTs employing a single persuasive strategy in comparison to those employing multiple strategies. Although, employing multiple strategies has been the convention in the area with the hope that the more the better. However, this may not be the case. As shown by Orji et al. [209], PTs employing a single strategy can be effective. Nevertheless, it is unknown whether employing multiple strategies would result to more effective PTs; that is if the strategies have an additive effects. We also acknowledge that employing multiple strategies may lead to a cognitive overload on the part of the users. Hence, *we recommend that future research should focus on establishing the effectiveness of PTs employing a single strategy in comparison to those employing multiple strategies and also how this may vary depending on how the strategies are implemented*.
4. **Effectiveness of Persuasive Strategies Across Contexts**: Although the review focused on studies in the area of PA and SB, we also noticed a variation in the choice of strategies which are majorly and randomly chosen due to the lack of clear guideline on which strategy works under various contexts. Hence, *we recommend that the effectiveness of the strategies be evaluated across domains and technologies to establish domain or technology-dependent factors that may impact effectiveness*. That is, does the effectiveness of the strategies depend on the technology platform and/or the domain of application or they generalized? Research in this area would identify the strengths and weaknesses of each strategy based on many factors, including the sample demographics, their health conditions, and the target behavior. This is essential for advancing the field and contributing to the design of future PT.
5. **Mix-Method Approach to Persuasive Technology Evaluation**: Researchers should employ mix methods approach to uncover the full effects of their PTs. Most existing studies employed the quantitative approach, and this is good as it allows for tracking of the actual PA behavior; however, it gives no insight into the process through which PTs motivated users and inspire the observed behavior change. Qualitative methods such as interviews, on the other hand, would allow users to express their feeling and the motives behind their actions. This would give insight into the reasons behind their actions, which would, in turn, shed more light on the mechanism through which PTs promotes behavior change. Hence, *we recommend that designers should employ a combination of quantitative and qualitative approaches (mixed methods) when evaluating the effectiveness of their PTs*.
6. **Longitudinal Evaluation of Persuasive Technology Effectiveness:** More than half of the reviewed studies 104 (61%) conducted their assessment in duration between <1 week and 1-to-3 months, whereas only 20 (12%) of studies conducted longitudinal evaluations between 4 months and 2 years. Therefore, there is a need to conduct more long-term evaluations to establish the effectiveness and users' adherence to PTs over the long-term for a sustained behavior change in the area of PA and SB domains.
7. **Accessible Cross-platform Persuasive Technologies**: A good number of evaluated PTs are multi-platforms PT intervention. They are implemented to run across multiple technology platforms such as a combination of smartphones, activity trackers devices, cameras, and height-adjustable workstations. We cannot state categorically that it contributes to the effectiveness of such interventions, however, it appears to be a good practice only considering that implementing cross-platform PTs increases the accessibility of such PTs, the reach, and makes them always available for users owning multiple technologies. Therefore, *we recommend that PT designers consider designing a cross-platform application to increase their reach and accessibility*.
8. **Comprehensive Persuasive Technology Design Framework**: Existing PT design models and frameworks are not comprehensive to guide the analysis of current PTs. We identified some strategies that are not captured in the popular PSD model. This is possibly due to advancements in technology evolution, which have made many strategies that would not have been imagined a decade ago possible. Therefore, *we suggest that more work is needed in the area of developing a comprehensive PT design framework that identified not only the strategies but also various possible implementation, domain, user group, technology, and other contextual factors that may affect their effectiveness*. This will hence, facilitate tailoring of PTs based on may contextual factors and user type. The PSD model was useful in organizing the strategies, but it was not enough to include all the resulted strategies. Furthermore, we sometimes faced some confusion when using the PSD model strategies because of the similarity between some of its strategies as well as the method of implementing such strategies in the design of PT based on a designer's own intuition. For example, the growth levels and the happy facial expression of the fish in the “Fish in Steps” system can be considered feedback and rewards strategies, whereas a sad or angry facial expression can also be classified as punishments (or negative reinforcements as they are known), reminders, and feedback strategies [39].
  Again, in most cases, we had to study the functionality for most of the strategies in-depth, which many did not specify clearly, requiring extra time and effort to identify them from the articles. They also had different names and classifications, which made it even more difficult to identify and code them into the PSD model.
9. **Unified Standard for Target Audience Categorization**: The classifications of the demographics by their age groups are sometimes unclear. For example, the age group of adults was varied in the reviewed articles, and this is the same with other age groups such as teenagers and children. This may cause considerable confusion when classifying the targeted audience by their age group. Therefore, *we suggest a unified standard for age group categorization*.
10. **Publication Biases**: It is important to consider publication bias and how it may have affected the present review. This means that papers with positive or significant results are more likely to be submitted and published compared to those with negative findings. However, future research may benefit from research that has reported negative findings/complications with the use of PT. Such information may be useful in directing the design of future PT.
11. **Diversification of the Target Audience of Persuasive Technology**: Most of the reviewed studies were targeted at adults, therefore it is necessary to develop more PT systems that target different populations, such as children, teenagers, and the elderly.
12. **Clarity of the Persuasive Technology Design Objectives**: It is clear that there is confusion regarding the PA and SB domains. People may misunderstand the difference between these domains because they might consider that the most common purpose of designing the PT in such fields is very often the same goal when aiming to reduce the time the user spends sedentarily and to increase his/her PA levels. However, it is important for researchers to distinguish between the terminologies of PA and SB. This is because each domain may require PT designers to employ different persuasive strategies or implement the strategies differently to achieve the desired objectives based on the targeted domain—PA, SB, or both. For instance, PT aimed at motivating users to achieve the Moderate Intensity Physical Activity (MIPA) level (e.g., 30 min of moderate intensity physical activity (MIPA) daily or 150 min of MIPA weekly) may be different from that aimed at motivating users to perform periodic movements (e.g., standing, stretching, walking) every 30 min or every 1 h to avoid a sedentary lifestyle.

Table 15 displays a list of some essential for PT researchers and designers. This alongside the 12 recommendations above could be used to inform future design and analysis of PT for PA and SB.

**Table 15:** Check list for future design and research of PT for PA and/or SB.

Tailoring PTs targeted population	Researchers have to consciously consider the targeted population by their age demographics, health conditions, jobs, and their general status in designing PT and employing appropriate and suitable persuasive strategies. PTs that are tailored and reflect the target audience realities tend to be more effective than the generic ones that employ the one-size-fits-all approach.
Design approach	To effectively tailor and consider the target audience in PT designs, PT designers should employ the iterative user-centered design approaches which involve studying and engaging the target audience from the onset of the design to the final deployment and evaluation.
Privacy	• It is essential to provide users with their performance feedback, notifications, and progress updates without intruding on their autonomy or privacy. • Users need to have control over which and how data will be tracked and what they will be used for.
Duration of evaluation	Researchers need to conduct longitudinal evaluations for their PT design to assess the effectiveness and users' commitment and adherence in continuing to use the PT over the long term in the area of PA and SB.
PT platforms	Although most existing studies employed multiple technology platforms (e.g., a combination of a wearable activity tracker and mobile phone) in PT design to persuade users to perform more PA and reduce SB, this may be burdensome on the user and discourage long-term use as they may not be seamlessly integrated into user's daily. We suggest that simple PTs based on a single platform that can easily integrate into user's daily lives should be preferred over complex ones that requires combining and carrying many gadgets.
Evaluation approach	Designers should prefer mix-method evaluation that combines both quantitative and qualitative approaches over a single method. This tend to provide a comprehensive evaluation of the PTs, uncovering not only what works but why and how they work
Behavior theories	Studies employing theory in their design tend to be more effective than those based not on any theory. Therefore, we recommend that PT designers employ behavior change theories in their design and clearly state how the theoretical components were translated into the design components in the PTs.
Others	• Researchers need to state specifically the main purpose of their PT design, whether aiming to increase PA alone, reduce SB alone or both. • Researchers need to state clearly the persuasive strategies they employed and how such strategies are implemented in their PT design (e.g., a self-monitoring strategy that was employed as graphical/visual feedback on a smartphone screen). • Researchers need to consider employing one or a set of PT design models and frameworks to guide the analysis of PTs and the persuasive strategies employed.

### 5.8. Notes for Future Design of Persuasive Technology for PA and SB

An important point to note is that many of the reviewed studies implemented their PT in more than one technology platforms, such as a combination of smartphones, wearable activity trackers' devices, smartwatch, and sensory chairs, therefore each of these can be considered a multi-platform intervention to achieve the main objective of a study to increase PA levels and reduce SB. This seems to be common considering that users tend to own multiple gadgets these days and to ensure that the PT is always available, they may need to be cross-platform, e.g., integrated with both smartwatch and mobile phone. In that way, it presents multiple opportunities to persuade and motivate users. More importantly, it can be used by users owning various technology, technology-independent. However, this means that the overall cost of implementing PT would increase. Users often do not want to be limited by the technology platform. Hence PT designer, especially those targeting PA and SB, should be aware of this.

Another essential point to consider is that most of the PT employed two or more persuasive strategies (e.g., tunneling, self-monitoring, rewards, reminders, expertise, and social comparison) to persuade users to be physically active and to make them more aware of the side effects of being sedentary. This makes it impossible to know which of the employed strategies resulted in the observed behavior change.

Again, it is also essential that PT designers explicitly state the main objective and purpose of their design, whether targeting in increasing PA alone or decreasing SB alone or both. Most times, this is not clear and a reader would have to deduce from the working of the system, study design, and measured evaluation outcome. This makes analyzing existing studies difficult to achieve.

It is important to state that there is a tiny difference between encouragement and persuasion on one side and coercion and deception on another ([210, 211]). Therefore, it is essential to consider this variation in general when designing PTs, and for health and wellness in particular such as PA and SB domains. According to Vlieghe and De Troyer ([211]), there are some ethical considerations of persuasion that need to be considered:

- The app needs to be tailored to the users' needs and deliver feedback, notifications, progress updates, and cues, which if not carefully implemented, may be considered as surveillance. There is a need to balance between the collection of data and the intruding on the autonomy and privacy of the user.
- There is also a need to design PTs that permit the user to control how the data is tracked, and what it is used for. This is important for all PTs but more important for PTs that track some health data and health-related behavior data.
- The technologies have to be designed in a way that the persuasive design do not lower the users' autonomy.

## 6. Conclusion and Future Work

The paper provides a detailed systematic review of 170 paper to establish the effectiveness PTs for promoting health and wellness in the domains of Physical Activity and Sedentary Behavior. Our findings show that almost three quarters [137 studies (80%)] of the total reviewed studies (170 studies) reported successful outcomes, whether fully or partially successful, which means that PTs are effective at promoting (PA) and discouraging (SB). Thus, the findings demonstrate that the use of PT has the potential to promote desirable behavior change among the users when combined with the proper persuasive strategy. Furthermore, the study summarizes and highlights trends in the outcomes including system design, research methods, persuasive strategies and implementations, behavioral theories, and employed technological platforms. The most frequently targeted populations are adults and young adults, while the least are older people, children, teenagers, and young children. The outcomes of this work illustrate that the most two effective and commonly employed technology platforms in the field of PA and/or SB are mobile and handheld devices, and activity trackers and sensors (whether commercially available or custom-designed by researchers).

Furthermore, this study shows that the most effective and frequently implemented persuasive strategies in PT design for promoting PA and/or reducing SB are tracking/self-monitoring, reminders, personalization, goal setting, rewards, and the set of social support strategies, in decreasing order. Our results show that, although limited, the studies employing behavioral theories in their design tend to be more effective and promising than those not based on any theory. In addition, the research shows that applying the mixed evaluation method (a combination of quantitative and qualitative approaches) is more useful to uncover the full effect of PTs. Finally, we identified the pitfalls and gaps in the present literature that could be leveraged and used to inform the design of a PT that targets PA. Accordingly, we provide a list of general limitations and recommendations to advance and improve future research.

Future works may need to evaluate studies done in the field of PTs in promoting PA and SB according to the different targeted populations by age demographics (e.g., older people, teenagers, children). Future works should also conduct more long-term evaluations to establish the effectiveness of and users' adherence to the PT over the long term in the area of PA and SB. Additionally, we suggest analyzing PTs based on each of technology platforms used in their design. Finally, we also recommend evaluating users' reviews/feedback for the existing PTs (e.g., applications, systems, or devices) to advance the future design of PTs for PA and SB.

## Data Availability Statement

All datasets generated for this study are included in the article/Supplementary Material.

## Author Contributions

NA conducted the paper search, the thematic analysis, and wrote the first version of the manuscript. FA and RO contributed to reviewing and refining the manuscript. RO and SS supervised the study.

## Conflict of Interest

The authors declare that the research was conducted in the absence of any commercial or financial relationships that could be construed as a potential conflict of interest.
