# Supplementary material for: Trends in Persuasive Technologies for Physical Activity and Sedentary Behavior: A Systematic Review
Source: Front Artif Intell. 2020 Apr 28;3:7. doi: 10.3389/frai.2020.00007 (PMC7861265; doi:10.3389/frai.2020.00007)
Supplement: Supplementary file 1 [file Table_1.docx]

APPENDIX

**Appendix 1. A Comprehensive Overview of Persuasive Technology for Physical Activity and/or Sedentary Behavior**

| **#** | **Authors of Articles, Year, Reference** | **Domain** | **Technology** | **Application / Project Name** | **Persuasive Strategies /Affordances** | **Theories** | **Evaluation Method** | **Duration** | **Targeted Outcomes** | **Audience Age Group** | **No. of Participants** | **Effectiveness / Evaluation Outcomes** | **Country of Study** |
| --- | --- | --- | --- | --- | --- | --- | --- | --- | --- | --- | --- | --- | --- |
| 1 | Grosinger et al.(2012), [69] | PA | Tablet computer (PC) | Agile Life | Tracking, Tailoring, Goal Setting, Self-Monitoring ( Graphical (Images) Feedback), Praise, Rewards, Reminders, Suggestion, Trustworthiness, Expertise, Social support (Social Facilitation, Social Cooperation) | none | Qualitative | 1 Week | Behavior, Awareness, Motivation | Elderly | 7 | Partially Successful | Austria |
| 2 | Esakia et al.(2018, [45]) | PA | Smartwatch, Mobile app, Website | FitAware | Tracking, Goal Setting, Self-Monitoring (Feedback displayed as non-interruptive passive notifications), Social Support (Cooperation, Competition) | none | Mixed | 2 Months | Behavior, Awareness | Adults | 24 | Fully Successful | USA |
| 3 | Gouveia et a.(2015), [67] | PA | Smartphone mobile, Wearable activity trackers | Habito | Tracking, Reduction, Goal Setting, Self-Monitoring, Praise (Textual Feedback), Rewards, Suggestion (Textual Feedback), Surface Credibility | TTM, GST | Quantitative | 10 Months | Behavior, Users' Engagements | Adults | 265 | Partially Successful | Portugal |
| 4 | Gao et al.(2014), [59] | SB | Computer Windows (PC), Game, Sensors | GrabApple Game | Tunneling, Simulation, Rewards, Suggestions | none | Mixed | 5 Months | Behavior, Attitude | Children | 60 | Fully Successful | Canada |

**Appendix 1. (continued)**

| **#** | **Authors of Articles, Year, Reference** | **Domain** | **Technology** | **Application / Project Name** | **Persuasive Strategies /Affordances** | **Theories** | **Evaluation Method** | **Duration** | **Targeted Outcomes** | **Audience Age Group** | **No. of Participants** | **Effectiveness / Evaluation Outcomes** | **Country of Study** |
| --- | --- | --- | --- | --- | --- | --- | --- | --- | --- | --- | --- | --- | --- |
| 5 | Foster et al.(2010), [54] | PA | Smartphone mobile, Computer, Pedometer | StepMatron | Tracking, Personalization, Goal Setting, Self-Monitoring (Textual Feedback Notifications), Social Support (Social Learning, Comparisons, Competition, Rankings Recognition, Giving Comments) | none | Mixed | 3 Weeks | Behavior | Adults | 10 | Fully Successful | UK |
| 6 | He and Agu (2014), [83] | SB | Smartphone mobile | On11 | Tracking, Reduction, Tunneling, Tailoring, Personalization, Goal Setting, Self-Monitoring (Self-Reflection), Reminder (Sound Notification or Vibrating), Suggestion, Liking | none | Qualitative | 2 Weeks | Behavior, Awareness | Adults | 8 | Partially Successful | USA |
| 7 | Fahim et al.(2017) , [48] | SB | Smartphone mobile | Alert Me | Tracking, Personalization, Self-Monitoring (Visual Feedback), Reminder and Textual Feedback (Personalized Messages that suggest short breaks) | none | Quantitative | Unspecified | Behavior, Awareness | Unspecified | 0 | Fully Successful | Russia |
| 8 | Mohadis and Ali (2016), [116] | PA | Smartphone mobile | WargaFit | Tracking, Reduction, Tunneling, Tailoring, Personalization, Self-Monitoring, Simulation, Rehearsal, Praise, Reminders, Suggestions, Similarity, Expertise, Real world feel, Third-Party Endorsement, Verifiability, Social Support (Social Learning, Social Comparison, Normative influence, Social facilitation, Competition, Recognition) | none | Mixed | Unspecified | Behavior | Elderly | 8 | Fully Successful | Malaysia |

**Appendix 1. (continued)**

| **#** | **Authors of Articles, Year, Reference** | **Domain** | **Technology** | **Application / Project Name** | **Persuasive Strategies /Affordances** | **Theories** | **Evaluation Method** | **Duration** | **Targeted Outcomes** | **Audience Age Group** | **No. of Participants** | **Effectiveness / Evaluation Outcomes** | **Country of Study** |
| --- | --- | --- | --- | --- | --- | --- | --- | --- | --- | --- | --- | --- | --- |
| 9 | Ayubi and Parmanto (2012), [10] | PA | Smartphone mobile, Facebook, Pedometer application | PersonA | Tracking, Reduction, Self-Management (Self-Monitoring, Self-Measurement, Self-Comparison, Goal Setting), Praise, Positive Reinforcement, Self-Monitoring (Real-Time Feedbacks and Visual Representation & Aural Feedback), Rewards, Reminders, Surface Credibility (Security Settings and Privacy/Confidentiality Settings), Social Support (Comparison, Cooperation, Competition, Sharing Experiences, Posting Feeds, Giving Comments) | TPB, SCT | none | none | Behavior, Adherence | Unspecified | 0 | none | USA |
| 10 | Luo et al.(2018), [106] | SB | Desktop-based system (PC) | Time for Break | Personalization, Feedback from users (Self-Report when responding to the reminders), Reminder | none | Mixed | 3 Weeks | Behaviors, Intentions | Adults | 25 | Partially Successful | USA |
| 11 | Pellegrini et al.(2015), [136] | PA, and SB | Smartphone mobile application , Wireless accelerometers | NEAT! | Tracking, Reduction, Reminder (Noise or Vibration Prompt) | none | Quantitative | 1 Month | Behavior, Awareness | Adults | 8 | Partially Successful | USA |
| 12 | Cambo et al.(2017), [25] | SB | Smartphone mobile, Smartwatch | BreakSense | Tracking, Personalization, Self-Monitoring, Rewards, Reminder | none | Mixed | 8 Days | Behavior | Adults | 6 | Partially Successful | USA |

**Appendix 1. (continued)**

| **#** | **Authors of Articles, Year, Reference** | **Domain** | **Technology** | **Application / Project Name** | **Persuasive Strategies /Affordances** | **Theories** | **Evaluation Method** | **Duration** | **Targeted Outcomes** | **Audience Age Group** | **No. of Participants** | **Effectiveness / Evaluation Outcomes** | **Country of Study** |
| --- | --- | --- | --- | --- | --- | --- | --- | --- | --- | --- | --- | --- | --- |
| 13 | Zuckerman and Gal-Oz (2014), [169] | PA | Smartphone mobile | StepByStep | V1 (Quantified): Tracking, Self-Monitoring (Real-Time Feedback, Visual Feedback), Praise, Goal Settings. V2 (Gamification): As same as version 1 plus Virtual Rewards. V3 (Gamification): All features of V1 plus Social Comparison. | none | Mixed | Study1: 2 Weeks. Study 2: 10 Days. | Behavior, Motivation | Adults | 90 | Partially Successful | Israel |
| 14 | Al-Hrathi and Consolvo (2012), [1] | PA | Exergaming system, Sensors | ExerLearn Bike System | Personalization, Simulation, Liking (Attractive System by Text, Audio, and Visual Representation) | none | Mixed | Unspecified | Cognitive, Physical Abilities | Children | 8 | Fully Successful | Canada |
| 15 | Munson et al.(2012), [120] | PA | Smartphone mobile application, Facebook | GoalPost and GoalLine | Tracking, Goal-Setting, Self-Monitoring, Rewards, Reminder, Suggestions, Surface Credibility (Privacy), Social Support (Sharing) | TTM, GST | Qualitative | 1 Month | Behavior | Adults | 23 | Partially Successful | USA |
| 16 | Mansart et al.(2015), [108] | PA and SB | Smartphone-based mobile exergame | Go Run Go | Tracking, Tunneling, Personalization, Self-Monitoring, Rewards, Social Support (Sharing) | none | Mixed | Unspecified | Behavior | Adults | 10 | Fully Successful | Thailand |
| 17 | Chen and Pu (2014), [30] | PA | Smartphone mobile game, Wearable activity tracker (Fitbit) | HealthyTogether | Tracking, Self-Monitoring, Rewards, Social Support (Cooperation or Competition or Hybrid (Social Interaction), Messages Exchange) | none | Mixed | 2 Weeks | Behavior, Awareness | Adults | 36 | Fully Successful | Switzerland |

**Appendix 1. (continued)**

| **#** | **Authors of Articles, Year, Reference** | **Domain** | **Technology** | **Application / Project Name** | **Persuasive Strategies /Affordances** | **Theories** | **Evaluation Method** | **Duration** | **Targeted Outcomes** | **Audience Age Group** | **No. of Participants** | **Effectiveness / Evaluation Outcomes** | **Country of Study** |
| --- | --- | --- | --- | --- | --- | --- | --- | --- | --- | --- | --- | --- | --- |
| 18 | Fortmann et al.(2013), [53] | SB | Ambient light display, Smartphone mobile, Laptop, Pedometer application | MoveLamp | Tracking, Self-Monitoring (Visual & Numerical Feedback), Reminder | TDDS | Quantitative | 2 Days | Behavior, Awareness, Motivation | Adults | 10 | Fully Successful | Germany |
| 19 | Lin et al.(2011), [105] | PA | Smartphone mobile application, web application | Motivate | Tracking, Reduction, Personalization, Feedback from users (Self-Report), Reminder, Suggestion | none | Mixed | 5 Weeks | Behavior, Awareness | Adults | 6 | Fully Successful | Netherlands |
| 20 | Dharia et al.(2016), [40] | PA | Smartphone mobile applications | PRO-Fit | Tracking, Reduction, Personalization, Self-Monitoring (Graphical Feedback), Reminder, Suggestion, Authorization, Social Support | none | none | none | Behavior | Unspecified | 0 | none | USA |
| 21 | Nair et al.(2016), [122] | Health Care, and SB | Smartwatch app, Web, Sensors | ROAMM | Tracking, Personalization, Self-Monitoring (Visual Feedback), Feedback from users (Self-Report), Reminder, Surface Credibility (Secure Server) | none | Quantitative | Unspecified | Behavior, Awareness | Adults | 10 | Fully Successful | USA |
| 22 | Klein et al.(2017), [97] | PA | Smartphone mobile application, Web page, Facebook, Wearable activity tracker (Fitbit) | Active2Gether | Tracking, Reduction, Tailoring, Personalization, Goal Setting, Self-Monitoring (Textual Feedback), Simulation, Reminder, Suggestion, Liking, Social Role, Surface Credibility, Social Support (Social Comparison) | MBR, TTM, DCM, SCT, SRT, HAPA | Qualitative | 3 Months | Behavior, Awareness | Young Adults | 100 | Fully Successful | Netherlands |

**Appendix 1. (continued)**

| **#** | **Authors of Articles, Year, Reference** | **Domain** | **Technology** | **Application / Project Name** | **Persuasive Strategies /Affordances** | **Theories** | **Evaluation Method** | **Duration** | **Targeted Outcomes** | **Audience Age Group** | **No. of Participants** | **Effectiveness / Evaluation Outcomes** | **Country of Study** |
| --- | --- | --- | --- | --- | --- | --- | --- | --- | --- | --- | --- | --- | --- |
| 23 | Khalil and Abdallah(2013), [94] | PA | Smartphone mobile application | STEP UP | Tracking, Self-Monitoring (Display History Feedback), Social Support (Competition, Sharing) | TRA | Mixed | 2 weeks | Behavior, Attitudes, motivation | Adults | 8 | Fully Successful | United Arab Emirates (UAE) |
| 24 | Arteaga et al.(2010), [9] | PA | Smartphone mobile game application | Mobile App | Tracking, Reduction, Personalization, Self-Monitoring, Reward, Social Support (Competition) | TPB, TMB, PT | Qualitative | 1 Month | Behavior Motivation | Teenagers | 5 | Fully Successful | USA |
| 25 | Chen et al.(2014), [29] | PA | Smartphone mobile, game, Sensor, Camera | SP-Stretch | Tracking, Reduction, Reward, Social Support (Social Competition) | SCT | Mixed | 1 Month | Motivation | Adults | 25 | Fully Successful | Taiwan |
| 26 | Lim et al.(2011), [103] | PA | Shoe physical activity tracker, Pedometer | Pediluma | Tracking, Personalization, Self-Monitoring (Visual Feedback), Reward, Reminder, Social Support (Social Learning (Social Pressure (Public Display), Sharing) | TTM | Mixed | 2 Weeks | Behavior, Attitude, Awareness, Motivation | Adults | 18 | Fully Successful | USA |
| 27 | Luca Chittaro and Riccardo Sioni (2012), [31] | PA | Smartphone mobile exergame | LocoSnake game | Tracking, Reduction, Personalization, Self-monition (Real-Time Feedback, Vibration Feedback), Simulation, Rewards | none | Mixed | 5 Minutes | Attitude | Young Adults | 15 | Fully Successful | Italy |

**Appendix 1. (continued)**

| **#** | **Authors of Articles, Year, Reference** | **Domain** | **Technology** | **Application / Project Name** | **Persuasive Strategies /Affordances** | **Theories** | **Evaluation Method** | **Duration** | **Targeted Outcomes** | **Audience Age Group** | **No. of Participants** | **Effectiveness / Evaluation Outcomes** | **Country of Study** |
| --- | --- | --- | --- | --- | --- | --- | --- | --- | --- | --- | --- | --- | --- |
| 28 | Young (2010), [166] | PA | Micro-blogging site (Twitter), Cell-phone, Website | Twitter-like micro-blogging site | Tracking, Personalization, Goal Setting, Self-Monitoring (Text Messages Textual Feedback), Self-Report, Praise, Rewards, Social Support (Cooperation, Competition, Sharing, Sending Encouraging Feedback) | none | Quantitative | 1 Month | Behavior | Teenagers | 4 | Fully Successful | USA |
| 29 | Zwinderman et al.(2012), [170] | PA | Smartphone mobile game, Smartphone mobile application, Website | Phone Row | Tracking, Personalization, Self-monitoring (Visual Feedback), Simulation, Praise, Reward, Liking, Surface Credibility (Security), Social Support (Social Comparison, Competition, Social Recognition (Leaderboard Rank), Sharing) | none | Qualitative | 4 Days | Behavior | Adults | 32 | Unsuccessful | Netherlands |
| 30 | Harjumaa and Oinas-Kukkonen (2009), [81] | PA | Heart rate monitor | Polar FT60 | Tracking, Reduction, Tailoring, Personalization, Goal Setting, Self-Monitoring (Summary Feedback), Praise (Verbal Feedback), Rewards, Reminders, Social Role, Trustworthiness, Expertise, Surface Credibility | none | Qualitative | 3 Months | Behavior | Young Adults | 12 | Unspecified | Finland |
| 31 | Dantzig et al.(2013), [38] | PA, SB, computer activity (CA) | Smartphone mobile application, Activity monitor technology | SitCoach | SitCoach Experiment: Goal Setting, Self-Monitoring (Timely Feedback), Reminder (Visual, Acoustic, and Tactile Messages), Social Support (Social Comparison, Normative Influence, Sharing) Sedentary Break Experiment: Reminder, Praise | none | Quantitative | 6 Weeks | Behavior, Awareness, Motivation | Adults | 94 | Partially Successful | Netherlands |

**Appendix 1. (continued)**

| **#** | **Authors of Articles, Year, Reference** | **Domain** | **Technology** | **Application / Project Name** | **Persuasive Strategies /Affordances** | **Theories** | **Evaluation Method** | **Duration** | **Targeted Outcomes** | **Audience Age Group** | **No. of Participants** | **Effectiveness / Evaluation Outcomes** | **Country of Study** |
| --- | --- | --- | --- | --- | --- | --- | --- | --- | --- | --- | --- | --- | --- |
| 32 | Munguia and Santana (2010), [119] | PA and SB | Web service, Mobile, Computer, a Wearable augmented reality system (Pedometer, camera, accelerometer) | youWalk | Tracking, Rewards, Social Support (Cooperation, Social Interaction, Sharing, Text Message (Invitation)) | none | none | none | Behavior, Motivation | Adults | 10 | none | Mexico |
| 33 | Probst et al.(2012), [139] | PA and SB | Integrated activity-promoting office workspace environment | Active Office | Tailoring, Personalization, Simulation | none | none | none | Behavior | Unspecified | 0 | none | Austria |
| 34 | Fialho et al.(2009), [52] | PA | Web service application, Accelerometer | ActiveShare | Tracking, Self-Monitoring (Graphical Feedback), Reminder, Expertise (Virtual Coach is a Real Expert), Social Support (Social Goal Setting, Sharing Activities, Set/Accept Challenges, Message, Tag) | none | Quantitative | 17 Days | Behavior, Motivation | Adults | 12 | Partially Successful | Netherlands |
| 35 | Berkovsky et al.(2010), [13] | PA | Computer, Wearable activity monitoring devices | A wearable activity interface and Neverball game | Tracking, Reductions, Self-monitoring (feedback), Rewards | none | Quantitative | 20 Minutes | Behavior, Motivation | Children | 180 | Fully Successful | Australia |
| 36 | Grundgeiger et al.(2017), [70] | SB | Smartphone mobile application | Mobile App | Tracking, Personalization, Reminder | none | Quantitative | 5 Days | Behavior Awareness | Adults | 5 | Partially Successful | Germany |

**Appendix 1. (continued)**

| **#** | **Authors of Articles, Year, Reference** | **Domain** | **Technology** | **Application / Project Name** | **Persuasive Strategies /Affordances** | **Theories** | **Evaluation Method** | **Duration** | **Targeted Outcomes** | **Audience Age Group** | **No. of Participants** | **Effectiveness / Evaluation Outcomes** | **Country of Study** |
| --- | --- | --- | --- | --- | --- | --- | --- | --- | --- | --- | --- | --- | --- |
| 37 | Keung et al.(2013), [93] | PA | Smartphones mobile phone, MIT App Inventor | BunnyBolt | Tracking, Tunneling, Rewards, Reminder, Surface Credibility | none | none | none | Behavior | Young Adults | 0 | none | USA |
| 38 | Ornelas et al.(2015), [129] | PA | Smartphone mobile application | CrowdWalk | Tracking, Reduction (Map Viewing Visualization of Nearby Activities and Challenges), Tunneling, Goal Setting (Walking Challenge), Suggestion | none | Mixed | Unspecified | Behavior, Awareness | Adults | 65 | Fully Successful | Portugal |
| 39 | Hirano et al.(2013), [85] | PA | Smartphone mobile application | WalkMinder | Tracking, Goal Setting, Self-Monitoring (Visual Feedback), Reminders | none | Qualitative | 1 Month | Behavior, Awareness | Adults | 8 | Partially Successful | USA |
| 40 | Chatta et al.(2015), [28] | SB | Computer Game | Sonic All Stars Transformed | Tracking and Monitoring, Simulation (Flashing Visual Feedback), Praise (Textual Feedback), Social Support (Competition) | none | Mixed | 45 - 60 Minutes | Behavior, Motivation | Young Adults | 34 | Partially Successful | USA |
| 41 | Penados et al.(2010), [101] | PA | A cuddly toy with electronic interaction capacities, Accelerometer, RFID technology | Gum | Tunneling, Simulation, Rewards, Reminder (Audio Feedback), Suggestion, Social Support (Competition, Social Interaction) | none | Mixed | 6 Months | Behavior | Young Children | 19 | Fully Successful | Netherlands |
| 42 | Yitong Huang (2016), [87] | SB | Digitally “enchanted” office objects , Sensors | Enchanted Objects (EOs) | Tracking, Self-Monitoring (Self-Reflection, Goal-Monitoring, Feedback), Reminder | TDF | Qualitative | Unspecified | Behavior, Feasibility, Acceptability | Adults | 20 | Fully Successful | UK |

**Appendix 1. (continued)**

| **#** | **Authors of Articles, Year, Reference** | **Domain** | **Technology** | **Application / Project Name** | **Persuasive Strategies /Affordances** | **Theories** | **Evaluation Method** | **Duration** | **Targeted Outcomes** | **Audience Age Group** | **No. of Participants** | **Effectiveness / Evaluation Outcomes** | **Country of Study** |
| --- | --- | --- | --- | --- | --- | --- | --- | --- | --- | --- | --- | --- | --- |
| 43 | Zaman et al.(2014), [167] | PA and SB | Computer, Smartphone mobile, Sensors | K-Sense monitoring system | Tracking | none | Quantitative | 90 Minutes | Behavior | Adults | 34 | Fully Successful | USA |
| 44 | Bieber et al.(2010), [16] | PA | Smartphone mobile, Accelerometer | Mobile phone app | Tracking | none | Quantitative | Unspecified | Behavior | Adults | 12 | Fully Successful | Germany |
| 45 | Min et al.(2015), [114] | SB | Smartphone mobile applications, Sensor-Equipped seat device (BackJoy) | Pretty Pelvis | Tracking, Self-Monitoring (Summary and Graphical Feedback), Simulation, Reminder (Sound Notification), Suggestion | none | Quantitative | Unspecified | Behavior, Awareness | Adults | 11 | Unspecified | South Korea |
| 46 | Probst et al.(2013), [138] | SB | Interactive sensing chair | Interactive sensing chair | Tracking, Tunneling, Simulation, Reminder | none | none | none | Behavior, Motivation | Adults | 0 | none | Austria |
| 47 | Schagen et al.(2015), [149] | SB | Smartphone mobile application (A virtual aquarium) | Super Starfish Mania | Tracking, Self-Monitoring, Rewards, Reminder, Social Support (Cooperation, Competition, Recognition, Collaboration, Sharing) | none | none | none | Behavior, Awareness, Motivation | Unspecified | 0 | none | Netherlands |
| 48 | Marinac et al.(2013), [110] | SB | Computer, Wearable Camera (SenseCam) | Unspecified | Tracking and Monitoring, Surface Credibility | none | Quantitative | 94 Days | Behavior | Adults | 28 | Partially Successful | USA |

**Appendix 1. (continued)**

| **#** | **Authors of Articles, Year, Reference** | **Domain** | **Technology** | **Application / Project Name** | **Persuasive Strategies /Affordances** | **Theories** | **Evaluation Method** | **Duration** | **Targeted Outcomes** | **Audience Age Group** | **No. of Participants** | **Effectiveness / Evaluation Outcomes** | **Country of Study** |
| --- | --- | --- | --- | --- | --- | --- | --- | --- | --- | --- | --- | --- | --- |
| 49 | Cercos and Mueller (2013), [27] | PA | Computer, Shared semi-public display, Wearable activity tracker (Fitbit) | Watch your Steps | Tracking, Self-Monitoring (Graphical Feedback), Social Support (Competition), Tunneling (Fictional Player To Guide a user to proceed to the Goal) | SDT, SCT, TTM | Quantitative | 2 Months | Behavior, Awareness | Adults | 15 | Fully Successful | Australia |
| 50 | Dobbins et al.(2014), [42] | SB | Web system, Accelerometer, Heart rate monitor sensors, Camera, Location detection | DigMem memory box | Tracking and Monitoring, Self-Monitoring (Visual Feedback) | none | Quantitative | none | Behavior | Unspecified | 0 | Fully Successful | UK |
| 51 | Moghimi et al.(2014, [115] | SB | Computer, ImageNet 2012 classifier, Wearable camera (SenseCam) | Unspecified | Tracking and Monitoring | none | Quantitative | 3 - 5 Days | Behavior | Adults ( | 40 | Partially Successful | USA |
| 52 | Kumar et al.(2016), [98] | SB | Computer, Chair with sensors | Care-Chair | Tracking | none | Quantitative | 33 Minutes 16 Seconds | Behavior | Unspecified | 5 | Fully Successful | USA |
| 53 | Gupta and Sood (2015), [75] | PA, and Stress, and Obesity | Smartphone application (Android), Web services | Let’s Exercise | Tracking, Self-Monitoring, Reminder, Suggestion | none | Quantitative | 1 Month | Behavior, Motivation | Young Adults | 33 | Fully Successful | USA |
| 54 | Synnott et al.(2016), [158] | SB | Thermal sensors | Thermal sensor | Tracking and Monitoring | none | Quantitative | 3 Days | Behavior | Adults | 0 | Fully Successful | Ireland |

**Appendix 1. (continued)**

| **#** | **Authors of Articles, Year, Reference** | **Domain** | **Technology** | **Application / Project Name** | **Persuasive Strategies /Affordances** | **Theories** | **Evaluation Method** | **Duration** | **Targeted Outcomes** | **Audience Age Group** | **No. of Participants** | **Effectiveness / Evaluation Outcomes** | **Country of Study** |
| --- | --- | --- | --- | --- | --- | --- | --- | --- | --- | --- | --- | --- | --- |
| 55 | Pina et al.(2012), [137] | SB | Computer, Wearable activity tracker (Fitbit) | Fitbit+ | Tracking, Self-Monitoring, Praise, Reminder as a (Positive or Negative Reinforcement), Suggestion | none | none | none | Behavior, Awareness | Adults | 30 | none | USA |
| 56 | Almkerk et al.(2015), [4] | SB and Sitting Posture (SP) | Smartphone application, Interactive office chair (Backtive), Sensors | Backtive | Tracking, Reminder (Tactile and Visual Feedback), Expertise, Surface Credibility | UTAUT | Quantitative | Unspecified | Behavior, Awareness | Adults | 4 | Fully Successful | Belgium |
| 57 | Haque et al.(2016), [80] | PA | Mobile to web application (Android) | iGO | Personalization, Self-Monitoring, Rewards, Reminder (Alarm "Sound" and Vibration Feedback), Social Support (Competition, Recognition) | SDT | Qualitative | 1 Week | Behavior | Young Adults | 26 | Fully Successful | Finland |
| 58 | Bond et al.(2014), [19] | SB | Smartphone application, Wearable sensor | B-MOBILE | Tracking, Personalization, Goal Setting, Self-Monitoring (Reinforcement Feedback), Praise, Rewards, Reminder (Reinforcement Feedback) | none | Quantitative | 16 Months | Behavior, Motivation | Adults | 30 | Fully Successful | USA |
| 59 | Renfree and Cox (2016), [144] | SB | User Interfaces (ambient displays) with LEDs, Seat sensors | Tangible User Interfaces (TUIs) | Tracking, Self-Monitoring (Ambient Display Feedback (LED Color and Intensity Visualization)), Rewards | TPB | none | none | Behavior, Awareness | Adults | 0 | none | UK |

**Appendix 1. (continued)**

| **#** | **Authors of Articles, Year, Reference** | **Domain** | **Technology** | **Application / Project Name** | **Persuasive Strategies /Affordances** | **Theories** | **Evaluation Method** | **Duration** | **Targeted Outcomes** | **Audience Age Group** | **No. of Participants** | **Effectiveness / Evaluation Outcomes** | **Country of Study** |
| --- | --- | --- | --- | --- | --- | --- | --- | --- | --- | --- | --- | --- | --- |
| 60 | Dobbins et al.(2012), [41] | SB | Lifelogging process by using activPAL accelerometer, SenseWear Armband (SWA) | Unspecified | Tracking, Self-Monitoring (Visual Lifelogs) | none | none | none | Behavior | Elderly | 0 | none | UK |
| 61 | Dewa et al.(2012), [39] | SB and Mental Health | Computer, Pedometer | none | Tracking | none | Quantitative | 1 Month | Behavior | Adults | 28 | Partially Successful | Canada |
| 62 | Barwais et al.(2013), [11] | SB and PA | Web personal activity monitor-based intervention program, Accelerometer, LED | Gruve Solution | Tracking, Goal Setting, Self-Monitoring (Visual Feedback "Graphs, Charts", LED Colors Visualization (Green means Goal Achievement)), Praise (Encouraging Textual Feedback), Reminder | none | Quantitative | 1 Month | Behavior | Adults | 33 | Fully Successful | Australia |
| 63 | Mueller et al.(2009), [118] | PA | Exertion games, Videoconferencing, Videotaping, Camera | Table Tennis for Three | Simulation, Social Support (Communicating via Video-Conferencing "Video Streams, Microphone", Competition) | GT | Qualitative | 50 - 90 Minutes | Behavior | Adults | 39 | Fully Successful | Australia |
| 64 | Gilson et al.(2013), [64] | PA | Website program, Pedometer | Walk@Work | Tracking , Self-Monitoring (Visual Feedback), Reminder, Suggestions | none | Quantitative | 3 Months | Behavior | Adults | 330 | Partially Successful | Australia, Canada, Northern Ireland, and USA |

**Appendix 1. (continued)**

| **#** | **Authors of Articles, Year, Reference** | **Domain** | **Technology** | **Application / Project Name** | **Persuasive Strategies /Affordances** | **Theories** | **Evaluation Method** | **Duration** | **Targeted Outcomes** | **Audience Age Group** | **No. of Participants** | **Effectiveness / Evaluation Outcomes** | **Country of Study** |
| --- | --- | --- | --- | --- | --- | --- | --- | --- | --- | --- | --- | --- | --- |
| 65 | Jung et al.(2009), [90] | PA, and Psychological and Physical Well-Being | Video-Game technology | Nintendo Wii | Simulation, Social Support (Competition) | SPF | Quantitative | 6 Weeks | Behavior, Emotion, Self-Esteem, Loneliness, Balance | Elderly | 45 | Fully Successful | Singapore |
| 66 | Fan et al.(2012), [49] | PA | Web application, Wearable activity tracker (Fitbit), Tablet visualization | Spark | Tracking, Reduction, Self-Monitoring (Visual & Graphical Feedback), Rewards | TDDS | Qualitative | 3 Weeks | Behavior, Awareness | Adults | 6 | Fully Successful | USA |
| 67 | Jafarinaimi et al.(2004), [88] | SB | Ambient sculpture display, Chair sensors | Breakaway | Tracking, Self-monitoring, Simulation, Reminder (Vibrotactile Feedback) | none | Mixed | 2 Weeks | Behavior, Awareness | Adults | 1 | Fully Successful | USA |
| 68 | Rodr ́ıguez et al.(2013), [145] | PA | A mobile ambient information system | CAMMInA | Tailoring, Goal Setting, Self-Monitoring (History Feedback), Rewards (Positive Reinforcement), Reminder (Audible and Textual Feedback), Suggestion, Social Support | CDT | Mixed | 1 day | Behavior, Awareness, Motivation | Elderly | 15 | Partially Successful | Mexico |
| 69 | Braun et al.(2015), [21] | SB | Desktop training software (PC), Microbreak training application, a Sensor-equipped smart chair | ExerSeat | Tracking, Reminder | none | Mixed | 2 Months | Behavior | Elderly | 10 | Partially Successful | Finland |

**Appendix 1. (continued)**

| **#** | **Authors of Articles, Year, Reference** | **Domain** | **Technology** | **Application / Project Name** | **Persuasive Strategies /Affordances** | **Theories** | **Evaluation Method** | **Duration** | **Targeted Outcomes** | **Audience Age Group** | **No. of Participants** | **Effectiveness / Evaluation Outcomes** | **Country of Study** |
| --- | --- | --- | --- | --- | --- | --- | --- | --- | --- | --- | --- | --- | --- |
| 70 | Park et al.(2013), [132] | PA | Computer, Smartphone mobile, Application-program interface (API), smart shoes, 3D Accelerometers | ExerSync | Tracking, Liking (Audio-Visual Rhythmic Representation), Social Support | TS | Mixed | Unspecified | Behavior | Young Adults | 74 | Unsuccessful | South Korea |
| 71 | Mueller et al.(2003), [117] | PA | Exertion Interfaces, Video-conference, Microphones, Camera | Breakout for Two | Tracking and Monitoring, Simulation, Social Support (Competition) | none | Mixed | Unspecified | Behavior | Adults | 56 | Fully Successful | Ireland, USA |
| 72 | Guo et al.(2017), [73] | PA | Virtual fitness coach, Wearable mobile devices and sensors, Smartphones | FitCoach | Tracking, Tunneling, Self-Monitoring (Performance Feedback), Suggestion | none | Quantitative | 6 Months | Behavior | Adults | 12 | Fully Successful | USA |
| 73 | Skriloff et al.(2016), [150] | PA | Smartphone mobile application (Android), Wearable activity tracker (Fitbit) | FitPlay Games platform | Tracking, Personalization, Social Support (Cooperation, Competition) | none | none | none | Behavior, Motivation | Unspecified | 0 | none | USA |
| 74 | McMahon et al.(2013), [112] | PA | Smartphone mobile application | Ready~Steady | Reduction, Personalization, Goal Setting, Self-Monitoring (Visual Feedback), Simulation, Praise, Rewards, Social Role | WMT, USS, TDP | none | none | Behavior | Elderly | 0 | none | USA |
| 75 | Buddharaju and Locanathan (2016), [23] | PA | Smartphone mobile, ExerPad | Mobile Exergame | Tracking, Simulation | none | Quantitative | Unspecified | Behavior | Young Adults | 20 | Fully Successful | USA |

**Appendix 1. (continued)**

| **#** | **Authors of Articles, Year, Reference** | **Domain** | **Technology** | **Application / Project Name** | **Persuasive Strategies /Affordances** | **Theories** | **Evaluation Method** | **Duration** | **Targeted Outcomes** | **Audience Age Group** | **No. of Participants** | **Effectiveness / Evaluation Outcomes** | **Country of Study** |
| --- | --- | --- | --- | --- | --- | --- | --- | --- | --- | --- | --- | --- | --- |
| 76 | Ramanathan et al.(2012), [142] | PA and Experience Sampling | Mobile to web platform | ohmage | Tracking, Personalization, Self-Monitoring (Graphical "Visual" Feedback), Feedback from users (Self-Report), Praise, Surface Credibility (Secure Server), Social Support | none | none | none | Behavior | Unspecified | 0 | none | USA |
| 77 | Stanley et al.(2010), [155] | PA and Obesity | Smartphone mobile game application | PiNiZoRo | Tracking, Reduction, Personalization, Simulation | none | Qualitative | Unspecified | Behavior, Awareness | Children | 4 | Fully Successful | Canada |
| 78 | Bexheti et al.(2015), [15] | PA | Smartphone mobile, Wearable mobile sensors, Wearable Camera | Re-Live app | Tracking, Self-Monitoring (Visual Feedback), Surface Credibility (Secure Server) | CPT | Qualitative | 10 Days | Behavior, Attitude, Awareness | Adults | 10 | Partially Successful | Italy |
| 79 | Hao et al.(2015), [79] | PA | Smartphone mobile application, Bluetooth headset, Accelerometer, Microphone | RunBuddy | Tracking, Self-Monitoring (Real-Time Visual Feedback), Suggestion, Surface Credibility (Privacy) | LRC | Quantitative | Unspecified | Behavior | Adults | 13 | Fully Successful | USA |

**Appendix 1. (continued)**

| **#** | **Authors of Articles, Year, Reference** | **Domain** | **Technology** | **Application / Project Name** | **Persuasive Strategies /Affordances** | **Theories** | **Evaluation Method** | **Duration** | **Targeted Outcomes** | **Audience Age Group** | **No. of Participants** | **Effectiveness / Evaluation Outcomes** | **Country of Study** |
| --- | --- | --- | --- | --- | --- | --- | --- | --- | --- | --- | --- | --- | --- |
| 80 | Anderson et al.(2007), [7] | PA | Smartphone mobile, Artificial Neural Network (ANN) | Shakra | Tracking, Personalization, Self-Monitoring (Real-Time Feedback), Social Support (Comparison, Competition, Sharing) | HMM, TTM, SCT | Mixed | 10 Days | Behavior, Awareness, Motivation | Adults | 9 | Partially Successful | UK |
| 81 | De Oliveira and Oliver (2008), [127] | PA | Mobile phone, Sensors | TripleBeat | Tracking, Reduction (Predefined Workout Plans), Tunneling (Real-Time Musical Feedback), Self-Monitoring (Visual and Textual Feedback), Suggestion, Social Support (Competition) | none | Quantitative | Unspecified | Behavior, Awareness | Young Adults | 10 | Fully Successful | Brazil |
| 82 | Macvean and Robertson (2013), [107] | PA | Smartphone mobile | iFitQuest | Tracking, Reduction, Tunneling, Goal Setting, Simulation, Rewards | TSE | Mixed | 7 Weeks | Behavior | Children | 12 | Partially Successful | UK |
| 83 | Hamiliton et al.(2012), [78] | SB and Obesity | Smartphone mobile application, Web, Facebook | Walk2Build | Tracking, Goal Setting, Self-Monitoring (Visual Feedback), Simulation, Rewards, Social Support (Comparison, Sharing) | TTM, SCT, SET, SP | none | none | Behavior, Awareness | Teenagers and Young Adults | 0 | none | UK |
| 84 | Guida et al.(2017), [72] | PA | Smartphone mobile, Bluetooth, Web-dashboard, Sensors | WeightBit | Tracking, Goal Setting, Self-Monitoring (Visual Feedback), Rewards, Reminder, Surface Credibility, Third-Party Endorsement, Social Support (Sharing) | none | none | none | Behavior, Motivation | Unspecified | 0 | none | UK |

**Appendix 1. (continued)**

| **#** | **Authors of Articles, Year, Reference** | **Domain** | **Technology** | **Application / Project Name** | **Persuasive Strategies /Affordances** | **Theories** | **Evaluation Method** | **Duration** | **Targeted Outcomes** | **Audience Age Group** | **No. of Participants** | **Effectiveness / Evaluation Outcomes** | **Country of Study** |
| --- | --- | --- | --- | --- | --- | --- | --- | --- | --- | --- | --- | --- | --- |
| 85 | Gui et al.(2017), [71] | PA | Smartphone mobile application | WeRun | Tracking, Self-Monitoring, Social Support (Social Learning, Cooperation, Recognition (Ranking), Sharing, Sending Liking, Follow) | GT | Qualitative | 4 Months | Behavior, Awareness | Adults | 32 | Partially Successful | China |
| 86 | Peeters et al.( 2013), [135] | PA | Intelligent musical staircase, Sound actuators, Sensors, Camera | Social Stairs | Rewards | none | Qualitative | 3 Weeks | Behavior, Motivation | Unspecified | 0 | Fully Successful | Netherlands |
| 87 | Fujinami and Reikki (2008), [58] | PA | Ambient Mirror Display, Pedometer | Ambient display | Tracking, Goal Setting, Self-Monitoring (History Feedback), Suggestion, Social Support (Competition, Collaboration, Sharing, Chatting) | none | Quantitative | 1 Week | Motivation, Awareness | Adults | 6 | Fully Successful | Japan |
| 88 | Eyck et al.(2006), [47] | PA | Virtual Coach, Heartbeat Sensor | Virtual coach cycling machine | Tracking, Verbal Feedback (Tunneling (Virtual Coach), Tailoring, Praise) | none | Quantitative | 15 Minutes | Motivation | Young Adults | 20 | Fully Successful | Netherlands |
| 89 | Toscos et al.(2008), [162] | PA | Mobile phone application, Pedometer | Mobile App | Tracking, Personalization, Goal Setting, Self-Monitoring, Praise (Persuasive Textual Feedback), Reminder, Social Support (Comparison, Competition, Sharing) | none | Mixed | 3 Weeks | Behavior | Teenagers | 8 | Partially Successful | USA |
| 90 | Albaina et al.(2009), [2] | PA | Virtual coach, Context-aware technology, Pedometer | Flowie (Virtual coach) | Tracking, Tunneling (Virtual Coach), Goal Setting, Self-Monitoring (Visual Feedback), Rewards | CLT | Mixed | 11 Days | Behavior, Motivation | Elderly | 34 | Partially Successful | Netherlands |

**Appendix 1. (continued)**

| **#** | **Authors of Articles, Year, Reference** | **Domain** | **Technology** | **Application / Project Name** | **Persuasive Strategies /Affordances** | **Theories** | **Evaluation Method** | **Duration** | **Targeted Outcomes** | **Audience Age Group** | **No. of Participants** | **Effectiveness / Evaluation Outcomes** | **Country of Study** |
| --- | --- | --- | --- | --- | --- | --- | --- | --- | --- | --- | --- | --- | --- |
| 91 | Sakai et al.(2011), [146] | PA | Publicly displayed screen, Bluetooth, mobile phone | APStairs | Personalization, Reminder, Authority | none | Quantitative | 5 Weeks | Behavior | Adults | 28 | Partially Successful | Netherlands |
| 92 | Mutsuddi and Connelly (2012), [121] | PA | Mobile text messaging app, Pedometer | Mobile phone text messaging app | Tailoring, Personlization, Goal Setting, Self-Monitoring (Textual Message Feedback), Praise, Reward, Reminder, Suggestion, Social Support (Sharing) | TTM | Mixed | 3 Months | Behavior | Young Adults | 30 | Fully Successful | USA |
| 93 | Fritz et al.(2014), [56] | PA | Wearable Activity tracker Devices | Unspecified | Tracking, Goal Setting, Reward, Social Support (Social-Sharing) | none | Qualitative | 25 – 45 Minutes | Behavior | Adults | 58 | Fully Successful | North America, Europe, Asia |
| 94 | Lacroix et al.(2009), [99] | PA | Wearable activity-tracker device | Unspecified | Tracking, Self-Monitoring (Visual Feedback) | SDT | Quantitative | 10 Days | Behavior, Motivation, Self-efficacy | Adults | 7 | Fully Successful | Netherlands |
| 95 | Toscos et al.(2006), [161] | Eating and PA | Cell-phone application, Pedometer | Chick Clique | Tracking, Reduction, Personalization, Self-Monitoring (Textual Feedback), Praise, Social Support (Cooperation, Competition, Sharing) | none | Mixed | 6 Days | Behavior, Awareness | Female Teenagers | 10 | Fully Successful | USA |
| 96 | Fujiki et al.(2008), [57] | PA | Cell-phone Mobile Game, Wearable accelerometer | NEAT-o-Games (NEAT-o-Race, NEAT-o-Sudoku) | Tracking, Self-Monitoring (Real-Time Text Feedback, Activity History), Simulation, Praise, Social Support (Competition) | none | Mixed | 1 Month | Behavior | Young Adults | 10 | Fully Successful | USA |

**Appendix 1. (continued)**

| **#** | **Authors of Articles, Year, Reference** | **Domain** | **Technology** | **Application / Project Name** | **Persuasive Strategies /Affordances** | **Theories** | **Evaluation Method** | **Duration** | **Targeted Outcomes** | **Audience Age Group** | **No. of Participants** | **Effectiveness / Evaluation Outcomes** | **Country of Study** |
| --- | --- | --- | --- | --- | --- | --- | --- | --- | --- | --- | --- | --- | --- |
| 97 | Lin et al.(2006), [104] | PA and SB | Computer game (Animated virtual fish), Pedometers | Fish'n'Steps | Tracking, Goal Setting, Self-Monitoring (Visual Growth), Simulation, Rewards ( Positive Reinforcements (Emotional Feedback)), Punishments (Negative Reinforcements (Emotional Feedback)), Social Support (Cooperation, Competition, Sharing), Gamification (Game) | TTM | Mixed | 14 Weeks | Behavior, Attitude, Awareness | Adults | 19 | Fully Successful | USA |
| 98 | Consolvo and Klasnja (2008), [36] | PA | Mobile phones, Activity sensor | UbiFit Garden | Tracking, Goal Setting, Self-Monitoring (Visual Feedback), Praise, Rewards, Reminder | TTM | Mixed | 3 Months | Behavior, Motivation | Young Adults | 28 | Fully Successful | USA |
| 99 | Berkovsky et al. (2012), [14] | PA | Computer Game, Pedometer, Accelerometer | PLAY MATE! | Tracking, Reward, Liking (Feedback Interaction between the Game and the Player) | OCT, TPP | Quantitative | Unspecified | Behavior, Motivation | Children | 225 | Fully Successful | Australia |
| 100 | McCreadie et al. (2006), [111] | PA | Mobile application, Personal Digital Assistants (PDAs) | Personal Navigation Tool | Tracking | none | Qualitative | 30 - 45 Minutes | Unspecified | Elderly | 5 | Unspecified | UK |
| 101 | Clinkenbeard et al. (2014), [34] | PA | Social Network (Personal Facebook networks) | Social network app | Reduction, Tunneling, Suggestion | none | Quantitative | 3 Days | Attitude | Adults | 61 | Fully Successful | USA |

**Appendix 1. (continued)**

| **#** | **Authors of Articles, Year, Reference** | **Domain** | **Technology** | **Application / Project Name** | **Persuasive Strategies /Affordances** | **Theories** | **Evaluation Method** | **Duration** | **Targeted Outcomes** | **Audience Age Group** | **No. of Participants** | **Effectiveness / Evaluation Outcomes** | **Country of Study** |
| --- | --- | --- | --- | --- | --- | --- | --- | --- | --- | --- | --- | --- | --- |
| 102 | Nakajima and Lehdonvirta (2013), [123] | PA | Ambient Mirror system | Persuasive Art | Tracking, Personalization, Self-Monitoring (Visual Feedback), Simulation, Reward (Positive Reinforcements (Expressions)), Punishments (Negative Reinforcements (Expressions)), Reminder | GST, OCT | Mixed | 31 Days | Behavior | Young Adults | 14 | Unsuccessful | Japan |
| 103 | Park et al.(2012), [133] | PA | Social exergames, Aerobic (RIA) exercise devices, Mobile devices (smartphones, tablets), Mobile Internet Devices (MIDs) | ExerLink | Tracking, Reduction, Tunneling, Personalization, Self-Monitoring (Summary Feedback), Social Support (Competition, Collaboration, Voice Social Interaction) | none | Mixed | Unspecified | Behavior | Young Adults | 36 | Fully Successful | South Korea |
| 104 | Kim et al.(2016), [96] | SB and Sitting Posture (SP) | Computer (Windows OS), Visual feedback system, Camera, Sensors | Visual feedback system | Tracking and Monitoring, Reminder (Pop-up Notification, Visual Feedback) | none | Quantitative | 1 Hour | Behavior | Young Adults | 14 | Fully Successful | South Korea |
| 105 | Mattias Wolfel(2017), [165] | SB and SP | Computer (Windows), Sensor, Camera, Projector | Ambient display | Tracking, Self-Monitoring (Visual Feedback), Simulation, Reminder | none | Quantitative | 3 Hours | Behavior | Adults | 16 | Fully Successful | Germany |
| 106 | Ferreira et al.(2014), [51] | SB and SP and Stress | Computer desktop system, Web camera, Open CV platform | BreakOut | Tracking and Monitoring, Simulation, Reminder (Real-Time and Ambient Feedback), Suggestion | none | Quantitative | 4 Days | Behavior | Adults | 10 | Partially Successful | Portugal |

**Appendix 1. (continued)**

| **#** | **Authors of Articles, Year, Reference** | **Domain** | **Technology** | **Application / Project Name** | **Persuasive Strategies /Affordances** | **Theories** | **Evaluation Method** | **Duration** | **Targeted Outcomes** | **Audience Age Group** | **No. of Participants** | **Effectiveness / Evaluation Outcomes** | **Country of Study** |
| --- | --- | --- | --- | --- | --- | --- | --- | --- | --- | --- | --- | --- | --- |
| 107 | Ren et al.(2016), [143] | SB and SP | A portable smart exercise pillow, Tablet or Smartphone or Computer, Sensors | Flow Pillow | Tracking, Simulation, Reminder (Auditory "Musical" Feedback), Social Support | FT | Mixed | 30 Minutes | Behavior | Elderly | 5 | Partially Successful | Netherlands |
| 108 | Obermair et al (2008), [126] | SB and SP | Interactive picture frame | perFrame | Rewards or Punishments (Effective Picture Feedback (Positive or Negative "Emotion" Reinforcement)) | none | Qualitative | Unspecified | Awareness | Adults | 8 | Fully Successful | Austria |
| 109 | Karime et al.(2012), [91] | PA and Mental Health | Exergames (video games), Sensory-mounted tiles (pads) | MeMaPads | Personalization, Simulation | none | Mixed - | 1 Month | Behavior, Cognitive | Young Children | 6 | Fully Successful | Canada |
| 110 | Sohn and Lee (2007), [151] | PA and Smoking | PDA text messaging, Instant Messaging (IM) system, Mobile device | UP Health | Tracking, Personalization, Goal Setting, Self-Monitoring, Reward or Punishment, Reminder (Textual Feedback), Social Support (Cooperation, Competition) | none | Qualitative | 1 week | Behavior, Awareness | Adults | 5 | Partially Successful | South Korea |
| 111 | Kientz et al. (2010), [95] | Eating and PA | Mindbloom: Website game, Sensors MyPyramid Blast Off: Computer Game, sensors | Mindbloom, and MyPyramid Blast Off | Mindbloom: Tracking, Goal Setting, Self-Monitoring (Visual Feedback), Simulation, Rewards, Social Support (Sharing). MyPyramid Blast Off: Tracking, Reduction, Tunneling, Self-Monitoring (Visual Feedback), Praise, Real-World Feel | none | Mixed | 15 Minutes | Behavior | Children | 10 | Fully Successful | USA |

**Appendix 1. (continued)**

| **#** | **Authors of Articles, Year, Reference** | **Domain** | **Technology** | **Application / Project Name** | **Persuasive Strategies /Affordances** | **Theories** | **Evaluation Method** | **Duration** | **Targeted Outcomes** | **Audience Age Group** | **No. of Participants** | **Effectiveness / Evaluation Outcomes** | **Country of Study** |
| --- | --- | --- | --- | --- | --- | --- | --- | --- | --- | --- | --- | --- | --- |
| 112 | Gasca et al. (2008), [60] | Eating and PA | Mobile, Web, Pedometer | pHealthNet | Tracking, Self-monitoring, Praise (Encouraging Textual Feedback), Social Support (Collaboration, Challenge Notification and Goal Setting Issued from Friends) | none | Mixed | 1 Month | Behavior, Engagement | Elderly | 6 | Fully Successful | Mexico |
| 113 | Gasser et al. (2006), [61] | Eating and PA | Smartphone Mobile, Website | Mobile App | Goal Setting, Self-Monitoring, Social Facilitation Smartphone Interface: Goal Setting, Self-Monitoring (Visual Feedback), Reminder, Social Support (Social Learning, Messages). Web Interface: The same but in a Single Screen | none | Quantitative | 28 Days | Usage, Acceptance | Adults | 40 | Fully Successful | Switzerland |
| 114 | Gerber et al. (2009), [62] | Eating and PA | Mobile phone | Mobile text messaging app | Textual Feedback (Tailoring, Reminder, Suggestion) | none | Mixed | 4 Months | Attitude | Adults | 95 | Fully Successful | USA |
| 115 | Sun et al.(2014), [157] | Health and Wellbeing (Diet, PA, SB, Elderly, Blind) | Wearable computer, Sensors, Camera | eButton application | Tracking and Monitoring, Tunneling, Personalization | none | none | none | Behavior | Unspecified | 0 | none | USA |

**Appendix 1. (continued)**

| **#** | **Authors of Articles, Year, Reference** | **Domain** | **Technology** | **Application / Project Name** | **Persuasive Strategies /Affordances** | **Theories** | **Evaluation Method** | **Duration** | **Targeted Outcomes** | **Audience Age Group** | **No. of Participants** | **Effectiveness / Evaluation Outcomes** | **Country of Study** |
| --- | --- | --- | --- | --- | --- | --- | --- | --- | --- | --- | --- | --- | --- |
| 116 | Donath et al.(2015), [43] | SB | Computer PC prompt with text, Height-adjustable work desks (HAWD), Wearable activity tracker (ActiGraph) | HAWD | Tracking, Tailoring, Personalization, Simulation, Reminders | none | Quantitative | 1 Year | Behavior | Adults | 31 | Fully Successful | Switzerland |
| 117 | Gilson et al.(2016), [65] | SB | Real time computer prompts, Chair sensor/software package, Wearable accelerometer (GENEActiv) | Sitting Pad | Tracking, Self-monitoring, Reminder (Real Time and Reactive Feedback) | none | Quantitative | 5 Months | Behavior | Adults | 57 | Fully Successful | Australia |
| 118 | Puig-Ribera et al.(2015), [141] | SB | Web-based intervention (W@WS), Pedometer | Walk@WorkSpain, W@WS | Tracking, Tailoring, Goal Setting, Self-Monitoring, Praise (Encouraging Textual Feedback), Suggestion, Social Support (Sharing) | none | Quantitative | 22 Weeks | Behavior | Adults | 190 | Fully Successful | Spain |
| 119 | Taylor et al.(2016), [160] | PA and SB | Computer PC prompt | Booster Break and Computer-prompt | Reminder | none | Quantitative, | 6 Months | Behavior | Adults | 175 | Partially Successful | USA |

**Appendix 1. (continued)**

| **#** | **Authors of Articles, Year, Reference** | **Domain** | **Technology** | **Application / Project Name** | **Persuasive Strategies /Affordances** | **Theories** | **Evaluation Method** | **Duration** | **Targeted Outcomes** | **Audience Age Group** | **No. of Participants** | **Effectiveness / Evaluation Outcomes** | **Country of Study** |
| --- | --- | --- | --- | --- | --- | --- | --- | --- | --- | --- | --- | --- | --- |
| 120 | Carr et al.(2013), [26] | SB | StepWatch activity tracker device, Portable pedal machine including activity tracking and monitoring software, Pedometer | Unspecified | Tracking, Self-Monitoring (Performance Feedback) | none | Quantitative | 3 Months | Behavior | Adults | 40 | Fully Successful | USA |
| 121 | Pronk et al.(2012), [140] | SB | Cellular telephone, a Sit-stand workstation | Take-a-Stand | Tracking/Monitoring, Tailoring, Personalization, Reminder (Text Messages), Simulation | none | Quantitative | 7 Weeks | Behavior | Adults | 34 | Fully Successful | USA |
| 122 | Hadgraft et al.(2017), [77] | SB | A Sit-stand workstation | Stand up Victoria | Tailoring, Personalization, Simulation | none | Qualitative | 1 Year | Behavior, Perspective | Adults | 28 | Fully Successful | Australia |
| 123 | Spinney et al.(2015), [153] | PA and SB | OpenBeacon active RFID, ActivPAL system for accelerometer information | Active Buildings | Tracking | none | Quantitative | 1 Week | Behavior | Adults | 33 | Partially Successful | UK |
| 124 | Glynn et al.(2013), [66] | PA | smartphone, Pedometer | Accupedo | Tracking, Personalization, Goal Setting, Self-Monitoring (Graphical and Performance Feedback), Social Support (Sharing) | none | Qualitative | 2 Months | Behavior | Adults | 80 | Unspecified | Ireland |

**Appendix 1. (continued)**

| **#** | **Authors of Articles, Year, Reference** | **Domain** | **Technology** | **Application / Project Name** | **Persuasive Strategies /Affordances** | **Theories** | **Evaluation Method** | **Duration** | **Targeted Outcomes** | **Audience Age Group** | **No. of Participants** | **Effectiveness / Evaluation Outcomes** | **Country of Study** |
| --- | --- | --- | --- | --- | --- | --- | --- | --- | --- | --- | --- | --- | --- |
| 125 | Neuhaus et al.(2014), [125] | SB | Height-adjustable workstation, ActivPAL3 monitor | Height-Adjustable workstations | Tailoring, Personalization, Goal Setting, Self-Monitoring (Normative Feedback), Simulation, Reminder, Suggestion | SCT | Quantitative | 6 Months | Behavior, Reducing Sitting Time | Adults | 44 | Fully Successful | Australia |
| 126 | Parry et al.(2013), [134] | SB and PA | Computer, Actical accelerometer | Unspecified | Tracking | none | Quantitative | 1 Week | Behavior | Adults | 50 | Fully Successful | Australia |
| 127 | Jago et al.(2013), [89] | PA and Screen-Viewing (SV) | Accelerometer (Active Pal) | Teamplay program | Tracking | none | Mixed | 16 Weeks | Behavior | Children | 48 | Fully Successful | UK |
| 128 | SPRUIJT-METZ et al.(2008), [154] | PA and SB | A Classroom Animation intervention | Get Moving! | Tailoring, Self-Report, Praise (Textual Messages) | SDT, TMB | Quantitative | 3 Months | Motivation | Teenagers | 459 | Fully Successful | USA |
| 129 | Brakenridge et al.(2016), [20] | SB | Smartphone mobile application, Activity trackers and monitors (LUMOback) (ActivPAL3) | Stand Up Lendlease program | Tracking, Self-Monitoring (Real-Time Feedback and Prompts on Sitting and Posture), Reminders | none | Quantitative | 1 Year | Behavior | Adults | 153 | Fully Successful | Australia |
| 130 | De Cocker et al.(2016), [35] | SB | Computer, Web, Activity monitor (activPAL) | Unspecified | Tracking, Tailoring, Personalization, Self-Monitoring (Personal Feedback), Suggestion, Expertise | TDDS, SDT, TPB, SRT | Quantitative | 3 Months | Behavior | Adults | 93 | Fully Successful | Belgium |

**Appendix 1. (continued)**

| **#** | **Authors of Articles, Year, Reference** | **Domain** | **Technology** | **Application / Project Name** | **Persuasive Strategies /Affordances** | **Theories** | **Evaluation Method** | **Duration** | **Targeted Outcomes** | **Audience Age Group** | **No. of Participants** | **Effectiveness / Evaluation Outcomes** | **Country of Study** |
| --- | --- | --- | --- | --- | --- | --- | --- | --- | --- | --- | --- | --- | --- |
| 131 | Evans et al.(2012), [46] | SB | Computer PC prompt with text, Activity monitor (ActivPAL) | Point-of-choice (PoC) | Tracking, Tailoring, Reminder | none | Quantitative | 1 Year | Behavior | Adults | 28 | Fully Successful | UK |
| 132 | Urda et al.(2016), [163] | SB | Computer, Activity monitor (ActivPAL3) | Unspecified | Tracking, Reminder (Audible Alert) | none | Quantitative | 2 Weeks | Behavior | Adults | 44 | Unsuccessful | USA |
| 133 | Otten et al.(2009), [130] | SB and Watching TV | TV, & Electronic monitors, Sensors SenseWear Pro 3 | Electronic lock-out system for the TV | Tracking and Monitoring | none | Quantitative | 6 Weeks | Behavior | Adults | 36 | Partially Successful | USA |
| 134 | Hadgraft et al.(2016), [76] | Sedentary Behavior (SB) | Activity monitors (ActivPAL3), Wearable activity tracker (Actigraph) | Unspecified | Tracking | none | Quantitative | 2 Years | Behavior | Adults | 231 | Unspecified | Australia |
| 135 | Healy et al.(2013), [84] | Sedentary Behavior (SB) | Activity monitors (ActivPAL3 ), sit–stand workstations | Unspecified | Tracking, Tailoring, Personalization, simulation | none | Quantitative | 1 Month | Behavior | Adults | 43 | Unspecified | Australia |
| 136 | Alkhajah et al.(2012), [3] | SB | Sit–stand workstation, Activity monitor (ActivPAL3 ) | Unspecified | Tracking, Tailoring, Personalization, simulation | none | Quantitative | 3 Months | Behavior | Adults | 32 | Fully Successful | Australia |
| 137 | Altmeyer et al.(2018), [5] | PA | Augmented portable bike and persuasive see-through mirror | SilverCycling | Tracking, Self-Monitoring, Praise, Reward, Social Support (Comparison, Normative Influence, Cooperation, Competition) | none | Quantitative | Unspecified | Behavior, Motivation | Elderly | 9 | Partially Successful | Germany |

**Appendix 1. (continued)**

| **#** | **Authors of Articles, Year, Reference** | **Domain** | **Technology** | **Application / Project Name** | **Persuasive Strategies /Affordances** | **Theories** | **Evaluation Method** | **Duration** | **Targeted Outcomes** | **Audience Age Group** | **No. of Participants** | **Effectiveness / Evaluation Outcomes** | **Country of Study** |
| --- | --- | --- | --- | --- | --- | --- | --- | --- | --- | --- | --- | --- | --- |
| 138 | Burkow et al. (2018), [24] | PA | Tablet-based Computer Application | Unspecified | Tracking, Reduction, Tunneling, Personalization, Goal-Setting, Self-Monitoring, Rewards, Social Support ( competition) | Unspecified | Mixed | 6 Weeks | Behavior, Acceptance, Adherence | Elderly | 10 | Partially Successful | Norway |
| 139 | Gupta et al.(2018), [74] | PA, SB, Sleep | A web application and Fitbit tracker | FitViz | Tracking, Personalization, Goal Setting, Self-Monitoring, Reminder, Social Role, Expertise | none | Qualitative | 1 month | Behavior, Awareness, self-management | Elderly | 20 | Partially Successful | Canada |
| 140 | Marcu et al. (2018), [109] | PA | Smartphone mobile | Bounce | Reduction, Tunneling, Personalization, Goal Setting, Self-Monitoring, Praise, Rewards, Reminders, Social Role, Trustworthiness, Expertise, Authority, Social Support (Normative Influence, Cooperation, Social Interaction) | TTM, SCT | Qualitative | 3 Weeks | Behavior, Attitude, Awareness, Motivation | Adults | 4 | Fully Successful | USA |
| 141 | Meyer et al.(2018), [113] | PA | Ambient sensors and monitors of stair climbing | ActiStrairs | Tracking, Tunneling, Self-Monitoring, Praise, Rewards, Social Support ( Cooperation) | none | Mixed | 3 Days | Behavior, Attitude, Acceptance, Awareness, Motivation | General population ( 20 - above 80 years old) | 358 | Partially Successful | Germany |
| 142 | Zhang and Jemmott (2019), [168] | PA | Mobile application, Activity Tracker (Fitbit) | PennFit | Tracking, Personalization, Self-Monitoring, Reminder, Social Support ( Comparison, Social Interaction ( messages with chatting tool)) | SCT | Quantitative | 3 Months | Behavior, Awareness | Young Adults | 91 | Fully Successful | USA |

**Appendix 1. (continued)**

| **#** | **Authors of Articles, Year, Reference** | **Domain** | **Technology** | **Application / Project Name** | **Persuasive Strategies /Affordances** | **Theories** | **Evaluation Method** | **Duration** | **Targeted Outcomes** | **Audience Age Group** | **No. of Participants** | **Effectiveness / Evaluation Outcomes** | **Country of Study** |
| --- | --- | --- | --- | --- | --- | --- | --- | --- | --- | --- | --- | --- | --- |
| 143 | Lee et al. (2018), [102] | PA and SB | Smartphone application | Puzzle Walk | Tunneling, Personalization, Goal Setting, Self-Monitoring, Praise, Rewards, Reminder, Liking | Unspecified | none | none | Behavior, Motivation | Adults | 34 | none | USA |
| 144 | Buckers et al. (2018), [22] | PA | Video-Game technology via augmented reality (AR) | VRabl | Reduction, Simulation, Goal Setting (from the game), Rewards, Liking, Social Support (Competition) | none | Quantitative | Unspecified | Motivation | Young Adults | 15 | Partially Successful | Netherlands |
| 145 | Clavel et al.(2018), [33] | PA | Smartwatch, phone , website | WEnner | Wenner: Tailoring, Personalization. WennerStep : Tailoring, Personalization, Goal, Praise, Reminder. WennerAgent: Tunneling, Self-Monitoring, Praise | RFT | Qualitative | 1 Week | Behavior, Motivation | Unspecified | 30 | Partially Successful | France |
| 146 | Gouveia et al.(2018), [68] | PA | A wearable cameras and Fitbit Tracker | in vivo | Tracking, Personalization, Goal Setting, Self-Monitoring, Reminder | GST | Mixed | 2 Days | Behavior, Motivation | Adults | 12 | Partially Successful | Portugal |
| 147 | Stragier et al.( 2015), [156] | PA | Wearable activity-tracker devices, and smartphone, Website Social Networking Sites ( Facebook and Twitter) | Strava | Tracking, Goal Setting, Self-Monitoring, Social Support (Comparison, Social Interaction, Social Sharing) | SDT | Quantitative | Unspecified | Motivation | Adults | 400 | Partially Successful | Belgium |

**Appendix 1. (continued)**

| **#** | **Authors of Articles, Year, Reference** | **Domain** | **Technology** | **Application / Project Name** | **Persuasive Strategies /Affordances** | **Theories** | **Evaluation Method** | **Duration** | **Targeted Outcomes** | **Audience Age Group** | **No. of Participants** | **Effectiveness / Evaluation Outcomes** | **Country of Study** |
| --- | --- | --- | --- | --- | --- | --- | --- | --- | --- | --- | --- | --- | --- |
| 148 | Harrington et al.(2018), [82] | PA | Mobile fitness applications , SNS | 3 Mobile Fitness applications (Endomondo, Burn'emDown, StepFit) | Endomondo: Social support (SNS).  Brun'emDown: Goal Setting.  StepFit: Tracking | none | Qualitative | 10 Weeks | Acceptance, Adherence | Elderly | 25 | Fully Successful | USA |
| 149 | Nakamura et al.(2005), [124] | (PA) | Web browser system, and wearable activity sensors | EnergyBrowser | Tracking, Tailoring, Simulation, Liking | none | Mixed | Unspecified | Behavior, Motivation, Efficacy | Children and Adults | 35 | Partially Successful | Japan |
| 150 | Lane et al.(2014), [100] | PA, Sleep, Social Interaction | Smartphone application, and ambient display on the smartphone wallpaper | BeWell+ | Tracking, Personalization, Self-Monitoring, Simulation, Rewards, Liking, Social Support (Social Interaction) | none | Quantitative | 19 Days | Behavior, Social Interaction | Unspecified (General) | 27 | Fully Successful | UK |
| 151 | Tajadura-Jimenez et al. (2018), [159] | PA | A movement wearable tracking system, Gesture-sound sensors and shoes sensors | MAGICSHOES | Tracking, Self-Monitoring, Simulation, Reminder | none | none | none | Behavior, Motivation, Confidence, Self-efficacy | Unspecified | 0 | none | Spain |

**Appendix 1. (continued)**

| **#** | **Authors of Articles, Year, Reference** | **Domain** | **Technology** | **Application / Project Name** | **Persuasive Strategies /Affordances** | **Theories** | **Evaluation Method** | **Duration** | **Targeted Outcomes** | **Audience Age Group** | **No. of Participants** | **Effectiveness / Evaluation Outcomes** | **Country of Study** |
| --- | --- | --- | --- | --- | --- | --- | --- | --- | --- | --- | --- | --- | --- |
| 152 | Hong et al.( 2013), [86] | PA | A mobile to web application (desktop version as a web, iPhone version) | iCanFit | Tracking, Reduction, Tunneling, Tailoring, Personalization, Goal Setting, Self-Monitoring, Suggestion, Praise , Trustworthiness, Expertise, Surface Credibility, Real-world Feel, Authority, Third-party Endorsements, Verifiability, Social Support ( Normative Influence, Social Interaction) | none | Mixed | 11 months | Behavior, Motivation | Elderly | 112 | Fully Successful | USA |
| 153 | Francillette et al.(2018), [55] | PA | A smartphone exergame application | An smartphone exergame app | Reduction, Tailoring, Personalization, Goal Setting, Self-Monitoring, Rewards ( Tickets), Reminder, Liking | none | Mixed | 30 minutes | Behavior, Motivation | Adults | 15 | Partially Successful | Canada |
| 154 | Dantzig et al.(2018), [37] | PA | A smartphone application , wearable activity tracker device | A digital smartphone coaching system | Tracking, Personalization, Self-monitoring, Praise, Reminder, Suggestion | none | Mixed | 1 Month | Behavior, Motivation | Adults | 70 | Partially Successful | Netherlands |
| 155 | Altmeyer et al.(2018), [6] | PA | A gamified system includes fitness tracker, mobile app, website as a public display | A gamified mobile app | Tracking, Personalization, Self-Monitoring, Rewards, Reminder, Similarity, Social Support ( Comparison, Normative Influence) | SDT | Mixed | 1 Month | Behavior, Motivation, Usability | Adults | 12 | Partially Successful | Germany |

**Appendix 1. (continued)**

| **#** | **Authors of Articles, Year, Reference** | **Domain** | **Technology** | **Application / Project Name** | **Persuasive Strategies /Affordances** | **Theories** | **Evaluation Method** | **Duration** | **Targeted Outcomes** | **Audience Age Group** | **No. of Participants** | **Effectiveness / Evaluation Outcomes** | **Country of Study** |
| --- | --- | --- | --- | --- | --- | --- | --- | --- | --- | --- | --- | --- | --- |
| 156 | Schafer et al.(2018), [148] | PA | A gamified smartphone app | A gamified smartphone app | Tracking, Personalization, Self-Monitoring, Praise, Rewards, Liking | none | Mixed | 1 Month | Behavior, Awareness, Motivation, Acceptance, Attitude | Children | 61 | Partially Successful | Germany |
| 157 | Kasteren et al.(2019), [92] | SB and PA | Fitbit tracker, and ambient temperature | Unspecified | Tracking, Self-monitoring | none | Quantitative | 2 Months | Motivation, Thermal Comfort | Adults | 15 | Partially Successful | Australia |
| 158 | Ciravegna et al.(2019), [32] | PA | Mobile phone application | Active 10 | Tracking, Reduction, Personalization, Self-monitoring, Goal-Setting, Praise, Rewards, Reminders, Expertise, Real-world feel | Unspecified | Quantitative | 8 Weeks | Behavior, Adherence | Unspecified | 129,010 | Fully Successful | UK |
| 159 | Boateng et al.(2017), [17] | PA and SB | Ambulet wrist-worn device | ActivityAware | Tracking, Reduction, Self-monitoring | none | Quantitative | 1 Months | Motivation | Obese Elderly (Participants were Young Adults 18 - 23 years old) | 14 | Fully Successful | USA |
| 160 | Oyibo et al.(2019), [131] | PA | Mobile phone application | BEN’FIT | Tailoring, Personalization, Goal-Setting, Self-Monitoring, Rewards, Social Support ( Social Learning, Social Comparison, Cooperation) | SCT | Mixed | 1 Months | Behavior, Motivation | Adults | 120 | Partially Successful | Canada, USA, and Nigeria |

**Appendix 1. (continued)**

| **#** | **Authors of Articles, Year, Reference** | **Domain** | **Technology** | **Application / Project Name** | **Persuasive Strategies /Affordances** | **Theories** | **Evaluation Method** | **Duration** | **Targeted Outcomes** | **Audience Age Group** | **No. of Participants** | **Effectiveness / Evaluation Outcomes** | **Country of Study** |
| --- | --- | --- | --- | --- | --- | --- | --- | --- | --- | --- | --- | --- | --- |
| 161 | Bascur et al.(2018), [12] | PA and Smoking | Mobile phone application | Evitapp | Tracking, Goal-Setting, Self-Monitoring, Reminders, Praise, Social Support ( Social Sharing, Social Comparison) | none | Mixed | 10 Days | Behavior, Motivation | Adults | 19 | Partially Successful | Spain |
| 162 | Samariya et al.(2019), [147] | PA | Mobile application, Wearable LED Color Light Display, Activity Tracker | KidLED mobile application | Tracking, Personalization, Goal-Setting, Self-Monitoring, Social Support (Social Learning, Social Comparison) | none | none | none | Motivation, Awareness | Children | none | none | USA |
| 163 | Oliveira et al.(2016), [128] | PA | Mobile application | PersonalFit | Tracking, Reduction, Personalization, Social Role | none | none | none | Self-management | Unspecified | none | none | Portugal |
| 164 | Economou et al.(2017), [44] | PA and Eating (Diet) | Gamified Mobile Web App | PhytoCloud | Tracking, Tailoring, Personalization, Goal-Setting, Self-Monitoring, Suggestions, Trustworthiness, Expertise, Surface Credibility, Authority, Third-Party Endorsement, Social Support ( Social Learning, Normative Influence, Recognition (Ranking), Sharing ) | none | none | none | Behavior | Adults | none | none | UK |
| 165 | Martin-Niedecken (2019), [8] | PA | Exergames (video games) | Plunder Planet | Tracking, Reduction, Tailoring, Simulation, Liking, Social Support ( Cooperation) | none | none | none | Behavior, Motivation | Children, Teenagers | none | none | Switzerland |

**Appendix 1. (continued)**

| **#** | **Authors of Articles, Year, Reference** | **Domain** | **Technology** | **Application / Project Name** | **Persuasive Strategies /Affordances** | **Theories** | **Evaluation Method** | **Duration** | **Targeted Outcomes** | **Audience Age Group** | **No. of Participants** | **Effectiveness / Evaluation Outcomes** | **Country of Study** |
| --- | --- | --- | --- | --- | --- | --- | --- | --- | --- | --- | --- | --- | --- |
| 166 | Wang and Reiterer ( 2019), [164] | SB | Computer PC System | SedentaryBar | Tracking and monitoring, Personalization, Self-Monitoring, Reminder, Surface Credibility | none | Mixed | 3 Weeks | Behavior, Awareness | Adults | 8 | Partially Successful | Germany |
| 167 | Far et al. (2014), [50] | PA | Mobile Phone apps, Web app, and sensors | Virtual Social Gym (VSG) | Tracking, Tunneling, Tailoring, Self-monitoring, Simulation, Praise, Punishment, Social Role, Surface Credibility, Social Support ( Cooperation, Comparison) | none | none | none | Motivation | Elderly | none | none | Italy |
| 168 | Geurts et al.(2019), [63] | PA | Mobile application | WalkWithMe | Tracking, Tunneling, Tailoring, Personalization, Goal-Setting, Self-Monitoring, Praise, Expertise, Social Support (Sharing) | GST | Mixed | 10 Weeks | Behavior, Motivation | Elderly | 13 | Fully Successful | Belgium |
| 169 | Spiesberger et al.(2015), [152] | PA | A gamified smartphone app | Woody | Tracking, Personalization, Simulation , Reminder (notification feedback), Rewards, Liking, Expertise | none | Mixed | 12 Days | Behavior, Awareness, Motivation | Children | 38 | Fully Successful | Austria |
| 170 | Boj et al.(2018), [18] | PA | Mobile phone application and Video games | HybridPLAY | Tracking, Simulation, Social Support ( Cooperation) | none | Mixed | 1 Month | Behavior | Elderly | 8 | Partially Successful | Spain |

References :

1. Rajwa Al-Hrathi, Ali Karime, Hussein Al-Osman, and Abdulmotaleb El Saddik. 2012. Exerlearn bike: An exergaming system for children’s educational and physical well-being. *Proceedings of the 2012 IEEE International Conference on Multimedia and Expo Workshops, ICMEW 2012*: 489–494. https://doi.org/10.1109/ICMEW.2012.91

2. Inaki Merino Albaina, Thomas Visser, Charles A.P.G. van der Mast, and Martijn H. Vastenburg. 2009. Flowie: A persuasive virtual coach to motivate elderly individuals to walk. 1–7. https://doi.org/10.4108/icst.pervasivehealth2009.5949

3. Taleb A. Alkhajah, Marina M. Reeves, Elizabeth G. Eakin, Elisabeth A.H. Winkler, Neville Owen, and Genevieve N. Healy. 2012. Sit-stand workstations: A pilot intervention to reduce office sitting time. *American Journal of Preventive Medicine*. https://doi.org/10.1016/j.amepre.2012.05.027

4. Marc Van Almkerk, Bart L. Bierling, Nono Leermakers, Jeroen Vinken, and Annick A.A. Timmermans. 2015. Improving posture and sitting behavior through tactile and visual feedback in a sedentary environment. *Proceedings of the Annual International Conference of the IEEE Engineering in Medicine and Biology Society, EMBS* 2015-Novem: 4570–4573. https://doi.org/10.1109/EMBC.2015.7319411

5. Maximilian Altmeyer, Pascal Lessel, Seyedmostafa Hosseini, and Antonio Krueger. 2018. SilverCycling: Evaluating Persuasive Strategies to Promote Physical Activity among Older Adults. In *Proceedings of the 19th International ACM SIGACCESS Conference on Computers and Accessibility - DIS ’18*. https://doi.org/10.1145/3197391.3205410

6. Maximilian Altmeyer, Pascal Lessel, Tobias Sander, and Antonio Krüger. 2018. Extending a Gamified Mobile App with a Public Display to Encourage Walking. https://doi.org/10.1145/3275116.3275135

7. Ian Anderson, Julie Maitland, Scott Sherwood, Louise Barkhuus, Matthew Chalmers, Malcolm Hall, Barry Brown, and Henk Muller. 2007. Shakra: Tracking and sharing daily activity levels with unaugmented mobile phones. *Mobile Networks and Applications* 12, 2–3: 185–199. https://doi.org/10.1007/s11036-007-0011-7

8. Anna Lisa Martin-Niedecken. 2019. Plunder Planet- An Adaptive Fitness Game Setup for Children. *CHI* : 19–30. https://doi.org/10.1145/2968120.2987720

9. Sonia M. Arteaga, Mo Kudeki, Adrienne Woodworth, and Sri Kurniawan. 2010. Mobile system to motivate teenagers’ physical activity. 1. https://doi.org/10.1145/1810543.1810545

10. Soleh U. Ayubi and Bambang Parmanto. 2012. PersonA: Persuasive social network for physical Activity. *Proceedings of the Annual International Conference of the IEEE Engineering in Medicine and Biology Society, EMBS*: 2153–2157. https://doi.org/10.1109/EMBC.2012.6346387

11. Faisal A. Barwais, Thomas F. Cuddihy, and L. M. Tomson. 2013. Physical activity, sedentary behavior and total wellness changes among sedentary adults: A 4-week randomized controlled trial. *Health and Quality of Life Outcomes* 11, 1: 1. https://doi.org/10.1186/1477-7525-11-183

12. Antonio Bascur, Pedro Rossel, Valeria Herskovic, and Claudia Martínez-Carrasco. 2018. Evitapp: Persuasive Application for Physical Activity and Smoking Cessation. *Proceedings*. https://doi.org/10.3390/proceedings2191208

13. Shlomo Berkovsky, Mac Coombe, and Richard Helmer. 2010. Activity interface for physical activity motivating games. 273. https://doi.org/10.1145/1719970.1720009

14. Shlomo Berkovsky, Jill Freyne, and Mac Coombe. 2012. Physical Activity Motivating Games: Be Active and Get Your Own Reward. *Lecture Notes in Computer Science (including subseries Lecture Notes in Artificial Intelligence and Lecture Notes in Bioinformatics)*. https://doi.org/10.1007/978-3-642-17080-5_30

15. Agon Bexheti, Anton Fedosov, Jesper Findahl, Marc Langheinrich, and Evangelos Niforatos. 2015. Re-Live the Moment: Visualizing run experiences to motivate future exercises. *Proceedings of the 17th International Conference on Human-Computer Interaction with Mobile Devices and Services Adjunct - MobileHCI ’15*: 986–993. https://doi.org/10.1145/2786567.2794316

16. Gerald Bieber, Philipp Koldrack, Christopher Sablowski, Christian Peter, and Bodo Urban. 2010. Mobile physical activity recognition of stand-up and sit-down transitions for user behavior analysis. In *Proceedings of the 3rd International Conference on PErvasive Technologies Related to Assistive Environments - PETRA ’10*. https://doi.org/10.1145/1839294.1839354

17. George Boateng, John A. Batsis, Ryan Halter, and David Kotz. 2017. ActivityAware: An app for real-time daily activity level monitoring on the Amulet wrist-worn device. In *2017 IEEE International Conference on Pervasive Computing and Communications Workshops, PerCom Workshops 2017*. https://doi.org/10.1109/PERCOMW.2017.7917601

18. Clara Boj, Diego Díaz, Cristina Portalés, and Sergio Casas. 2018. Video Games and Outdoor Physical Activity for the Elderly: Applications of the HybridPLAY Technology. *Applied Sciences*. https://doi.org/10.3390/app8101912

19. Dale S. Bond, J. Graham Thomas, Hollie A. Raynor, Jon Moon, Jared Sieling, Jennifer Trautvetter, Tiffany Leblond, and Rena R. Wing. 2014. B-MOBILE - A smartphone-based intervention to reduce sedentary time in overweight/obese individuals: A within-subjects experimental trial. *PLoS ONE* 9, 6. https://doi.org/10.1371/journal.pone.0100821

20. C. L. Brakenridge, B. S. Fjeldsoe, D. C. Young, E. A.H. Winkler, D. W. Dunstan, L. M. Straker, and G. N. Healy. 2016. Evaluating the effectiveness of organisational-level strategies with or without an activity tracker to reduce office workers’ sitting time: A cluster-randomised trial. *International Journal of Behavioral Nutrition and Physical Activity* 13, 1: 1–15. https://doi.org/10.1186/s12966-016-0441-3

21. Andreas Braun, Ingrid Schembri, and Sebastian Frank. 2015. Exerseat - Sensor-supported exercise system for ergonomic microbreaks. In *Lecture Notes in Computer Science (including subseries Lecture Notes in Artificial Intelligence and Lecture Notes in Bioinformatics)*. https://doi.org/10.1007/978-3-319-26005-1_16

22. Tim Buckers, Elmar Eisemann, and Stephan Lukosch. 2018. VRabl: Stimulating Physical Activities through a Multiplayer Augmented Reality Sports Game. https://doi.org/10.1145/3210299.3210300

23. Pradeep Buddharaju and Yokeshwaran Lokanathan. 2016. Mobile exergaming: exergames on the go. *Proceedings of the International Workshop on Mobile Software Engineering and Systems - MOBILESoft ’16*: 25–26. https://doi.org/10.1145/2897073.2897125

24. Tatjana M. Burkow, Lars K. Vognild, Elin Johnsen, Astrid Bratvold, and Marijke Jongsma Risberg. 2018. Promoting exercise training and physical activity in daily life: A feasibility study of a virtual group intervention for behaviour change in COPD. *BMC Medical Informatics and Decision Making*. https://doi.org/10.1186/s12911-018-0721-8

25. Scott A Cambo, Daniel Avrahami, and Matthew L Lee. 2017. BreakSense: Combining Physiological and Location Sensing to Promote Mobility during Work-Breaks. *Proceedings of the 2017 Acm Sigchi Conference on Human Factors in Computing Systems (Chi’17)*. https://doi.org/10.1016/j.engappai.2016.10.012

26. Lucas J. Carr, Kristina Karvinen, Mallory Peavler, Rebecca Smith, and Kayla Cangelosi. 2013. Multicomponent intervention to reduce daily sedentary time: A randomised controlled trial. *BMJ Open* 3, 10. https://doi.org/10.1136/bmjopen-2013-003261

27. Robert Cercos and Florian “Floyd” Floyd Mueller. 2013. Watch your Steps: Designing a Semi-Public Display to Promote Physical Activity. *Ie 2013*: 2. https://doi.org/10.1145/2513002.2513016

28. Anjana Chatta, Tyler Hurst, Gayani Samaraweera, Rongkai Guo, and John Quarles. 2015. Get off the Couch Get off the Couch: An Approach to Utilize Sedentary Commercial Games as Exergames. *Proceedings of the 2015 Annual Symposium on Computer-Human Interaction in Play - CHI PLAY ’15*: 47–56. https://doi.org/10.1145/2793107.2793115

29. Yong Xiang Chen, Siek Siang Chiang, Shu Yun Chih, Wen Ching Liao, Shih Yao Lin, Shang Hua Yang, Shun Wen Cheng, Shih Sung Lin, Yu Shan Lin, Ming Sui Lee, Jau Yih Tsauo, Cheng Min Jen, Chia Shiang Shih, King Jen Chang, and Yi Ping Hung. 2014. Opportunities for persuasive technology to motivate heavy computer users for stretching exercise. *Lecture Notes in Computer Science (including subseries Lecture Notes in Artificial Intelligence and Lecture Notes in Bioinformatics)* 8462 LNCS: 25–30. https://doi.org/10.1007/978-3-319-07127-5_3

30. Yu Chen and Pearl Pu. 2014. HealthyTogether: Exploring Social Incentives for Mobile Fitness Applications. *2nd International Symposium of Chinese CHI, Chinese CHI 2014*, May 2016: 25–34. https://doi.org/10.1145/2592235.2592240

31. Luca Chittaro and Riccardo Sioni. 2012. Turning the classic snake mobile game into a location-based exergame that encourages walking. *Lecture Notes in Computer Science (including subseries Lecture Notes in Artificial Intelligence and Lecture Notes in Bioinformatics)* 7284 LNCS: 43–54. https://doi.org/10.1007/978-3-642-31037-9_4

32. Fabio Ciravegna, Jie Gao, Neil Ireson, Robert Copeland, Joe Walsh, and Vitaveska Lanfranchi. 2019. Active 10- Brisk Walking to Support Regular Physical Activity. *Proceedings Paper in PervasiveHealth2019*. https://doi.org/10.1145/3329189.3329208

33. Céline Clavel, Steve Whittaker, Anaïs Blacodon, and Jean-Claude Martin. 2018. WEnner: A Theoretically Motivated Approach for Tailored Coaching about Physical Activity. In *Proceedings of the 2018 ACM International Joint Conference and 2018 International Symposium on Pervasive and Ubiquitous Computing and Wearable Computers - UbiComp ’18*. https://doi.org/10.1145/3267305.3274190

34. Drew Clinkenbeard, Jennifer Clinkenbeard, Guillaume Faddoul, Heejung Kang, Sean Mayes, Alp Toygar, and Samir Chatterjee. 2014. What’s your 2%? A pilot study for encouraging physical activity using persuasive video and social media. In *Lecture Notes in Computer Science (including subseries Lecture Notes in Artificial Intelligence and Lecture Notes in Bioinformatics)*. https://doi.org/10.1007/978-3-319-07127-5_5

35. Katrien De Cocker, Ilse De Bourdeaudhuij, Greet Cardon, and Corneel Vandelanotte. 2016. The Effectiveness of a Web-Based Computer-Tailored Intervention on Workplace Sitting: A Randomized Controlled Trial. *Journal of Medical Internet Research* 18, 5: 1–14. https://doi.org/10.2196/jmir.5266

36. Sunny Consolvo and Predrag Klasnja. 2008. Flowers or a robot army?: encouraging awareness & activity with personal, mobile displays. *Proceedings of the 10th …* 12, 4: 54–63. https://doi.org/10.1145/1409635.1409644

37. Saskia van Dantzig, Murtaza Bulut, Martijn Krans, Anouk van der Lans, and Boris de Ruyter. 2018. Enhancing physical activity through context-aware coaching. https://doi.org/10.1145/3240925.3240928

38. Saskia Van Dantzig, Gijs Geleijnse, and Aart Tijmen Van Halteren. 2013. Toward a persuasive mobile application to reduce sedentary behavior. *Personal and Ubiquitous Computing* 17, 6: 1237–1246. https://doi.org/10.1007/s00779-012-0588-0

39. Carolyn S. Dewa, Wayne deRuiter, Nancy Chau, and Kim Karioja. 2012. Walking for Wellness: Using Pedometers to Decrease Sedentary Behaviour and Promote Mental Health. *International Journal of Mental Health Promotion* 11, 2: 24–28. https://doi.org/10.1080/14623730.2009.9721784

40. Saumil Dharia, Vijesh Jain, Jvalant Patel, Jainikkumar Vora, Rizen Yamauchi, Magdalini Eirinaki, and Iraklis Varlamis. 2016. PRO-Fit: Exercise with friends. *Proceedings of the 2016 IEEE/ACM International Conference on Advances in Social Networks Analysis and Mining, ASONAM 2016*: 1430–1433. https://doi.org/10.1109/ASONAM.2016.7752437

41. Chelsea Dobbins, Paul Fergus, Madjid Merabti, and David Llewellyn-Jones. 2012. Monitoring and measuring sedentary behaviour with the aid of human digital memories. In *2012 IEEE Consumer Communications and Networking Conference, CCNC’2012*. https://doi.org/10.1109/CCNC.2012.6181016

42. Chelsea Dobbins, Madjid Merabti, Paul Fergus, and David Llewellyn-Jones. 2014. A user-centred approach to reducing sedentary behaviour. 1–6. https://doi.org/10.1109/ccnc.2014.6866645

43. Lars Donath, Oliver Faude, Yannick Schefer, Ralf Roth, and Lukas Zahner. 2015. Repetitive daily point of choice prompts and occupational sit-stand transfers, concentration and neuromuscular performance in office workers: An RCT. *International Journal of Environmental Research and Public Health* 12, 4: 4340–4353. https://doi.org/10.3390/ijerph120404340

44. Daphne Economou, Miriam Dwek, Claire Roberston, Bradley Elliott, Thanos Kounenis, Tayebeh Azimi, Mohammad Ramezanian, and Nathan Bell. 2017. PhytoCloud: A Gamified Mobile Web Application to Modulate Diet and Physical Activity of Women with Breast Cancer. In *Proceedings - IEEE Symposium on Computer-Based Medical Systems*. https://doi.org/10.1109/CBMS.2017.164

45. Andrey Esakia, D Scott Mccrickard, Samantha Harden, Michael Horning, Virginia Tech, Virginia Tech, and Virginia Tech. 2018. FitAware: Promoting Group Fitness Awareness Through Glanceable Smartwatches. *Group 2018*: 178–183. https://doi.org/10.1145/3148330.3148343

46. Rhian E. Evans, Henrietta O. Fawole, Stephanie A. Sheriff, Philippa M. Dall, P. Margaret Grant, and Cormac G. Ryan. 2012. Point-of-choice prompts to reduce sitting time at work: A randomized trial. *American Journal of Preventive Medicine* 43, 3: 293–297. https://doi.org/10.1016/j.amepre.2012.05.010

47. Anke Eyck, Kelvin Geerlings, Dina Karimova, Bernt Meerbeek, Lu Wang, Wijnand Usselsteijn, Yvonne De Kort, Michiel Roersma, and Joyce Westerink. 2006. Effect of a virtual coach on athletes’ motivation. In *Lecture Notes in Computer Science (including subseries Lecture Notes in Artificial Intelligence and Lecture Notes in Bioinformatics)*. https://doi.org/10.1007/11755494_22

48. Muhammad Fahim, Thar Baker, Asad Masood Khattak, and Omar Alfandi. 2017. Alert me: Enhancing active lifestyle via observing sedentary behavior using mobile sensing systems. *2017 IEEE 19th International Conference on e-Health Networking, Applications and Services, Healthcom 2017* 2017-Decem: 1–4. https://doi.org/10.1109/HealthCom.2017.8210838

49. Chloe Fan, Jodi Forlizzi, and Anind K. Dey. 2012. A Spark Of Activity: Exploring Information Art As Visualization For Physical Activity. In *Proceedings of the 2012 ACM Conference on Ubiquitous Computing - UbiComp ’12*, 81. https://doi.org/10.1145/2370216.2370229

50. Iman Khaghani Far, Francisco Ibarra, Marcos Baez, and Fabio Casati. 2014. *Virtual Social Gym: a Persuasive Training Platform for Independently Living Seniors*. Retrieved July 26, 2019 from http://demo.socialgym.org/

51. MJ Ferreira, AK Caraban, and E Karapanos. 2014. Breakout: predicting and breaking sedentary behaviour at work. *CHI’14 Extended Abstracts on Human Factors in Computing Systems*: 2407–2412. Retrieved from https://dl.acm.org/citation.cfm?id=2581330

52. André Fialho and Herjan Van Den Heuvel. 2009. ActiveShare : Sharing Challenges to Increase Physical Activity. *Chi’09*: 4159–4164.

53. Jutta Fortmann, Tim Stratmann, Susanne Boll, Benjamin Poppinga, and Wilko Heuten. 2013. Make Me Move at Work! An Ambient Light Display to Increase Physical Activity. https://doi.org/10.4108/icst.pervasivehealth.2013.252089

54. Derek Foster, Conor Linehan, Ben Kirman, Shaun Lawson, and Gary James. 2010. Motivating physical activity at work: Using persuasive social media for competitive step counting. *Proceedings of the 14th International Academic MindTrek Conference on Envisioning Future Media Environments - MindTrek ’10*: 111. https://doi.org/10.1145/1930488.1930510

55. Yannick Francillette, Bruno Bouchard, Eric Boucher, Sébastien Gaboury, Paquito Bernard, Ahmed Jérome Romain, and Kévin Bouchard. 2018. Development of an Exergame on Mobile Phones to Increase Physical Activity for Adults with Severe Mental Illness. https://doi.org/10.1145/3197768.3201521

56. Thomas Fritz, Elaine M. Huang, and Gail C. Murphy. 2014. Persuasive technology in the real world: a study of long-term use of activity sensing devices for fitness. *Chi*: 241–244.

57. Yuichi Fujiki, Konstantinos Kazakos, Colin Puri, Pradeep Buddharaju, Ioannis Pavlidis, and James Levine. 2008. NEAT-o-Games: Blending Physical Activity and Fun in the Daily Routine. *ACM Comput. Entertain.* 6, 1: 21. https://doi.org/10.1145/1371216.1371224

58. Kaori Fujinami and Jukka Riekki. 2008. A case study on an ambient display as a persuasive medium for exercise awareness. In *Lecture Notes in Computer Science (including subseries Lecture Notes in Artificial Intelligence and Lecture Notes in Bioinformatics)*. https://doi.org/10.1007/978-3-540-68504-3-28

59. Yue Gao, Kathrin M. Gerling, Regan L. Mandryk, and Kevin G. Stanley. 2014. Decreasing sedentary behaviours in pre-adolescents using casual exergames at school. 97–106. https://doi.org/10.1145/2658537.2658693

60. Eduardo Gasca, Jesus Favela, and Monica Tentori. 2008. Persuasive virtual communities to promote a healthy lifestyle among patients with chronic diseases. *Lecture Notes in Computer Science (including subseries Lecture Notes in Artificial Intelligence and Lecture Notes in Bioinformatics)* 5411 LNCS: 74–82. https://doi.org/10.1007/978-3-540-92831-7_7

61. Roland Gasser, Dominique Brodbeck, Markus Degen, Jürg Luthiger, Remo Wyss, and Serge Reichlin. 2006. Persuasiveness of a mobile lifestyle coaching application using social facilitation. *Lecture Notes in Computer Science (including subseries Lecture Notes in Artificial Intelligence and Lecture Notes in Bioinformatics)* 3962 LNCS: 27–38. https://doi.org/10.1007/11755494_5

62. Ben S. Gerber, Melinda R. Stolley, Allison L. Thompson, Lisa K. Sharp, and Marian L. Fitzgibbon. 2009. Mobile phone text messaging to promote healthy behaviors and weight loss maintenance: A feasibility study. *Health Informatics Journal*. https://doi.org/10.1177/1460458208099865

63. Eva Geurts, Fanny Van Geel, Peter Feys, and Karin Coninx. 2019. WalkWithMe- Personalized Goal Setting and Coaching for Walking in People with Multiple Sclerosis. In *Proceedings of the 27th ACM Conference on User Modeling, Adaptation and Personalization - UMAP ’19*, 51–60. https://doi.org/10.1145/3320435.3320459

64. Nicholas D. Gilson, Guy Faulkner, Marie H. Murphy, M. Renee Umstattd Meyer, Tracy Washington, Gemma C. Ryde, Kelly P. Arbour-Nicitopoulos, and Kimber A. Dillon. 2013. Walk@Work: An automated intervention to increase walking in university employees not achieving 10,000 daily steps. *Preventive Medicine* 56, 5: 283–287. https://doi.org/10.1016/j.ypmed.2013.01.022

65. Nicholas D. Gilson, Norman Ng, Toby G. Pavey, Gemma C. Ryde, Leon Straker, and Wendy J. Brown. 2016. Project Energise: Using participatory approaches and real time computer prompts to reduce occupational sitting and increase work time physical activity in office workers. *Journal of Science and Medicine in Sport* 19, 11: 926–930. https://doi.org/10.1016/j.jsams.2016.01.009

66. Liam G. Glynn, Patrick S. Hayes, Monica Casey, Fergus Glynn, Alberto Alvarez-Iglesias, John Newell, Gearóid ÓLaighin, David Heaney, and Andrew W. Murphy. 2013. SMART MOVE - a smartphone-based intervention to promote physical activity in primary care: Study protocol for a randomized controlled trial. *Trials*. https://doi.org/10.1186/1745-6215-14-157

67. Rúben Gouveia, Evangelos Karapanos, and Marc Hassenzahl. 2015. How Do We Engage With Activity Trackers? A Longitudinal Study of Habito. https://doi.org/10.1145/2750858.2804290

68. Rúben Gouveia, Evangelos Karapanos, and Marc Hassenzahl. 2018. Activity Tracking in vivo . https://doi.org/10.1145/3173574.3173936

69. Jasmin Grosinger, Frank Vetere, and Geraldine Fitzpatrick. 2012. Agile Life : Addressing Knowledge and Social Motivations for Active Aging. In *OZCHI*.

70. Tobias Grundgeiger, Jürgen Pichen, Jennifer Häfner, Birgit Wallmann-Sperlich, Diana Löffler, and Stephan Huber. 2017. Combating Sedentary Behavior: An App Based on a Distributed Prospective Memory Approach. *Proceedings of the 2017 CHI Conference Extended Abstracts on Human Factors in Computing Systems - CHI EA ’17*: 1632–1639. https://doi.org/10.1145/3027063.3053094

71. Xinning Gui, Yu Chen, Clara Caldeira, Dan Xiao, and Yunan Chen. 2017. When Fitness Meets Social Networks: Investigating Fitness Tracking and Social Practices on WeRun. In *Proceedings of the ACM Conference on Human Factors in Computing Systems (CHI 2017)*. https://doi.org/ccwc

72. Dario Guida, Artie Basukoski, and Performance Database. 2017. Weightbit: An Advancement in Wearable Technology. *Proceedings - IEEE Symposium on Computer-Based Medical Systems* 2017-June: 672–677. https://doi.org/10.1109/CBMS.2017.85

73. Xiaonan Guo, Jian Liu, and Yingying Chen. 2017. FitCoach: Virtual fitness coach empowered by wearable mobile devices. *Proceedings - IEEE INFOCOM*. https://doi.org/10.1109/INFOCOM.2017.8057208

74. Ankit Gupta, Tim Heng, Chris Shaw, Linda Li, and Lynne Feehan. 2018. Designing pervasive technology for physical activity self-management in arthritis patients. https://doi.org/10.1145/3240925.3240956

75. S. Gupta and S. Sood. 2015. Context aware mobile agent for reducing stress and obesity by motivating physical activity: A design approach. *International Conference on Computing for Sustainable Global Development, INDIACom 2015*: 962–966. Retrieved from https://www.scopus.com/inward/record.uri?eid=2-s2.0-84960868913&partnerID=40&md5=73862401886e0bda300f58d8b8603d97

76. Nyssa T. Hadgraft, Genevieve N. Healy, Neville Owen, Elisabeth A.H. Winkler, Brigid M. Lynch, Parneet Sethi, Elizabeth G. Eakin, Marj Moodie, Anthony D. LaMontagne, Glen Wiesner, Lisa Willenberg, and David W. Dunstan. 2016. Office workers’ objectively assessed total and prolonged sitting time: Individual-level correlates and worksite variations. *Preventive Medicine Reports* 4: 184–191. https://doi.org/10.1016/j.pmedr.2016.06.011

77. Nyssa T. Hadgraft, Lisa Willenberg, Anthony D. LaMontagne, Keti Malkoski, David W. Dunstan, Genevieve N. Healy, Marj Moodie, Elizabeth G. Eakin, Neville Owen, and Sheleigh P. Lawler. 2017. Reducing occupational sitting: Workers’ perspectives on participation in a multi-component intervention. *International Journal of Behavioral Nutrition and Physical Activity*. https://doi.org/10.1186/s12966-017-0530-y

78. Iain Hamiliton, Gennaro Imperatore, and MD Dunlop. 2012. Walk2Build: a GPS game for mobile exergaming with city visualization. *MobileHCI ’12: Proceedings of the 14th international conference on Human-computer interaction with mobile devices and services companion*: 17–22. Retrieved from http://dl.acm.org/citation.cfm?id=2371670

79. Tian Hao, Guoliang Xing, and Gang Zhou. 2015. RunBuddy: A Smartphone System for Running Rhythm Monitoring. *Proceedings of the 2015 ACM International Joint Conference on Pervasive and Ubiquitous Computing - UbiComp ’15*: 133–144. https://doi.org/10.1145/2750858.2804293

80. Md Sanaul Haque, Wali Mohammad Abdullah, Sadiqur Rahaman, Maarit Kangas, and Timo Jämsä. 2016. Persuasive health and wellbeing application: A theory-driven design in promoting physical activity. *1st International Conference on Medical Engineering, Health Informatics and Technology, MediTec 2016*. https://doi.org/10.1109/MEDITEC.2016.7835369

81. Marja Harjumaa and Harri Oinas-kukkonen. 2009. Understanding Persuasive Software Functionality in Practice : A Field Trial of Polar FT60. *Methodology*. https://doi.org/10.1145/1541948.1541952

82. Christina N Harrington, Lauren Wilcox, Wendy Rogers, and Kay Connelly. 2018. Designing Health and Fitness Apps with Older Adults : Examining the Value of Experience-Based Co-Design. In *PervasiveHealth*. https://doi.org/10.475/1145_4

83. Qian He and Emmanuel Agu. 2014. On11: An Activity Recommendation Application to Mitigate Sedentary Lifestyle. *Proceedings of the 2014 Workshop on Physical Analytics*: 3–8. https://doi.org/10.1145/2611264.2611268

84. Genevieve N. Healy, Elizabeth G. Eakin, Anthony D. LaMontagne, Neville Owen, Elisabeth A.H. Winkler, Glen Wiesner, Lynn Gunning, Maike Neuhaus, Sheleigh Lawler, Brianna S. Fjeldsoe, and David W. Dunstan. 2013. Reducing sitting time in office workers: Short-term efficacy of a multicomponent intervention. *Preventive Medicine*. https://doi.org/10.1016/j.ypmed.2013.04.004

85. Sen H. Hirano, Robert G. Farrell, Catalina M. Danis, and Wendy A. Kellogg. 2013. WalkMinder: Encouraging an Active Lifestyle Using Mobile Phone Interruptions. *Chi Ea 2013*: 1431–1436. https://doi.org/10.1145/2468356.2468611

86. Yan Hong, Deborah Vollmer Dahlke, Marcia Ory, Angela Hochhalter, Jana Reynolds, Ninfa Pena Purcell, Divya Talwar, and Nola Eugene. 2013. Designing icanfit: A mobile-enabled web application to promote physical activity for older cancer survivors. *Journal of Medical Internet Research*. https://doi.org/10.2196/resprot.2440

87. Yitong Huang. 2016. How to Design Internet of Things to Encourage Office Workers to Take More Regular Micro-Breaks. 1–3. https://doi.org/10.1145/2970930.2970963

88. Nassim Jafarinaimi, Jodi Forlizzi, Amy Hurst, and John Zimmerman. 2004. Breakaway: An ambient display designed to change human behavior. BT - In Proceedings of the Conference on Computer-Human Interaction. 1945–1948.

89. Russell Jago, Simon J. Sebire, Katrina M. Turner, Georgina F. Bentley, Joanna K. Goodred, Kenneth R. Fox, Sarah Stewart-Brown, and Patricia J. Lucas. 2013. Feasibility trial evaluation of a physical activity and screen-viewing course for parents of 6 to 8 year-old children: Teamplay. *International Journal of Behavioral Nutrition and Physical Activity*. https://doi.org/10.1186/1479-5868-10-31

90. Younbo Jung, Koay Jing Li, Ng Sihui Janissa, Wong Li Chieh Gladys, and Kwan Min Lee. 2009. Games for a better life: Effects of playing wii games on the well-being of seniors in a long-term care facility BT - 6th Australasian Conference on Interactive Entertainment, IE 2009, December 17, 2009 - December 19, 2009. University of Technology Sydney; ACM Special Inter. https://doi.org/10.1145/1746050.1746055

91. Ali Karime, Basim Hafidh, Abdulmajeed Khaldi, Jihad Mohamad Aljaam, and Abdulmotaleb El Saddik. 2012. MeMaPads: Enhancing children’s well-being through a physically interactive memory and math games. *2012 IEEE I2MTC - International Instrumentation and Measurement Technology Conference, Proceedings*: 2563–2566. https://doi.org/10.1109/I2MTC.2012.6229520

92. Yasmin van Kasteren, Stephanie Champion, Lua Perimal-Lewis, and Jennifer B Sartor. 2019. Thermal comfort and physical activity in an office setting. https://doi.org/10.1145/3290688.3290733

93. Christine Keung, Alexa Lee, Megan O’Keefe, and Shirley Lu. 2013. BunnyBolt: A Mobile Fitness App for Youth. *Idc’13*: 585–588.

94. Ashraf Khalil and Salam Abdallah. 2013. Harnessing social dynamics through persuasive technology to promote healthier lifestyle. *Computers in Human Behavior* 29, 6: 2674–2681. https://doi.org/10.1016/j.chb.2013.07.008

95. Julie A. Kientz, Eun Kyoung Choe, Brennen Birch, Robert Maharaj, Amanda Fonville, Chelsey Glasson, and Jen Mundt. 2010. Heuristic evaluation of persuasive health technologies. https://doi.org/10.1145/1882992.1883084

96. Joohee Kim, Na Hyeon Lee, Byung-Chull Bae, and Jun Dong Cho. 2016. A Feedback System for the Prevention of Forward Head Posture in Sedentary Work Environments. 161–164. https://doi.org/10.1145/2908805.2909414

97. Michel C.A. Klein, Adnan Manzoor, and Julia S. Mollee. 2017. Active2Gether: A personalized m-health intervention to encourage physical activity. *Sensors (Switzerland)* 17, 6: 1–16. https://doi.org/10.3390/s17061436

98. Rakesh Kumar, Alec Bayliff, Debraj De, Adam Evans, Sajal K. Das, and Mignon Makos. 2016. Care-Chair: Sedentary Activities and Behavior Assessment with Smart Sensing on Chair Backrest. *2016 IEEE International Conference on Smart Computing, SMARTCOMP 2016*: 1–8. https://doi.org/10.1109/SMARTCOMP.2016.7501682

99. Joyca Lacroix, Privender Saini, and Annelies Goris. 2009. Understanding user cognitions to guide the tailoring of persuasive technology-based physical activity interventions. 1. https://doi.org/10.1145/1541948.1541961

100. Nicholas D. Lane, Mu Lin, Mashfiqui Mohammod, Xiaochao Yang, Hong Lu, Giuseppe Cardone, Shahid Ali, Afsaneh Doryab, Ethan Berke, Andrew T. Campbell, and Tanzeem Choudhury. 2014. BeWell: Sensing sleep, physical activities and social interactions to promote wellbeing. *Mobile Networks and Applications*. https://doi.org/10.1007/s11036-013-0484-5

101. Andrea Leal Penados, Mathieu Gielen, Pieter Jan Stappers, and Tinus Jongert. 2010. Get up and move: An interactive cuddly toy that stimulates physical activity. *Personal and Ubiquitous Computing* 14, 5: 397–406. https://doi.org/10.1007/s00779-009-0270-3

102. Daehyoung Lee, Georgia Frey, Alison Cheng, and Patrick C. Shih. 2018. Puzzle walk: A gamified mobile app to increase physical activity in adults with autism spectrum disorder. In *2018 10th International Conference on Virtual Worlds and Games for Serious Applications, VS-Games 2018 - Proceedings*. https://doi.org/10.1109/VS-Games.2018.8493439

103. BY Lim, Aubrey Shick, Chris Harrison, and SE Hudson. 2011. Pediluma: motivating physical activity through contextual information and social influence. *Proceedings of the fifth …*: 173–180. Retrieved from http://dl.acm.org/citation.cfm?id=1935736

104. James J. Lin, Lena Mamykina, Henry B. Strub, Silvia Lindtner, and Gregory Delajoux. 2006. Fish’n’Steps: Encouraging Physical Activity with an Interactive Computer Game. 261–278. https://doi.org/10.1007/11853565_16

105. Yuzhong Lin, Joran Jessurun, Bauke de Vries, and Harry Timmermans. 2011. Motivate: towards Context-Aware Recommendation Mobile System for Healthy Living. 250–253. https://doi.org/10.4108/icst.pervasivehealth.2011.246030

106. Yuhan Luo, Bongshin Lee, Donghee Yvette Wohn, Amanda L. Rebar, David E. Conroy, and Eun Kyoung Choe. 2018. Time for Break: Understanding Information Workers’ Sedentary Behavior Through a Break Prompting System. *Proceedings of the 2018 CHI Conference on Human Factors in Computing Systems - CHI ’18*: 1–14. https://doi.org/10.1145/3173574.3173701

107. Andrew Macvean and Judy Robertson. 2013. Understanding exergame users’ physical activity, motivation and behavior over time. 1251. https://doi.org/10.1145/2470654.2466163

108. Chiraphruet Mansart, Siriluck Sukitphittayanon, Panitan Pantongkhum, and Supphachai Thaicharoen. 2015. Go Run Go: An Android Game-Story Application for Aiding Motivation to Exercise. *Proceedings - 2015 IEEE International Symposium on Multimedia, ISM 2015*: 407–410. https://doi.org/10.1109/ISM.2015.49

109. Gabriela Marcu, Anjali Misra, Karina Caro, Meghan Plank, Amy Leader, and Andrea Barsevick. 2018. Bounce: Designing a Physical Activity Intervention for Breast Cancer Survivors. https://doi.org/10.1145/nnnnnnn.nnnnnnn

110. Catherine Marinac, Gina Merchant, Suneeta Godbole, Jacqueline Chen, Jacqueline Kerr, Bronwyn Clark, and Simon Marshall. 2013. The feasibility of using SenseCams to measure the type and context of daily sedentary behaviors. https://doi.org/10.1145/2526667.2526674

111. Claudine McCreadie, Jonathan Raper, Anil Gunesh, Jo Wood, Kevin Carey, Helen Petrie, Lucy Wood, Ordnance Survey, Steve Tyler, and Simon Biggs. 2006. Persuasive technology for leisure and health: Development of a personal navigation tool. *Lecture Notes in Computer Science (including subseries Lecture Notes in Artificial Intelligence and Lecture Notes in Bioinformatics)* 3962 LNCS: 187–190. https://doi.org/10.1007/11755494_27

112. Siobhan McMahon, Mithra Vankipuram, and Julie Fleury. 2013. Mobile Computer Application for Promoting Physical Activity. *Journal of Gerontological Nursing*. https://doi.org/10.3928/00989134-20130226-01

113. Jochen Meyer, Elke Beck, Kai von Holdt, Dirk Gansefort, Tilman Brand, Hajo Zeeb, and Susanne Boll. 2018. ActiStairs: Design and Acceptance of a Technology-Based Intervention to Advocate Stair-Climbing in Public Spaces. *Proceedings of the 3rd International Workshop on Multimedia for Personal Health and Health Care*: 59–66.

114. Deedee A. Min, Keun Young Kim, Yaejin Kim, Su-Eun Jung, Sung A Jang, and Ji-Hyun Lee. 2015. Pretty Pelvis: A Virtual Pet Application That Breaks Sedentary Time by Promoting Gestural Interaction. *Crossing: Proceedings of CHI ’15 Extended Abstracts on Human Factors in Computing Systems*: 1259–1264. https://doi.org/10.1145/2702613.2732807

115. Mohammad Moghimi, Wanmin Wu, Jacqueline Chen, and Suneeta Godbole. 2014. ANALYZING SEDENTARY BEHAVIOR IN LIFE-LOGGING IMAGES. *International Conference on Image Processing(ICIP)*: 1011–1015.

116. Hazwani Mohd Mohadis and Nazlena Mohamad Ali. 2016. Designing persuasive application to encourage physical activity at workplace among older workers. In *2016 6th International Conference on Digital Information and Communication Technology and Its Applications, DICTAP 2016*. https://doi.org/10.1109/DICTAP.2016.7544013

117. Florian Mueller, Stefan Agamanolis, and Rosalind Picard. 2003. Exertion interfaces: Sports over a distance for social bonding and fun. *Proceedings of the Conference on Human Factors in Computing Systems*, 5: 561–568. https://doi.org/10.1145/642611.642709

118. Florian “Floyd” Mueller, Martin R. Gibbs, and Frank Vetere. 2009. Design influence on social play in distributed exertion games. 1539. https://doi.org/10.1145/1518701.1518938

119. Aarón Munguía and PC Santana. 2010. A wearable augmented reality system to improve the quality of life of sedentary people. *Proceedings of the 3rd …* 1: 8–11. Retrieved from http://www.pedrosantana.mx/papers/MexIHC-2010_augmented-reality.pdf

120. Sean Munson and Sunny Consolvo. 2012. Exploring Goal-setting, Rewards, Self-monitoring, and Sharing to Motivate Physical Activity. https://doi.org/10.4108/icst.pervasivehealth.2012.248691

121. Adity Mutsuddi and Kay Connelly. 2012. Text Messages for Encouraging Physical Activity Are they effective after the novelty effect wears off? *2012 6th International Conference on Pervasive Computing Technologies for Healthcare (PervasiveHealth) and Workshops*: 33–40. https://doi.org/10.4108/icst.pervasivehealth.2012.248715

122. Sanjay Nair, Matin Kheirkhahan, Anis Davoudi, Parisa Rashidi, Amal A. Wanigatunga, Duane B. Corbett, Todd M. Manini, and Sanjay Ranka. 2016. ROAMM: A software infrastructure for real-time monitoring of personal health. *2016 IEEE 18th International Conference on e-Health Networking, Applications and Services, Healthcom 2016*. https://doi.org/10.1109/HealthCom.2016.7749479

123. Tatsuo Nakajima and Vili Lehdonvirta. 2013. Designing motivation using persuasive ambient mirrors. *Personal and Ubiquitous Computing* 17, 1: 107–126. https://doi.org/10.1007/s00779-011-0469-y

124. Satoshi Nakamura, Mitsuru Minakuchi, and Katsumi Tanaka. 2005. Energy browser: to make exercise enjoyable and interesting. In *Proceedings of the 2005 ACM SIGCHI International Conference on Advances in computer entertainment technology*. https://doi.org/http://doi.acm.org/10.1145/1178477.1178521

125. Maike Neuhaus, Genevieve N. Healy, David W. Dunstan, Neville Owen, and Elizabeth G. Eakin. 2014. Workplace sitting and height-adjustable workstations: A randomized controlled trial. *American Journal of Preventive Medicine*. https://doi.org/10.1016/j.amepre.2013.09.009

126. Christoph Obermair, Wolfgang Reitberger, Alexander Meschtscherjakov, Michael Lankes, and Manfred Tscheligi. 2008. PerFrames: Persuasive picture frames for proper posture. In *Lecture Notes in Computer Science (including subseries Lecture Notes in Artificial Intelligence and Lecture Notes in Bioinformatics)*. https://doi.org/10.1007/978-3-540-68504-3-12

127. Rodrigo De Oliveira and Nuria Oliver. 2008. Triplebeat: enhancing exercise performance with persuasion. *In Mobile {HCI}*: 255–264.

128. T. Oliveira, D. Leite, and G. Marreiros. 2016. PersonalFit - Fitness App with intelligent plan generator. In *ACM International Conference Proceeding Series*. https://doi.org/10.1145/2948992.2949014

129. T. Ornelas, A. Caraban, R. Gouveia, and E. Karapanos. 2015. CrowdWalk: Leveraging the wisdom of the crowd to inspire walking activities. *UbiComp*: 213–216. https://doi.org/10.1145/2800835.2800923

130. Jennifer J. Otten, Katherine E. Jones, Benjamin Littenberg, and Jean Harvey-Berino. 2009. Effects of television viewing reduction on energy intake and expenditure in overweight and obese adults: A randomized controlled trial. *Archives of Internal Medicine*. https://doi.org/10.1001/archinternmed.2009.430

131. Kiemute Oyibo, Abdul-Hammid Olagunju, Babatunde Olabenjo, Ifeoma Adaji, Ralph Deters, and Julita Vassileva. 2019. BEN’FIT: Design, Implementation and Evaluation of a Culture-Tailored Fitness App. https://doi.org/10.1145/3314183.3323854

132. Taiwoo Park, Uichin Lee, Bupjae Lee, Haechan Lee, Sanghun Son, Seokyoung Song, and Junehwa Song. 2013. ExerSync: Facilitating interpersonal synchrony in social exergames. *Proceedings of the 2013 conference on Computer supported cooperative work - CSCW ’13*: 409. https://doi.org/10.1145/2441776.2441823

133. Taiwoo Park, Junehwa Song, Inseok Hwang, Uichin Lee, Sunghoon Ivan Lee, Chungkuk Yoo, Youngki Lee, Hyukjae Jang, Sungwon Peter Choe, and Souneil Park. 2012. ExerLink: enabling pervasive social exergames with heterogeneous exercise devices. *Proceedings of the 10th international conference on Mobile systems, applications, and services - MobiSys ’12*: 15. https://doi.org/10.1145/2307636.2307639

134. Parry, S. and Straker, L. 2013. The contribution of office work to sedentary behaviour associated risk. *BMC Public Health* 13, 1. https://doi.org/10.1186/1471-2458-13-296

135. Michel Peeters, Aarnout Brombacher, Carl Megens, Elise van den Hoven, and Caroline Hummels. 2013. Social Stairs: taking the Piano Staircase towards long- term behavioral change. Figure 1: 1–6. Retrieved from https://link.springer.com/content/pdf/10.1007/978-3-642-37157-8_21.pdf

136. Christine A. Pellegrini, Sara A. Hoffman, Elyse R. Daly, Manuel Murillo, Gleb Iakovlev, and Bonnie Spring. 2015. Acceptability of smartphone technology to interrupt sedentary time in adults with diabetes. *Translational Behavioral Medicine*. https://doi.org/10.1007/s13142-015-0314-3

137. Laura Pina, Ernesto Ramirez, and William Griswold. 2012. Fitbit+: A behavior-based intervention system to reduce sedentary behavior. https://doi.org/10.4108/icst.pervasivehealth.2012.248761

138. Kathrin Probst, D Lindlbauer, and P Greindl. 2013. Rotating, tilting, bouncing: using an interactive chair to promote activity in office environments. *CHI ’13 Extended Abstracts on Human Factors in Computing Systems*: 79–84. https://doi.org/10.1145/2468356.2468372

139. Kathrin Probst, Florian Perteneder, Leitner Jakob, Michael Haller, Andreas Schrempf, and Josef Glöckl. 2012. Active Office : Towards an Activity- Promoting Office Workplace Design. *Extended Abstracts on Human Factors in Computing Systems (CHI’12)*: 2165–2170.

140. Nicolaas P. Pronk, Abigail S. Katz, Marcia Lowry, and Jane Rodmyre Payfer. 2012. Reducing Occupational Sitting Time and Improving Worker Health: The Take-a-Stand Project, 2011. *Preventing Chronic Disease* 9, 8: 1–9. https://doi.org/10.5888/pcd9.110323

141. Anna Puig-Ribera, Iván Martínez-Lemos, Raimon Milà, Maria Giné-Garriga, Nicholas D. Gilson, Judit Bort-Roig, Josep Fortuño, Angel M. González-Suárez, Joan C. Martori, Jim McKenna, and Laura Muñoz-Ortiz. 2016. Patterns of Impact Resulting from a ‘Sit Less, Move More’ Web-Based Program in Sedentary Office Employees. *Plos One* 10, 4: e0122474. https://doi.org/10.1371/journal.pone.0122474

142. Nithya Ramanathan, Faisal Alquaddoomi, Hossein Falaki, Dony George, Cheng-Kang Hsieh, John Jenkins, Cameron Ketcham, Brent Longstaff, Jeroen Ooms, Joshua Selsky, Hongsuda Tangmunarunkit, and Deborah Estrin. 2012. ohmage: An open Mobile System for Activity and Experience Sampling. https://doi.org/10.4108/icst.pervasivehealth.2012.248705

143. X Ren, R Brankaert, V Visser, S Offermans, Y Lu, and H Nagtzaam. 2016. FLOW pillow: Exploring sitting experience towards active ageing. *Proceedings of the 18th International Conference on Human-Computer Interaction with Mobile Devices and Services Adjunct, MobileHCI 2016*: 706–713. https://doi.org/10.1145/2957265.2961841

144. Ian Renfree and Anna Cox. 2016. Tangibly Reducing Sedentariness in Office Workers. *Proceedings of ACM CHI 2016*. Retrieved from https://tangibles4health.files.wordpress.com/2016/02/ian-renfree-anna-cox-tangibles-for-health-workshop-chi-2016-camera-ready.pdf

145. Marcela D. Rodríguez, José R. Roa, Alberto L. Morán, and Sandra Nava-Muñoz. 2013. CAMMInA: A mobile ambient information system to motivate elders to exercise. *Personal and Ubiquitous Computing* 17, 6: 1127–1134. https://doi.org/10.1007/s00779-012-0561-y

146. Ryo Sakai, Sarah Van Peteghem, Leoni Van De Sande, Peter Banach, and Maurits Kaptein. 2011. Personalized persuasion in ambient intelligence: The APStairs system. *Lecture Notes in Computer Science (including subseries Lecture Notes in Artificial Intelligence and Lecture Notes in Bioinformatics)* 7040 LNCS: 205–209. https://doi.org/10.1007/978-3-642-25167-2_26

147. Ankita Samariya, Anud Sharma, Margiawan Fitriani, Tucker Ferguson, Jerry Alan Fails, and Jerry Alan. 2019. KidLED: A colorful approach to children’s activity awareness. https://doi.org/10.1145/3311927.3326594

148. Hanna Schäfer, Joachim Bachner, Sebastian Pretscher, Georg Groh, and Yolanda Demetriou. 2018. Study on Motivating Physical Activity in Children with Personalized Gamified Feedback. https://doi.org/10.1145/3213586.3225227

149. Jurgen van Schagen, Martijn Gribnau, Jean de Leeuw, Benjamin Los, Nick Cleintuar, and Rafael Bidarra. 2015. Super Starfish Mania: Fish for Friends. *Proceedings of the 2015 Annual Symposium on Computer-Human Interaction in Play - CHI PLAY ’15*: 787–790. https://doi.org/10.1145/2793107.2810275

150. Sarah Justine Guy Skriloff, Dario C Gonzalez, Kurtis C Christensen, Logan J Bentley, and Cody V Mortensen. 2016. FitPlay Games: Increasing Exercise Motivation Through Asynchronous Social Gaming. *Proceedings of the 2016 CHI Conference Extended Abstracts on Human Factors in Computing Systems*. https://doi.org/10.1145/2851581.2890367

151. M Sohn and Jeunwoo Lee. 2007. UP health: Ubiquitously Persuasive Health Promotion with an Instant Messaging System. *25th SIGCHI Conference on Human Factors in Computing Systems 2007, CHI 2007*: 2663–2668. https://doi.org/10.1145/1240866.1241059

152. P. Spiesberger, F. Jungwirth, C. Wöss, S. Bachl, J. Harms, and T. Grechenig. 2015. Woody: A location-based smartphone game to increase children’s outdoor activities in urban environments. In *ACM International Conference Proceeding Series*. https://doi.org/10.1145/2836041.2841210

153. Richard Spinney, Lee Smith, Marcella Ucci, Abigail Fisher, Marina Konstantatou, Alexia Sawyer, Jane Wardle, and Alexi Marmot. 2015. Indoor tracking to understand physical activity and sedentary behaviour: Exploratory study in UK office buildings. *PLoS ONE* 10, 5: 1–19. https://doi.org/10.1371/journal.pone.0127688

154. Donna Spruijt-Metz, Selena T. Nguyen-Michel, Michael I. Goran, Chih Ping Chou, and Terry T.K. Huang. 2008. Reducing sedentary behavior in minority girls via a theory-based, tailored classroom media intervention. *International Journal of Pediatric Obesity*. https://doi.org/10.1080/17477160802113415

155. K G Stanley, I Livingston, A Bandurka, R Kapiszka, and R L Mandryk. 2010. PiNiZoRo: A GPS-based exercise game for families. *International Academic Conference on the Future of Game Design and Technology, Future Play 2010*: 243–246. https://doi.org/10.1145/1920778.1920817

156. Jeroen Stragier, Tom Evens, and Peter Mechant. 2015. Broadcast yourself: An exploratory study of sharing physical activity on social networking sites. *Media International Australia*.

157. Mingui Sun, Lora E Burke, Zhi-hong Mao, Yiran Chen, Hsin-Chen Chen, Yicheng Bai, Yuecheng Li, Chengliu Li, and Wenyan Jia. 2014. eButton : A Wearable Computer for Health Monitoring and Personal Assistance. *Design Automation Conference (DAC), 2014 51st ACM/EDAC/IEEE*: 1–6. https://doi.org/10.1145/2593069.2596678

158. J. Synnott, J. Rafferty, and C. D. Nugent. 2016. Detection of workplace sedentary behavior using thermal sensors. *Proceedings of the Annual International Conference of the IEEE Engineering in Medicine and Biology Society, EMBS* 2016-Octob: 5413–5416. https://doi.org/10.1109/EMBC.2016.7591951

159. Ana Tajadura-Jiménez, Francisco Cuadrado, Patricia Rick, Nadia Bianchi-Berthouze, Aneesha Singh, Aleksander Väljamäe, and Frédéric Bevilacqua. 2018. Designing a gesture-sound wearable system to motivate physical activity by altering body perception. https://doi.org/10.1145/3212721.3212877

160. W C Taylor, R J Paxton, R Shegog, S P Coan, A Dubin, T F Page, and D M Rempel. 2016. Impact of Booster Breaks and Computer Prompts on Physical Activity and Sedentary Behavior Among Desk-Based Workers: a Cluster-Randomized Controlled Trial. *Preventing chronic disease*. https://doi.org/10.5888/pcd13.160231

161. Tammy Toscos, Anne Faber, Shunying An, and Mona Praful Gandhi. 2006. Chick Clique- Persuasive Technology to Motivate Teenage Girls to Exercise. https://doi.org/10.1145/1125451.1125805

162. Tammy Toscos, Anne Faber, Kay Connelly, and Adity Mutsuddi Upoma. 2008. Encouraging physical activity in teens can technology help reduce barriers to physical activity in adolescent girls? In *Proceedings of the 2nd International Conference on Pervasive Computing Technologies for Healthcare 2008, PervasiveHealth*. https://doi.org/10.1109/PCTHEALTH.2008.4571073

163. Joyan Urda, Jeffrey Lynn, and Beth Larouere. 2016. An Intervention To Reduce Sedentary Time And Change Perceived Wellness In Women Office Workers. *Medicine & Science in Sports & Exercise*. https://doi.org/10.1249/01.mss.0000486785.36151.36

164. Yunlong Wang and Harald Reiterer. 2019. The Point-of-Choice Prompt or the Always-On Progress Bar?: A Pilot Study of Reminders for Prolonged Sedentary Behavior Change. https://doi.org/10.1145/3290607.3313050

165. Matthias Wölfel. 2017. Acceptance of dynamic feedback to poor sitting habits by anthropomorphic objects. https://doi.org/10.1145/3154862.3154928

166. Mark Mingyi Young. 2010. Twitter me: Using micro-blogging to motivate teenagers to exercise. *Lecture Notes in Computer Science (including subseries Lecture Notes in Artificial Intelligence and Lecture Notes in Bioinformatics)* 6105 LNCS: 439–448. https://doi.org/10.1007/978-3-642-13335-0_30

167. Kazi I. Zaman, Sami Yli-Piipari, and Timothy W. Hnat. 2014. Kinematic-based sedentary and light-intensity activity detection for wearable medical applications. 28–33. https://doi.org/10.1145/2676431.2676433

168. Jingwen Zhang and John B. Jemmott III. 2019. Mobile App-Based Small-Group Physical Activity Intervention for Young African American Women: a Pilot Randomized Controlled Trial. *Prevention Science*: 1–10. https://doi.org/10.1007/s11121-019-01006-4

169. Oren Zuckerman and Ayelet Gal-Oz. 2014. Deconstructing gamification: evaluating the effectiveness of continuous measurement, virtual rewards, and social comparison for promoting physical activity. *Personal and Ubiquitous Computing* 18, 7: 1705–1719. https://doi.org/10.1007/s00779-014-0783-2

170. Matthijs Jan Zwinderman, Azadeh Shirzad, Xinyu Ma, Prina Bajracharya, Hans Sandberg, and Maurits Clemens Kaptein. 2012. Phone row: A smartphone game designed to persuade people to engage in moderate-intensity physical activity. *Lecture Notes in Computer Science (including subseries Lecture Notes in Artificial Intelligence and Lecture Notes in Bioinformatics)* 7284 LNCS: 55–66. https://doi.org/10.1007/978-3-642-31037-9_5
